# Social capital and resilience among people living on antiretroviral therapy in resource-poor Uganda

**DOI:** 10.1371/journal.pone.0197979

**Published:** 2018-06-11

**Authors:** Esther Kalule Nanfuka, David Kyaddondo, Sarah N. Ssali, Narathius Asingwire

**Affiliations:** 1 Department of Social Work and Social Administration, Makerere University, Kampala, Uganda; 2 School of Gender and Women Studies, Makerere University, Kampala, Uganda; The Ohio State University, UNITED STATES

## Abstract

**Background:**

Despite the national roll-out of free HIV medicines in Uganda and other sub-Saharan African countries, many HIV positive patients on antiretroviral therapy (ART) are at risk of non-adherence due to poverty and other structural and health system related constraints. However, several patients exhibit resilience by attaining and sustaining high levels of adherence amid adversity. Social capital, defined as resources embedded within social networks, is key in facilitating resilience but the mechanism through which it operates remains understudied. This article provides insights into mechanisms through which social capital enables patients on ART in a resource-poor setting to overcome risk and sustain adherence to treatment.

**Methodology:**

The article draws from an ethnographic study of 50 adult male and female HIV patients enrolled at two treatment sites in Uganda, 15 of whom were followed-up for an extended period of six months for narrative interviews and observation. The patients were selected purposively on the basis of socio-demographic and treatment related criteria.

**Findings:**

Social capital protects patients on ART against the risk of non-adherence in three ways. 1) It facilitates access to scarce resources; 2) encourages HIV patients to continue on treatment; and 3) averts risk for non-adherence.

**Conclusions:**

Social capital is a key resource that can be harnessed to promote resilience among HIV patients in a resource-limited setting amid individual, structural and health system related barriers to ART adherence. Invigoration and maintenance of collectivist norms may however be necessary if its protective benefits are to be fully realized.

## Introduction

Social capital is linked directly and indirectly to health outcomes [1]. By social capital, we mean the resources embedded and accessed through informal direct and indirect ties in social networks [2, 3]. Several studies have associated access to social capital with improved and sustained adherence to antiretroviral therapy (ART) in resource-poor settings.

In a study of three African countries, Nigeria, Tanzania and Uganda, Ware et al [4] report how HIV patients overcame economic obstacles to adherence with the help of family, friends and health care providers. They describe how a nominated treatment partner borrowed food from a neighbour who owned a shop to help an ill patient cope with treatment [pg 0043]. In their inquiry about the supportive strategies that would encourage the highest rates of adherence among HIV patients in the Western Cape South Africa, Nachega and colleagues [5] cite the patient’s ability to build a good supportive network outside the clinic as crucial for adherence in the long term. In Uganda, having treatment supporters was found to improve adherence to HIV treatment. Kunutsor and colleagues [6] found that ART clients with treatment supporters were more likely to achieve 95% adherence, be on time for their clinic appointments and less likely to skip appointments. These positive outcomes were attributed to the constant reminders about appointments, financial and moral support extended by nominated treatment supporters. In Tanzania O’Laughlin et al [7] report roles performed by nominated treatment supporters to include not only promoting adherence to antiretroviral medicines but also the social health of patients by proactively minimizing the isolating effects of HIV-related stigma and ill-health. This enabled people living with HIV (PLHIV) to access vital community support, such as short term-credit from lending associations, which was helpful in keeping them on treatment.

Accordingly, social capital is suggested as a key facilitator of resilience among PLHIV on ART in sub-Saharan Africa [7, 8]. Borrowing from Olsson and colleagues [9] we take resilience to mean the ability of patients on ART to surmount risk posed by psychological, social and economic barriers to sustain adherence to HIV treatment. However, the mechanism through which social capital protects HIV patients from risk of non-adherence remains under studied. Yet such understanding is necessary if social capital is to be harnessed to promote resilience among HIV patients on ART. Going beyond the broad presentation of social capital [7, 8], in this article we analyze how bonding, bridging and linking networks from which it is accessed [10] contribute to the resilience of patients on ART.

Sustained adherence to HIV treatment in Uganda and other parts of sub-Saharan Africa is indicated to be primarily constrained by structural and health system related barriers. In their review of the status of knowledge and research priorities on ART adherence and retention in care in low and middle income countries Nachega and colleagues [11] observe that while HIV patients worldwide encounter social, economic and individual barriers to adherence; structural factors are the most important in resource limited settings. They refer to a systematic review of patient reported barriers and facilitators in developed and developing countries [12] which identifies cost, non-disclosure, the fear of being stigmatized, alcohol abuse, transportation difficulties and pharmacy stock-outs as the most important and frequently cited barriers to adherence in developing countries. A study of adherence problems in Uganda, Tanzania and Botswana found that patients in the three countries were generally committed to taking their HIV medicines as prescribed, but were constrained by transport costs, user fee charges, long waiting time, stigma and inadequate food and information on the side effects of the medicines [13]. More recent studies reveal that ART adherence among HIV patients in Uganda continues to be imperiled by financial constraints, food insecurity, stock-outs of antiretroviral and medicines for the prevention and treatment of opportunistic infections, routine transport costs to the treatment centres and HIV-related stigma [14–17].

This article provides insights in to the protective mechanisms of social capital in light of promoting resilience among HIV patients on ART in resource constrained Uganda. Written from a strengths perspective [18] it demonstrates how bonding, bridging and linking networks enable PLHIV on medicine in a peri-urban district of central Uganda to beat specific structural and health system related constraints to adhere to HIV medicines and other requirements of the treatment regime. The article provides context specific recommendations for enhancing resilience among HIV patients that can be drawn on by other resource-limited settings.

## Methods

### Study design

The paper draws on data from a qualitative study that examined how PLHIV on ART mobilize social and medical resources for the day today management of HIV. The study employed an ethnographic approach with the aim of generating a thick description of events, actions, processes and systems of meanings [19] to understand the patients’ practices of mobilizing resources for the management of HIV.

Data were collected at two ART sites in Mukono district, central Uganda. Naggalama Hospital is a mission (private not for profit) facility located in the rural part of Mukono. On the other hand Mukono Health Centre IV (HCIV) is an urban public health facility in the heart of Mukono Municipality. Whilst both facilities provide free HIV medicines under the national ART scale-up arrangement, their varied management approaches pose differences in health system related risks and opportunities.

### Sampling

Study participants were selected purposively on the basis of age (18 years and above), gender, marital and disclosure status, length of period on ART, distance from the treatment centre, number of people on ART in the household and whether on antiretrovirals or only Septrin/Dapsone and suffering from an illness or not. Initially 50 patients (25 from either facility) were selected and interviewed. From the fifty, 15 cases (10 women and 5 men) were identified for extended follow-up. These were selected on the basis of unique attributes like advanced age, total dependence, several people in the household on ART, living far from the treatment centre, serious illness, discordance and non-disclosure to significant others like spouses. Clients who met our criteria were identified and recruited for the study during routine visits to the two treatment centres, in most cases with the help of health workers. Data in this article are based on both the experiences of the 50 patients and others we encountered as we participated in the activities of routine ART clinics.

### Data collection

The study protocol and data collection tools were designed collaboratively by all the four authors, but data were mainly collected by Esther through semi- structured and narrative interviews, extended case method and participant observation. At the beginning of the study the 50 respondents were engaged in semi- structured interviews at the treatment centres, which lasted approximately between one and two hours. The semi-structured interviews were conducted with the help of a guide comprising open-ended questions. The questions captured key socio-demographic characteristics of the respondents including length of period on ART, occupation and residence, the resources they considered important in the management of ART, how they mobilized them, the constraints they encountered in the mobilization process, how they overcame them and the relations that were particularly supportive in this regard. The semi-structured interviews enabled us to gain a broad understanding of the respondents’ HIV illness experiences [20]. Data from the semi-structured interviews informed the selection of the 15 respondents for extended follow-up. These were visited in their homes monthly, for a period of six months, from September 2015 to February 2016, but we kept in touch beyond this period. While in their homes, the patients and, in some cases their significant others, were engaged in narrative interviews to gain deeper insights in to their experiences on ART. The narratives were guided to follow-up issues of interest that had been identified from preliminary transcription and coding of emerging themes from earlier interviews and to capture new developments in patients’ lives regarding mobilization of resources for HIV treatment.

Additional data on the patients’ practices of mobilizing resources and the constraints and opportunities they encountered in the process were collected during participant observation at the ART clinics and in patients’ homes. Esther attended two of the three clinic days run by our treatment centres of focus every week for a period of six months. Whilst at the clinic she assisted in the delivery of various ART services, including counseling, registering clients and dispensing medicines. She also interacted with and participated in spontaneous conversations of patients waiting to be reviewed. Participating in activities at the treatment centre provided opportunity for lengthy interactions and informal conversations with both patients and health workers. This enabled us to triangulate the patients’ views on health- system related challenges they encountered. We were also able to observe interactions and exchanges among patients and health workers that pertained to accessing treatment and other necessary resources. In the homes, Esther participated in conversations of patients and their family members and observed interactions and exchanges between patients and family, friends, neighbours and other community members. Thus, through participant observation we were able to experience and observe some of the constraints patients encountered in accessing and adhering to treatment and how they were overcome at first hand [21]. All the interviews were audio recorded, whilst observations, discoveries and conversations made during home visits and participant observation at the treatment centres were recorded in field dairies on a daily basis.

### Data preparation and analysis

The interviews were transcribed, translated, word processed, compiled in to cases and imported into NVIvo.10 qualitative data analysis software package for coding. The software package aided cross- indexing of data that corresponded with more than one theme [22]. Data analysis for this article was conducted through an iterative process of inductive and deductive thematic analysis [23]. Initially, data on each respondent were read multiple times and the relevant sections, paragraphs and words coded according to two broad categories derived from the definition of the construct resilience. Resilience as a theme was drawn from the theoretical framework of the study–the strengths perspective. The two categories were: risk factor/adversity defined as events or problems that made it difficult for patients to access or utilize resources they identified to be important in the management of HIV like medicines, health facilities, food, information and support. Examples of these in the data were ill-health, poverty, fear of stigma, lack of information and stock-outs of medicines from the treatment centre. The second category was overcoming risk defined as successful access to and utilization of requisite resources in spite of the problems encountered. Examples of data coded under this category include incidents when patients accessed medicines when they were out of stock from their treatment centre, accessed HIV medicines without visiting the health facility in the fear of stigma, accessed the recommended diet of food amid poverty, ill health and without owning land. The coding was executed by matching the category with sections of data that reflected its definition.

A thorough scrutiny of data under the two categories revealed that besides using personal resources, several of the patients successfully overcame the problems to access and utilize necessary resources with the help of others. This suggested that social networks played an important role in the resilience of HIV patients. We tested the significance of this pattern by identifying and comparing the extent to which patients who defaulted on treatment due to adversity at one point in their lifetime, utilized their social networks to overcome the problems. It emerged that social networks were not as active in several of the latter cases. The incidents where patients succumbed to adversity were coded ‘non-resilient’ to ease retrieval for comparison.

From literature on social networks, we identified the theme social capital and applied it to sections, paragraphs and incidents where problems in access and utilization of resources were overcome with the support of others. Social capital was defined as any form of support from the patients’ relations. Data under the theme social capital were re-read and further segmented according to categories of bonding, bridging and linking networks to enable analysis of the role played by various relations. Bonding networks were defined as intimate relations with same socio-demographic characteristics as the patient such as family. Bridging networks were described as supportive individuals in the community whose relationship with the patient was not very intimate such as landlords. Linking networks were defined as influential relations based in institutions such as health workers. Themes for this article were derived from patterns identified from an analysis of the role of various networks in enabling HIV patients to remain on treatment when affected by specific barriers.

### Ethical considerations

Written informed consent was sought from the 50 patients that were selected to participate in the study. Patients who could not write used a thumb print. Additional verbal consent was sought from those who were selected for follow-up. Effort was made to protect the interests of patients who had accepted to be followed-up but did not want to disclose to significant others, including spouses. We always notified them about our intended visits in advance to obtain their clearance. For purposes of ensuring confidentiality, only pseudonyms are used in the article. The study was granted ethical clearance from Mengo Hospital Research Ethics Committee and Uganda National Council for Science and Technology.

## Analytic concepts

### Social capital and resilience

In this section, we give a brief description of our understanding of social capital as well as resilience.

Social capital is conceived differently by different scholars. However, there is consensus that social capital is rooted in social networks and is of instrumental utility to actors in those networks [2, 24, 25]. Drawing from a social network view of social capital [26] this article borrows Lin’s definition [2, 3, 27] of social capital as valued resources embedded in informal social networks. The resources include emotional (love, care, understanding), instrumental (money, labour, and food), appraisal (help with decision making) and informational (advice, information for care) support [28]. Other resources include power and influence and resourceful contacts. Like Lin [3] our definition considers network features such as trust, norms and values as contextual variables that impinge on access to social capital (embedded resources) but not integral to it.

Analytically, three forms of social capital can be accessed by individuals and groups in society, notably, bonding (from micro intimate relations often among homogenous groups such as families and friends); bridging (involving diverse groups of people such as work mates) and linking social capital–from vertical connections with influential people based in institutions such as health care facilities [10, 29, 30].

The term resilience is used to describe the attainment of positive outcomes by an individual or group despite exposure to significant adversity [31–33]. This implies overcoming the odds and continued positive functioning during difficulty, including poverty and illness. Therefore, resilience connotes adaptation to the difficult and bouncing back from distressful situations [32], be it financial, emotional or otherwise. Persons living on ART in resource-limited settings often face these constraints and need to surmount them to continue on medication. In this article, we analyze how social capital enables people living on ART in Uganda to overcome the risk of non-adherence due to selected structural and health system related barriers. Adherence in this article is viewed in the broad sense of meeting several requirements of the ART regime, notably taking the daily pill, routine check-up, prompt management of side-effects and taking the recommended food diet.

We focus on the process through which bonding, bridging and linking social capital protects patients on ART from risk but not necessarily measuring the outcomes [9].

## Results

### Study participants

Thirty one of the 50 patients were female whilst 19 were male. Ten of them were on only Septrin or its substitute Dapsone prophylaxis whilst the rest had started antiretroviral medicines. Their length of period on ART ranged from being newly initiated to nine years; whilst their ages ranged from 19 to 83 years. The majority (33) were married, five (5) single, four (4) widowed and eight (8) divorced/ separated. Eleven (11) of the married respondents had not disclosed to their spouse. In terms of occupation the majority (26) were subsistence farmers.

### Social capital and resilience among HIV patients

Analytically, social capital enables HIV patients to overcome the risk for non-adherence to ART when confronted with individual, structural and health system related barriers through bonding, bridging and linking networks. Bonding networks include close relations with the same demographic characteristics as the patient such as members of both the nuclear and extended family, friends, fellow patients, religious affiliates and neighbours. Bridging networks include community members of diverse characteristics whose relationship with the patient is not very intimate like workmates, transporters and landlords. Linking networks include influential individuals within institutions like health workers and religious leaders.

The focus is placed on the role of social networks in overcoming risk posed by five barriers; non-disclosure and fear of being stigmatized, inadequate information on diagnosis and management of side-effects, transport costs, medicine stock-outs and food insecurity.

The results are presented according to three themes that reflect how social capital works to enable HIV patients overcome risk: facilitating access to scarce resources; encouraging patients to continue on treatment; and averting risk for non-adherence.

Each section begins with a brief discussion of the risk posed by specific barrier (s). This is followed with presentation of data on the role played by patients’ networks in overcoming the identified risk. To further illustrate the important role of social networks in overcoming risk associated with particular barriers, examples of patients who did not seek assistance from their relations and defaulted on treatment are provided.

The reported results are mainly derived from semi-structured interviews and conversations with HIV patients, which are supplemented with data from our observations and conversations with health workers. Data from interviews, conversations and observations are presented in form of cases with direct quotations from the respondents.

### Facilitating access to scarce resources

Bonding, bridging and linking networks enable direct and indirect access to medicines, food and money, among other resources, when patients encounter barriers in obtaining them. The roles played by different relations are described under the themes: linkage to medicines amid stock-outs and fears of stigma; facilitating access to transport; and facilitating access to food as part of treatment as explained below.

#### Linkage to medicines amid stock-outs and fears of stigma

Whilst ART requires that clients strictly adhere to prescribed antiretroviral and prophylactic medicines and manage opportunistic infections (OIs) and other common illnesses promptly, stock-outs remain a common phenomenon in Ugandan health facilities. Health workers always advised patients to purchase unavailable medicines but several of them would fail due to poverty.

Faced with stock-outs several patients relied on health workers who used their position and sometimes connections to enable them access scarce medicines from other health facilities.

Hajji and his wife Faridah were friends with the in-charge of their local Health Centre III (HCIII). Although Hajji and Faridah faced financial constraints like other patients, most of the time they accessed the medicines they were advised to buy through the in-charge. “I go with the [treatment] books and ask him to check if the medicines prescribed are available at the health centre III. When they are there he gets them for us,” Hajji narrated.

Those without direct links to health workers accessed their help indirectly through relations connected to them.

Betina, a 45- year-old housewife, realized more sustainable stocks of Dapson, a rare and expensive substitute for Septrin, with the assistance of the wife to her landlord who worked as a nurse in a big public hospital. Betina was on Dapsone because she was allergic to Septrin. It was however perpetually out of stock from the treatment centre and she had to buy it most of the time. She had virtually bought the medicines during the six years of her treatment, with financial support from her husband and eldest son. The support of her husband and son had not completely removed the threat of running out of medicines for the next dose, because they were bought on a piece-meal basis due to the high cost involved. She indicated that sometimes they failed to raise enough money for her medicines. An opportunity to access Dapsone in ‘bulk’ emerged when her husband Bwire found out that the wife to the landlord- whom he helped to collect ground rent from fellow tenants- was a nurse in a big public hospital. Bwire seized the opportunity to inform the landlord about the problems of accessing Betina’s medicine and requested him to engage his wife on possibly getting for them dapsone from her hospital. A couple of months later Bwire informed us that accessing Dapsone was no longer a major problem because the wife to the landlord often sent them a tin or two of Dapsone which covered Betina for months.

For accountability purposes, public health facilities in Uganda discourage dispensation of medicines they have not prescribed. Hajji and Betina nonetheless managed to obtain medicines prescribed from elsewhere because health workers directly and indirectly linked to them, respectively, used their influence to enable them access these institutional resources.

The utility of networks of health workers as links to medicines amid stock-outs was further demonstrated during a short-lived shortage of some antiretroviral regimens from Mukono HCIV during the study. Clients whose regimens were missing were advised to buy or ask from a nearby mission facility or other treatment centres and return for refill after two weeks. Our follow-up interviews with these patients showed that majority who succeeded in accessing medicines from the mission and other treatment centres knew someone who worked there or had a contact who knew someone there. Several of the contacts were relatives, friends and health workers in the ART clinic of Mukono HCIV. An expert patient at Mukono HCIV referred patients who sought his assistance to a fellow expert patient in the mission facility. He called and explained the situation to his colleague beforehand and then told the patients to mention that he was the one who directed them there. Susan accessed the medicines through a sister whose friend worked as a nurse in another public health facility. She said that she explained the situation to her sister who then sought the assistance of her friend.

The patients who were unable to mobilize money to buy the medicines or contacts to link them to alternative sources defaulted on treatment. A middle-aged woman shared that she survived on the extra pills they are given for the next three days and remained without medicines for the rest of the period.

Besides stock-outs access to medicines is constrained by patients’ fears of being stigmatized. Even with increased access to HIV treatment stigma remains a concern in Uganda and affects the use of ART services. Patients on treatment are required to return to the clinics on a routine basis to pick new supplies of medicines (refill) and to monitor their progress. However, patients who do not disclose to spouses, employers and other significant relations in the fear of being stigmatized, commonly keep away from the treatment centres to avoid being exposed. The infrastructure at the treatment centres several times does not allow total confidentiality. Some ART clinics are conducted in an open space, and because they exclusively attend to HIV patients one’s presence is an indirect declaration of a positive HIV status. Whilst avoiding the treatment centre usually helps patients who prefer anonymity to prevent potential gossip and rumours about their status, it exposes them to the risk of defaulting on or stopping HIV treatment. Sometimes patients who have not disclosed to employers do not deliberately avoid coming to the treatment centre but may find it difficult to attend routine ART clinics due to lack of time and space. Attending routine clinics requires substantial amounts of time away from work. In the two treatment centres of focus, patients take an average of five hours to complete the whole cycle of treatment from payment of user fees, triage (registration and taking of basic measurements such as weight, height, blood pressure, nutrition status), monitoring tests (CD4+ count, viral load, full blood count) clinical review by the doctor and receiving new supplies of medicines. It may thus not be possible to keep away from work for that long on a regular basis without arousing suspicion from the boss and fellow employees if any.

Some of these patients seek linkages with health workers and rely on the help of fellow patients to continue accessing HIV medicines and treatment services without attending routine clinics or arousing the suspicions of employers. The health workers use their positions to help the patients access services outside the normal operating procedures of the treatment centre. This practice was more conspicuous in the public health facility where supervision and management were weak.

Ronald managed to access HIV medicines and diagnostic services, with the help of a counselor, despite not attending routine clinics during his four years on treatment in the fear of being exposed. He usually stopped at the main entrance of the health facility and called this counselor to pick the book with his treatment details from him. The counselor often updated his file, collected his medicines from the dispenser and linked up with Ronald later in the evening to hand them over. Ronald only utilized the services of the treatment centre when his CD4+ count and viral load tests were due. Still in these cases he did not reach the ART clinic, but stopped at the entrance of the health centre, asked ‘his’ counselor to take him a filled laboratory request form and then went straight to the laboratory which serves all patients of the health centre. He left immediately after the tests and relied on the counselor to pick his medicines.

Peace also relied on the help of a counselor to collect her medicines. Unlike Ronald, she only sought the counselor’s help when she failed to find an opportunity to sneak away from work. She said that she worked as a secretary for a very strict boss who did not allow any of the workers to leave, except when they had an emergency. She could not ask for special consideration because she had not disclosed her HIV status to them. Her only option was to sneak out, but sometimes she failed.

“I do the work assigned to me very fast to create time for coming to the treatment centre. When I come, my counselor Badru helps me to get through the process quickly so that I run back to work before my boss complains. But many times, the boss gives me one assignment after the other and keeps monitoring my progress. It becomes impossible to leave. In such cases I call counselor Badru and ask him to collect the medicines for me, then I pick them from him after work.”

ART clients usually become close to counselor (s) who initiate(s) them on treatment. Most of them described these counselors as their friends. News about a positive HIV status is certainly distressing. Thus, the position of counselors as comforters and scepters of hope naturally endears them to their clients. The initial counseling encounter in many cases marks the onset of an enduring friendship that patients like Peace and Ronald usually draw upon to access resources from the treatment centres when faced with adversity. Most patients usually nurture their relationships with counselors and other health workers with gifts in the form of food and sometimes money. Similar gifts are given to appreciate and arguably sustain the help extended by the health workers.

Health workers who are also community and clan members are several times compelled to use their positions to assist close relations, such as kin, out of moral obligation. It is common for health workers to carry medicines for patients in their communities or relatives without them visiting the health facilities. One nurse used her position to enroll and support her uncle Frank to remain on HIV treatment without him visiting the treatment centre. Frank had confided in her about his HIV positive status but refused to enroll for treatment even after she advised him to do so. A respected opinion leader and elder in the area, Frank expressed concern that people would question his morals if they saw him at a treatment centre and concluded that ‘a man of his age and social position had HIV’. The nurse felt moral pressure to have her uncle enrolled on HIV treatment. She feared that people would blame her if Frank died. “I have no option but to intervene”, she explained. “He is like a father to me. If he dies, how will I explain that I could not help?” She took Frank’s blood sample from home and brought it to the health facility for examination of his CD4+ count. When the results showed that he was eligible to start antiretroviral medicines, she bought a book and prescribed him a single dose regimen which was at the time preserved for pregnant and lactating mothers. Asked why she was doing this, she told Esther that her uncle was poor at taking medicine so she could not risk putting him on regimens with a high pill burden. She always picked his medicines and brought his blood samples to the treatment centre for examination every three and six months, respectively, as required. Her assistance enabled Frank to overcome the risk of not initiating on and later potentially dropping out of treatment due to his fears of stigma.

Besides health workers, patients who find it difficult to visit treatment centres due to non-disclosure may depend on the help of fellow patients to access medicines. The treatment centre is a place and space for HIV patients to meet, interact, share experiences and exchange ideas about managing HIV. Bonds of friendship and trust mediated by shared interests and experiences are sometimes borne out of these interactions and facilitate the formation of alliances geared at addressing common challenges in treatment seeking. These alliances operate on principles of trust, cooperation and reciprocity, and normally bind the members to either pool resources towards a common cause or share responsibility for executing a practical task. Namatovu depended on such mutual support arrangements to reduce waiting time and beat the restrictions of her employers that made it difficult for her to attend routine ART clinics. Namatovu taught in a private secondary school and had not disclosed her status to the school administration in fear of possible discrimination. The school required all teachers to be present during working hours, unless one had a good reason backed by evidence to be excused. Namatovu sneaked out from work on her clinic days in the hope that she would get a quick service and return before the head teacher noticed her absence. She was however always let down by the long waiting time. She said that sometimes she waited for as many as seven hours. One day, as she waited for her turn with several other patients one of them suggested how they could support each other to reduce the waiting time.

“She proposed that we exchange registration and personal telephone numbers such that whoever arrived first registered all the rest. The rest of us would call each other in advance to confirm that we were scheduled on the same day. Six of us liked the idea. We exchanged our names, registration and mobile telephone numbers.”

This arrangement helped Namatovu to reduce waiting time significantly. She was able to pick her medicines without getting in trouble with the school administrators. We observed that she usually arrived at the clinic after 10:00am but her name was always among the first 20 on the registration list of over 100 patients.

It was common for patients who did not exploit relations with health workers and fellow patients in this way to succumb to their fears of being stigmatized and default on treatment. Brian told of how he abandoned HIV treatment for two years on realizing that it would be difficult to keep his status discreet from his girlfriend if he continued attending ART clinics. He had a live-in girlfriend but was not ready to disclose to her. However, the first ART clinic he attended gave him the impression that he was at the risk of being exposed. He said that he had seen a couple of people he knew waiting for the same service, and after serious reflection decided to stop visiting the treatment centre. “I said I am still on Septrin which I can buy. I don’t need to go back there to expose myself.”

#### Facilitating access to transport

Adhering to ART requires regular travel to the treatment centre for refill and routine check-up.

The main challenge with transport is the cost. Lack of money for transport is a common reason for patients to skip treatment appointments. Whilst HIV treatment services in Uganda have been further decentralized to lower level health facilities such as HC III [34], many patients stay long distances away from the nearest facility, yet others prefer to seek care from distant treatment centres as a way of avoiding stigma.

Most of our interlocutors who overcame difficulties in mobilizing transport resources mainly drew on support from bonding networks of family.

Parents, children and siblings were the primary sources of monetary and practical support towards transport. Patients who had disclosed usually contacted family members for money or their means of transport. However, sometimes the support was volunteered by concerned individuals in the family. Eighty-three-year-old Matiya always asked one of his sons to drive him and his young children on ART to the treatment centre, because he found it very expensive to transport him and the children by *bodaboda* (motorcycle taxi) from his remote village. His son would drive over 70 kilometres from the city the night before and sleep in the village to be able to drop them at hospital by 6:00am the following day. On the other hand, Rose was frequently ill but did not want to ‘bother’ any of her relatives for assistance with money for transport and other treatment needs. However, her mother provided her support. She narrated that her mother had got concerned about her situation and decided to take over her treatment costs.

“She came to visit and found me in a terrible shape. I was thin and basically struggling to survive. When I shared my challenges with getting money for transport to come there [treatment centre], food and buying medicines she got concerned and told me to inform her when I need anything… When you ask her she provides. Like, when I need money for transport to come there I inform her in advance. She always gives me. “

Like Rose, Liz was always offered support to meet transport charges by concerned family members. She related that her parents and siblings asked her to always inform them about her dates for visiting the clinic, and whenever she does they voluntarily contribute money towards her transport, user fees and any other treatment bills she identifies.

Patients who had not disclosed to family usually devised subtle means of obtaining monetary support towards their transport from them. Some disguised by giving generic reasons for seeking financial help. Asked how she managed to obtain money for transport from close family when she has not disclosed, a 29-year-old woman said, “I just tell them that I have a problem. Please send me 10,000 or 20,000 shillings, and you manage like that.” Other patients feign different illness whilst those with known chronic conditions such as ulcers and eye complications may advance their treatment as the reasons for seeking financial help to travel to the health facility. To increase the odds for success, some clients sought to borrow rather than ask for direct financial assistance from family. Matana observed that her paternal aunt always gave excuses when she asked her for direct monetary support towards her transport, but was quick to respond when she placed her request as a loan she would clear in a given period of time.

It is evident that several kin still take their traditional obligation of caring and supporting needy relatives as binding; even though it is widely acknowledged that the protective capacity of the family and kinship support system in Uganda and other sub- Saharan African countries has been weakened by the economic and social pressures of modern times [35]. This is not to suggest that all patients have functional family support systems. On the contrary, several of our interlocutors were struggling to meet transport and other treatment needs due to limited family support. These patients overcame difficulties in mobilizing transport by drawing on the support of bonding and bridging networks outside the family like friends, fellow patients and transporters among others. These diverse networks appeared to provide assistance out of compassion for and/or as an expression of solidarity with constrained patients.

Transporters assisted constrained patients by providing them transport services to the treatment centre on credit and sometimes for free. Tinka, a migrant from south western Uganda was partially blind-due to an untreated cataract and living very far from his relatives but always managed to travel to the treatment centre with the help of transporters. Whenever Tinka failed to mobilize money for transport he shared his problem with the *bodaboda* riders he often hired to travel to the treatment centre.

“When I have no money, I explain to bodaboda riders known to me and request them to bring me [to the treatment centre]. It is impossible not to find at least one who sympathises with you and transports you for free or on credit.”

Alisaba who was conspicuously thin and frail was often exempted from paying for the services of *bodaboda* riders in her village, which she said enabled her to accumulate savings that she drew upon when the brother she depended on for financial support was constrained. She told of an incident when one of the cyclists rode her to the highway and when she reached her hand bag to pay him; he told her to leave the money and instead wished her a safe journey to the health facility. Such gestures appeared to be the transporters’ way of expressing solidarity with an ailing member of their community.

A few health workers were involved in supporting needy patients to meet transport costs. These health workers typically drew from personal resources and therefore acted more like a bridging network of concerned and compassionate community members. Twenty-three-year-old Mutetsi, got regular monetary support to meet transport needs from a nurse she had met in the process of seeking care for her ill daughter. Mutetsi was a housewife, but her husband was not supportive. She initiated the supportive relationship with the nurse when she (nurse) expressed concern about the poor health of her baby, and personally helped them to access testing and treatment services from the laboratory and the ART clinic. She premised on the nurse’s concern to share her social and economic struggles with her. The nurse sympathized with Mutetsi and hence forth gave her money for transport when she called to inform her that she had failed to mobilize enough money.

Other patients who drew on their networks to overcome challenges in mobilizing transport were in mutually supportive treatment alliances -with fellow patients, focused on sharing the monetary costs associated with transport. An example is a group of three women who had an arrangement of travelling in pairs, when possible, to reduce the transport costs incurred by either of them. The three women lived in a remote village located about 60 kilometres from the treatment centre and did not know they were living with HIV until they bumped in to each other on clinic days. The idea of sharing transport costs was initiated by our interlocutor Betina who observed that they were wearing her down at the time.

“We would check with each other to confirm if any two of us were scheduled to return for refill on the same day. When the days coincided we booked one bodaboda and negotiated for a discount. One person is charged 10,000/ = (USD 2.7) but when we were two we negotiated and paid a total of 15,000/ = (USD 4.3).”

Betina observed that the arrangement enabled them to reduce the costs incurred by either of them to manageable levels, and without it they would not have managed to finance the routine visits to Naggalama Hospital.

#### Facilitating access to food as part of treatment

Nutrition is a key component of the ART regime. In health education talks, HIV patients are advised to eat an adequate and balanced diet to improve the efficacy of the treatment. ‘Eat well’ is common advice given during HIV counseling sessions. Patients are also advised to take their medicines after a meal to minimize the occurrence of some side-effects. Access to an adequate quantity and quality of food is nevertheless complicated by food insecurity. With the increasingly limited land for cultivation, climatic changes and other agricultural problems in the country food insecurity is a problem to both patients in rural and urban areas, but is probably worse for the urban poor.

Most of our interlocutors who managed to comply with the dietary requirements of HIV treatment despite being vulnerable to food insecurity benefitted from the support of a diverse network including bonding, bridging and linking relations. Some of these relations facilitated access to food directly through provision of food in kind and contributing financial support towards the patients’ food budget. Eve shared that in addition to her mother and brothers calling to remind her to take her medicines, they also advise her on what to eat and provide money to buy it. Cathy lived in town, but told us that ever since her mother learnt that she was HIV positive and on medicine, she sends her food from the village on a weekly basis.

Besides family, community members are significant supporters in accessing food. These mainly support through provision of land for cultivation to the needy. Kagoya, her husband and four children accessed adequate food through cultivating land that was lent to them by a compassionate landowner. Kagoya and her family had migrated from Busoga in eastern Uganda and rented a small room. They were landless, did not have relatives in the area and visibly living in squalor, with difficulties in accessing basics and food in particular. Seeing how Kagoya was suffering with her family, a renowned affluent man in their village offered them free land to cultivate. Kagoya’s husband, a cobbler, got the offer when he had taken back the repaired shoes of the landowner and asked for a small piece of land for his family to cultivate. The landowner told him to use part of his vast land to grow food if he liked. He was probably moved by their situation and decided to help in the best way he thought he could. “My husband just asked the old man for some little land to dig but he offered us a much bigger piece, and wanted to add us more, but we declined the offer because we could not use it,” Kagoya explained. Kagoya and her family got additional support from a local Pentecostal church they prayed from when one member offered them a daily supply of a litre of milk from her farm. Kagoya termed the offer as the lady’s ‘investment’ to receive God’s blessings.

“This sister [in faith] just got touched by God to give us the offer. We have been getting it for about two years now. Actually, people here always wonder where we get money to buy milk on a daily basis.”

The practice of regularly giving -usually material things- to needy persons as an investment in further blessings is locally known as *okusiga* and is encouraged in many religions as an enactment of faith.

Maria a 41-year-old widow utilized land offered by the lead priest of her local catholic church to generate adequate food for her and the six children. A victim of land fragmentation, Maria had no land to cultivate. Her deceased husband had left her with a very small piece of land inherited from his father, which was just enough to accommodate their house, kitchen and latrine. She was an active member of the church and known to almost all the clergy. When she shared her challenge with the lead priest and requested to borrow a piece from the vast church land in her neighbourhood, he gave her permission to choose a convenient portion to cultivate.

As shown, bonding networks like family, friends and religious affiliates, bridging networks like affluent community members and linking networks of religious leaders may cushion HIV patients against risk associated with food insecurity by facilitating their direct and indirect access to food.

### Encouraging HIV patients to continue on treatment

Networks of fellow patients and health workers are important sources of information that encourages patients who feel compelled to abandon the medicines due to extreme side-effects to continue on treatment. Adherence to ART is often constrained by patients’ lack of adequate information on the symptoms and management of side-effects of antiretroviral medicines. Patients starting treatment are always oriented about ART, side-effects of antiretroviral medicines and their management to empower them to take appropriate decisions in case they react. We nevertheless found that the orientation sessions are usually not enough to prepare patients for what is to come. The side-effects are sometimes not only ‘peculiar’ but also so severe that patients and their care takers are not able to explain what is happening to them. Such confusion commonly compels patients to discontinue medication when it is uncalled for. Overcoming these information barriers requires that HIV patients access prompt and timely information on diagnosis and management of side-effects from experts on ART.

Health workers and fellow patients were the main sources of information, expert advice and encouragement that enabled patients to overcome confusions that were threatening their continuation on treatment.

As members of informal networks in families or the community, health workers provided professional advice outside the formal health worker-client relationship. Patients usually sought out health workers they knew in the community or called their personal phone numbers for advice. Counselors and often volunteer expert-clients commonly share their personal phone numbers with patients voluntarily or on request, and further permit them to call anytime in case of a problem related to HIV treatment at their own initiative. Clients are thus able to access timely information and expert advice to eliminate notably disruptive confusions and distress [12] to continue treatment.

Tolo narrated how the advice of an expert-client enabled her to get a quick solution to a baffling side-effect that threatened her continuation on medication. She had just started antiretroviral medicines then her stomach started swelling. She and her mother were confused about it and contemplated discontinuing the medicines. They consulted an expert-client who lived in their neighbourhood. Tolo knew him because he had once mobilized PLHIV to contribute money for self-help projects that did not take off. Tolo and her mother strolled to his home one evening and narrated her situation to him. He looked sternly at her for a while and then said that he knew what the problem was. “He said that I was swallowing medicine without drinking enough water and that antiretrovirals are supposed to be accompanied with a lot of water. He advised me to drink a full cup of water after taking the medicine.” Tolo followed his advice and within a week became better and continued with her medication.

Similarly, Joshua a client of Naggalama Hospital narrated how the advice of a counselor encouraged him to abandon the idea of discontinuing medication when he experienced severe vomiting.

“I vomited and vomited for one, two, three weeks. I would vomit anything I ate. I reached a point where I had got fed-up with the medicine. I felt a strong urge to throw it away. I was fed-up. I called the counselor, explained my condition to him and inquired if it was okay for me to get off the drugs, at least for a while. The counselor said that getting off the drugs was not a good idea. He told me to continue taking the medicine, but go to the nearest health facility to manage the vomiting. My friend, he encouraged me to persevere and after sometime I got better.”

Fellow patients mainly offered each other advice derived from personal experiences with ART.

Micheal told of how a talk with a fellow patient gave him the confidence that the frequent headaches he was having were a common side-effect that did not warrant discontinuing antiretroviral medicines. He said that he suffered a severe headache a week after starting HIV treatment and at first did not understand what was happening to him and contemplated giving himself a break from the medicines. He nevertheless decided to first consult a fellow patient he knew had been on HIV medicine for a while to confirm if he had got a similar experience.

“He asked me if the tablets I was swallowing were blue, then I said yes. He told me that he too had got headache and that headache is common among people on this particular medicine. He advised me to drink a lot of water and to buy hedex [a strong pain killer]. I followed his advice and got better over time.”

Micheal shared that he had encouraged several newly initiated clients- who confided in him that they had plans to abandon the medicines due to severe side-effects, to carry on. He indicated that he always draws on his experience to convince them that the body eventually tolerates the medicine.

It was not uncommon that patients who abandoned the medicines when they experienced side-effects they considered severe had not consulted health workers or fellow patients.

On a sunny Tuesday morning, Mzee walked straight to the dispensing window of the ART clinic of Mukono HCIV and dumped there a black polythene bag containing his newly initiated antiretroviral medicines.

“Here is your medicine, he said. I do not want it anymore. I have taken alcohol all of my life, but I have never felt this drunk. How can one sleep and wake up drunk for a whole week? Can you imagine I stopped taking this medicine a week ago but I still feel dizzy? Take it I am no longer interested. “

It took extra counseling and reassurance for Mzee to be convinced that he had to persist to benefit from the medicine, and that his body would eventually tolerate it. Mzee’s experience further attests to the important role played by informal networks of fellow patients and health workers in encouraging adherence among patients experiencing information barriers.

### Averting risk for non-adherence

Social relations provide the practical support that enables patients to avoid situations that can cause them to skip doses when significant others they have not disclosed their HIV status to are around. The site for keeping HIV medicines can complicate their use by patients who fear to disclose to close relations such as spouses. Several of these patients hide their antiretroviral medicines to avoid arousing suspicions that may culminate in potential rejection. Such secrecy presents risk of skipping doses when the individuals they do not want to know are around.

Bonding networks of trusted friends, relatives and neighbours were instrumental in assisting patients to avert such risk. They helped to keep the medicines away from the patients’ homes, thus enabling them to continue on treatment undetected. Keeping the medicines away further minimized the chances of skipping doses when significant others, like spouses, who had not been disclosed to were around. Stella asked a neighbour and friend to keep her HIV medicines when swallowing them on time was complicated by her husband’s almost constant presence at the time she was supposed to take her dose. She met and married her husband after several years on HIV treatment but decided not to disclose to him in fear of possible rejection, and consequently hid her medicines from him. Stella took her tablets once at 8:00pm, a time that was convenient before she got married. This became difficult when she started living with her husband because he was almost always around.

“He normally returns from work by 7:00pm and watches television. We rent only one room. It was difficult for me to retrieve the medicines from under the bed, where I hid them, on a daily basis [without arousing his suspicion] … But now, when it is time I just walk to my friend’s house. It is a few houses away. “

John a *bodaboda* rider kept and took his medicines from the home of a workmate and friend to prevent his wife from seeing them. John was convinced that his wife would leave him when she found out that he was HIV positive, because she regularly cautioned him against promiscuity.

“That woman is tough. I know she will leave if she finds out. I know it. I cannot let her suspect anything, so I keep my medicine with a workmate who is my friend. I pass by his home and take my morning pill on my way to work and take the evening one on my way home from work.”

Sarah a 23-year-old house wife kept and took her medicines from the home of a sister whom everybody in the family, including her husband, knew was HIV positive. Sarah was on TDF/3TC/EFV a single dose regimen she was advised to take at night. She went to her sister’s home to take the medicine every evening. When her husband was around she found an excuse. “I can for instance tell him that I need to pick something from my sister, or my sister is not well and needs my help.” Sarah had learnt her status during a routine antenatal check-up and feared to inform her husband. When she confided in this sister, she advised her to keep the medicines with her (sister) because she could easily cover her up in case her husband suspected anything. This was the case when Sarah’s husband returned abruptly from work and found her preparing to take the new supplies of antiretrovirals she had picked from the ART clinic to her sister. When he inquired where she had got medicines that looked like for HIV patients she told him that they were for her sister, and that her sister had sent her to pick them from the HIV clinic on her behalf because she was not feeling well. Her sister covered her up by confirming the narrative when Sarah’s husband called to verify his wife’s story.

Problems in adherence were common among several of our interlocutors who had not disclosed to close people such as spouses but did not solicit the support of their networks to keep the medicines away from home. In fact some felt that the involvement of family and friends among other relations would increase the risk of exposing their status to those they did not want to know. Allen who was in a discordant relationship but had not disclosed to her husband, related that she was sometimes forced to skip doses when her husband returned from work earlier than expected.

“I hide my medicine in the cupboard which I know my husband never checks…I take the morning pill at 8:00am and the evening one at 8:00pm. He is usually at work at those times. He loves watching films. Sometimes he returns earlier and watches films in the living room, where the cupboard is, for hours. It becomes difficult for me to get the medicines. I am usually forced to skip the dose to avoid creating trouble for myself.”

In spite of having friends in the neighbourhood, we observed that Allen could not keep her medicines with them because she did not trust them enough to disclose her HIV status. She noted that the only person she trusted was her mother, but she lived over 200 kilometres away.

## Discussion

We have presented how social capital in its various forms facilitates resilience (sustained adherence) among HIV patients on ART in the face of the barriers of stigma, medicine stock-outs, food insecurity, difficulties in mobilizing routine transport and inadequate information on diagnosis and management of side-effects. The aim was to provide insights in to the protective mechanisms of social capital so as to suggest ideas on how it can be harnessed to promote resilience among HIV patients on ART in a resource-constrained setting.

We show that in as much as HIV patients encounter several barriers to ART adherence, they do not necessarily succumb to risk. Rather, several of them draw on the resources of their social networks to overcome the risk for non-adherence posed by the numerous barriers to continue on treatment. This highlights the importance of social capital in promoting resilience among HIV patients in resource-constrained settings, a finding that is consistent with those of Ware and colleagues [4]. Since social capital plays a prominent role in promoting resilience among HIV patients in resource-poor settings, arrangements to expand and sustain it may be a necessary component of comprehensive ART programmes in these areas.

We found three mechanisms through which social capital facilitates resilience among HIV patients on ART, that is, by: facilitating access to scarce resources; encouraging HIV patients to continue on treatment; and averting risk for non-adherence. These mechanisms lend themselves to findings in several studies of sub-Saharan Africa [4–6, 15]. The mechanisms also relate to the protective processes identified in resilience literature. It is indicated that protective factors work to overcome risk through three mechanisms. They have a compensatory effect when they directly reduce a problem/adversity [36], a mediating effect when they modify the effect of adversity in a positive direction and a moderating or buffering effect when they reduce the negative effects of adversity [32]. In this case, social capital operates through mediating and moderating processes. The mediating effect is seen when advice from fellow patients and health workers encourages patients to continue on medication despite experiencing severe side-effects. The facilitation of access to scarce resources and aversion of risk both have a buffering effect as they reduce the likelihood that patients will default on treatment requirements when faced with barriers. The buffering effect is documented to be the dominant protective mechanism in other contexts of high risk [32].

Bonding, bridging and linking networks all play a prominent role in promoting the resilience of HIV patients on ART. As analytic constructs bonding, bridging and linking networks tend to be described as mutually exclusive [10, 29, 30], but in practice they overlap. For example health workers as a category can be simultaneously family and community members and thus sources of bonding, bridging and linking social capital. They serve as links when they use their positions to leverage scarce resources for patients who do not share very intimate bonds with them, but are more of bridges when they are touched to use their personal resources to support needy patients and simultaneously as bonds and links when they help close relatives and friends to access resources from the health facility.

Bonding networks of family are however the primary source of support for overcoming financial and structural barriers to adherence. These observations are consistent with the findings of several studies from sub-Saharan Africa [4, 5, 7, 15]. This article nevertheless underlines the significance of networks beyond the family in facilitating resilience where the family support network is weak. These include bonding networks like friends, fellow patients, neighbours and religious affiliates, bridging networks like transporters and affluent community members and linking networks like health workers and religious leaders. The results show that these non-family networks compensate for weaknesses in the immediate support network of HIV patients, ensuring their continued protection against risk in the absence of a functional state welfare system. This suggests that possession of diverse networks of social relations improves HIV patients’ opportunities for resilience in a resource constrained setting. Promoting sustained adherence in resource-poor settings would therefore necessitate that HIV patients on ART are encouraged and supported to diversify their social networks. This calls for proactive initiatives such as increasing opportunities to network through participation in mutual support groups at health facility and community levels; equipping patients with skills to initiate and sustain relationships; and intensifying the fight against HIV-related stigma to increase acceptance and integration of HIV patients in broader community networks.

Access to resources of various bonding, bridging and linking networks of HIV patients is mediated by social and religious values and norms like reciprocity, trust, cooperation, solidarity, concern, compassion and moral obligations to share and care for others as demonstrated in the results. These network features correlate with what is termed as collectivist norms [37]. Collectivist norms are associated with collectivism, a cultural orientation that subordinates individual for group interests. Collectivistic societies are concerned with the welfare of the group and obligate and commonly exert pressure on individual group members to serve the interests of others [38, 39]. Accordingly, values such as family cohesion, cooperation, solidarity, sharing of material and non-material resources, empathy, conformity with group norms and standards and consciousness about the needs, goals and aspirations of others and working towards their fulfillment are essential features of collectivistic societies [37, 39, 40]. As shown HIV patients on ART in Uganda ride on these collectivist values to access the embedded resources that enable them to overcome risk posed by poverty, medicine stock-outs, information barriers and stigma. This suggests that a value system that promotes collectivist norms is necessary for the realization of the protective benefits of social capital. As Binagwaho and Ratnayake [8] suggest, harnessing the protective benefits of social capital in resource-constrained settings necessitates proactive measures to invigorate and maintain collectivist norms in contexts that are increasingly tending towards individualism.

Despite its protective benefits, social capital is not devoid of limitations. Besides the inability to provide support due to poverty, we have shown that it is sometimes difficult to utilize available network resources due to fears of stigma. The imperiling effects of stigma on the utilization of social support were also reported in South Africa. Gilbert and Walker [41] found that several PLHIV avoided disclosing in the fear of being stigmatized and discriminated and in the process had to forego support provided by both informal networks and the State. In addition, whilst reliance on bonding and linking networks may help patients to minimize non-adherence due to stigma, the protective process inadvertently reinforces the practice of non-disclosure, particularly to intimate partners. This practice seems to be aided by the fact that most patients on ART attain relatively high levels of physical health. They are therefore able to obtain support for HIV treatment from significant others indirectly, for instance through using lies, as we have shown in the results. Non-disclosure may limit the accumulation and utilization of social capital [42]. It may further increase risk for infection and re-infection of intimate partners- where viral suppression is not attained, and is therefore counterproductive to public health. These observations highlight the potential role of economic empowerment programmes and initiatives to reduce stigma at community level in enhancing the protective benefits of social capital.

A limitation of our study is that it does not sufficiently address the individual patient attributes that interact with social capital to facilitate its protective benefits. Resilience is an outcome of a dynamic interaction of individual and environmental factors [32]. Thus interventions aimed at enhancing the protective benefits of social capital would benefit from understanding the dynamic processes involved. Future studies can contribute to this understanding by examining the individual attributes of HIV patients whose sustained adherence is associated with social capital.

## Conclusion

Social capital is a key resource that can be harnessed to promote resilience among patients on ART in a resource-poor setting. Invigoration and maintenance of collectivist norms may however be necessary if its protective benefits are to be fully realized.

## Supporting information

S1 Field Notes Field notes. (DOC)

S1 File Interview transcript Client 001. (DOCX)

S2 File Interview transcript Client 002. (DOCX)

S3 File Interview transcript Client 003. (DOCX)

S4 File Interview transcript Client 005. (DOC)

S5 File Interview transcript Client 006. (DOCX)

S6 File Interview transcript Client 007. (DOC)

S7 File Interview transcript Client 009. (DOC)

S8 File Interview transcript Client 011. (DOCX)
