# Supplementary material for: Social capital and resilience among people living on antiretroviral therapy in resource-poor Uganda
Source: PLoS One. 2018 Jun 11;13(6):e0197979. doi: 10.1371/journal.pone.0197979 (PMC5995438; doi:10.1371/journal.pone.0197979)
Supplement: S1 Field Notes — (DOC) [file pone.0197979.s001.doc]

**24^th^ August 2015**

I had plans to commence fieldwork at the beginning of September. I decided to seek clearance from the district health office and also the administrative departments of the 2 health facilities. The district health officer (DHO) didn’t complicate anything for me. He quickly stamped my papers from the university. I then decided to proceed to one of the facilities. There I asked for the in-charge. He was out of office. The nurse I talked to told me to wait on the bench outside the theatre building and explained that he often used the office attached to the theatre rather than the official one. I sat and waited patiently. While I was there two young women came and sat next to me. They asked me if I was waiting for the in-charge. I told them that I was and asked if they knew him. They said they did, and would alert me when he came. They too were looking for him. They were from a local NGO which targeted teenagers who had dropped out of school but were interested in learning any vocational skills. They knew that several girls get pregnant and come to the health facility, so they wanted to ask the in-charge to notify other staff to refer such cases to them. We sat for over an hour and had started talking about general issues when he came. The two girls knew him and alerted me when they saw him. But he didn’t go to office. He walked straight in to the theatre. The older of the girls walked up to him and told him we were waiting to see him. He told her to wait, he would come shortly. A few minutes later he came out asking for the care taker of a mother who was in theatre. He wanted her to take the newborn baby away from the bed in the resting room. He had a patient to examine from it. We could hear the baby crying. There seemed to be no one claiming her. He asked one of the nurses to go to the maternity ward and look for the care taker. She returned with a middle aged woman, who picked the baby and started pacing around with it to calm it down. Minutes later, the mother who seemed still unconscious was pushed to the ward on a stretcher. They kept calling the caretaker who was carrying the baby to push the patient as well. I complained to the nurse who had brought the mother out of the theatre. She told me there were no people to push patients; one had to come with energetic people to push the stretcher up hill to the ward. After about 30 minutes he came. We agreed that they should see him first since my issues were likely to take a longer time to sort. When they came out after about 5 minutes, I got in. The room was small and seemed like an entrance to the theatre. Several white boots lay on the floor and white coats hung on hooks fixed on one side of the wall. The room had only two chairs and one table. The table was empty, and didn’t seem official office to me. He sat on the chair across the table, while I sat on the opposite side. I introduced myself, showed him the papers that the DHO had endorsed and explained that I needed clearance to start participating in the activities of the HIV clinic and to talk to a few clients. He noted that there was no problem and handed me back my papers. He got up and told me to follow him. As we walked out a young woman came. He introduced her as one of the ‘*basawo*’ (health workers) who work in the HIV clinic. I greeted her and introduced myself. He explained that I would be talking to people living with HIV (PLHIV) for my research. She said I was welcome. The two of them started talking as I stood by. She complained that they hadn’t received any money as they had been promised. She had plans to go back to the chief administrative officer’s (CAO) office and complain. He encouraged her to do so and said he had pushed and reached his limit. She noted that they were planning to go on strike the following week. The in charge told her they should take whatever step they thought would help them get their salary faster and promised to see someone over the threat of a sit down strike. Another nurse looking for the in-charge came. We left the two of them talking then we proceeded to the ART clinic. It was not a clinic day, but the in-charge of the unit was there. She was working on a number of files. I was introduced to her, and her boss asked her to render me all the necessary support. He left me there. As he left I noticed that he had given me back all my papers and so didn’t have any record for filing. I informed him. He didn’t seem to think it was necessary for him to take any of the papers, but I insisted until he took them. I talked to the in-charge of the unit. I asked about clinic days and how I could get involved in the activities of the clinic. She said they had 3 clinic days, Tuesday, Wednesday and Friday. They didn’t work on Monday, but Thursdays were days for handling data. She told me she was going on maternity leave the following day but I should come on any of the clinic days and talk to whomever I will find. They would help me. I asked for her phone number which she gave me. I promised to keep in touch and to start the following week. I then left and drove to the other facility. I had not been to this place before, but it was not difficult to find. A guard in a green uniform opened for me and directed me where to park. I could already sense the difference between the two health facilities. After parking I was asked to register in the book, which I did. I then inquired where the medical superitendant’s office was. He directed me. The building was in sight, so I walked there. I got to the reception and found two desks. One was occupied by a young woman while the other was empty, albeit there were signs that someone was seated there but had stepped out. There were 3 chairs for visitors to sit on. The woman I found was busy working on her computer. I greeted her and asked to see the superitendant. She told me he was away but I should wait for his secretary who had stepped out a bit. She told me to sit on one of the chairs and wait. I did. She handed me newspapers to read. 30 minutes later the secretary came. I was introduced to her. I explained why I needed to see the superitendant. She gave me a visitors’ book to write in and said she was going to call him. He was around but probably in the wards. She used her mobile phone and after a while got him. She explained that he had a visitor, he said he would come in a few minutes. When he came, I was ushered in to his office. It was dark but well furnished. I sat in one of the sofa sets while he sat behind a table. He asked what I wanted exactly, then I told him. I handed him my papers. He scrutinized them. He got behind his computer and started typing. He printed a sheet and asked me if I had extra copies of my documents to leave behind. I didn’t. He offered to photocopy and went to the reception. When he came back he filed the photocopies and handed me my original papers as well as the letter he had addressed to the in-charge ART clinic. I thanked him. He asked me to come at the end of the week, on Friday if possible to be introduced to the staff of the ART clinic. He explained that they had already left since the clinic had already closed. I accepted to come on Friday. He walked me out to the reception and told the secretary to introduce me to the staff of the clinic when I came on Friday. He left me talking to the secretary and went back to his office. The secretary advised me to come at around 9:00a.m.

**28^th^ August, 2015**

On Friday, I set off for the treatment centre early in the morning and was there by 8:30 am. At 9:00am I was taken to the ART clinic by the secretary. She took me straight to the office of the in-charge ART clinic, said a few things about me and left me there. I handed the letter from the superintendent to the in-charge. He read through it, got up and told me to follow him. He started introducing me to the staff, starting with those at the triage. He then took me to the dispensing room and the records room. In the records room one of the staff challenged him to show him permission from the hospital administration. Smilingly, he handed him the letter from the superintendent. He kept quiet. We pushed through several people who sat in the narrow corridor to the counseling rooms. I was introduced to several counselors who were orienting 4 people about ART. The in-charge told me to engage one of the counselors if I had any more questions about the ART clinic, then he left. Since they were busy, I decided to wait on the bench outside their office. As I waited, two elderly men came and sat next to me. They were deep in conversation and seemed to know each other well. Shortly after a middle-aged woman wearing an apron on top of a long skirt and oversized blouse approached us. She picked a powdered substance parked in polythene material out of a big sisalbag and said, ‘*Ebisigo bya ovacado abyetaaze.Ffe abimila RV zituleeta ekifu ku maaso. Ebikuta bino bilungi nyo, ofuka ku kyayi nga majaani nonywa (those who need peels of Avocado.* (Is there anyone who needs powder from avocado seeds. We people who swallow ARVs get blurred vision. This powder is very good, you put on tea like tealeaves and drink).’ One of the men picked interest and asked for a packet. I asked him if he was sure it would help him. He said, ‘Mwana wange, obulamu tugula bugule. Ogezako kino nakili. (My child, we just buy life. You try this and that).’ He paid 2000/= for it. The other one told him he had seen several people selling seeds of Avocado as well. The lady asked me if I too was interested. I told her I wasn’t for today but she should give me her number so that I call her for some at a later stage. She checked her mobile phone and read it out to me. She explained that she was a client of the clinic, and always came to sell medicine to the patients. She left. I started enganging the two men. I asked if they knew each other. They told me they had met at the clinic. Both of them were still on Septrin. They asked me what I was doing at the ART clinic. I told them I was a student on research. I was going to be at the ART clinic for some months.

We were still talking when the counselor opened the door and called me in. We started talking about the ART clinic. I was told they had 3 days, Monday for mothers and children, Thursday and Friday were general days. They also had 4 outreaches, on the first Tuesday of the month, Every Wednesday (but they went to different places). By the time I was done with the counselor, the 2 men were gone and the clinic was practically empty. I told them I would start the following week and left.

**8^th^ September 2015**

It is my second visit to the treatment centre. I arrived as early as 7:00am, but I found the ART clinic still locked, although there were patients already waiting on the few benches outside. Most of them were women, I saw only two men. The health workers were yet to arrive. I was forced to stand outside for 30 minutes before one expert client/counselor arrived. I went in with her, placed my bag in the dispensing room. She asked me to help her identify files for the patients who had registered. At about 8:30a.m the chief nurse arrived and started picking ARVs from the store on to a trolley in the dispensing room. I asked her if she needed help with packing Septrin, then she said that it was out of stock. I later learnt that an ARV regimen CBV/N –constituting AZT/3TC/NVP (zidovudine/ lamivudine/ nivirapine) was also out of stock. Patients kept trickling in and by 9.30a.m the clinic was parked to capacity. The compound and corridors were all full of people. The available seats were not enough; majority of the patients were standing. The clinical officers who were supposed to review the patients that day had arrived and started seeing patients who had been triaged. At the triage, the expert patient announced to those on only Septrin ‘*olwalero kweteekamu cash*’ (you have to invest money in yourselves today, in otherwords you have to buy the medicine). She asked them to forward their books then she wrote the prescription and a retun date. They were told to retun after two weeks instead of the usual two months. Only patients on ARVs went through the full cycle but some reached the dispensing room only to find that their regimens were not available. These were advised to either buy or ask from a nearby mission HCIV. A gentleman asked where he could buy the regimen but he was given no definite answer. The nurse told him to go to Wandegeya a Kampala City suburb. An expert client, who served as a volunteer at this facility told some of the patients to go the mission facility and ask for the drugs. Many of them hesitated but he assured them that they would be helped. He told them to ask for a particular health worker (whom I later learnt was a fellow expert patient) whose name he told them. He went ahead to call and explain to him that he had referred patients who needed specific regimens and asked him to help them.

Patients who were new referrals were denied medicines. A particular woman seemed to be desperate to get enrolled but she was turned away and told to retun in a months’ time. I tried to plead for her but the nurse refused and explained that they could not take on new patients when they had failed to provide ARVs for those they already have.

I realized that it is easier for patients related to people who work in the clinic, but especially work related to the HIV clinic to get easy access to drugs. In one case a nurse collected medicines for her uncle call him Frank. The counsellors teased her about when the patient would ever come to the clinic, they told me they have never seen him, because she opened his file, drew the blood for CD4 testing herself, measures his weight and updates his file generally. She put him on option B plus which is preserved for (Pregnant women). The nurse told me she put her uncle on it because he is poor at swallowing medicines in the first place, so she could not risk giving him drugs with a high pill burden.’This is easier to use,’ she said. She shared that her uncle had confided in her when he was diagnosed with HIV but refused to come for treatment. ‘You see he is an elder here, he fears to be seen. People will start gossiping about him (*okumusoma*). He said that people will start questioning what a man of his age and status was looking for if they see him here and conclude that he has HIV, which he doesn’t like. I persuaded him to come and when he refused I decided to enroll him. He is like a father to me, if he dies how will I explain that I could not help? People won’t believe that I was unable to help in my position.’ When she came she was complaining that the owner of the medicine was not sure of the appointment and was telling her next week. She got his file and showed me the CD4 results of 70 cells/mm^3^. She exclaimed, ‘eee! You see, he almost died whilst I was looking. I started him on treatment last year.’ When she asked if his regimen was there the counsellor said it was were there and advised that she takes for him as much as for 2 months. She consulted and indicated the next refill date by herself.

We worked through lunch time without stopping until we cleared all the patients at about 3:00pm. There was no time to eat. I was so hungry but felt it was not proper for me to excuse myself to eat when none of the health workers got up to eat. I further understood how hard their working conditions were, when I asked for the toilets to ease myself. The nurse bluntly told me that the latrines were in a very sorry state and that they have overtime learnt to hold their urine until they return home. She nevertheless asked a colleague to take me to their home, which was nearby to ease myself. By the time the last patient left I was hungry and exhausted. The health workers however seemed to be comfortable, despite the fact that they had similarly got no time to eat. I imagined that they had got used to staying hungry the whole day.

**11^th^ September 2015**

I arrived as early as 7.30am and found the clinic still locked. The clinic started at around 9:00a.m. I opted to help with the dispensing of medicine, a task that I realized many of the counsellors and expert patients were not keen on taking on. It was almost always left to one nurse, who had to read the prescription, dispense medicines, interpret the days for return for the patients (some of them illiterate) and register their next date of return at once. It was too much work for one person, but none of the expert patients and counsellors all of whom served as volunteers was willing to help the nurse in this capacity. They preferred to help with triage and counseling. Today I realized that their actions could be motivated by monetary gain. Sitting in the dispensing room did not provide them opportunity to interact with patients, some of whom were willing to pay them to get favours, particularly a quick service. As volunteers they are not paid a salary, but hope to benefit from trainings and workshops. Although MUWRP had proposed to pay them, they were yet to honour the promise. I learnt that majority of them had no other source of income and relied on their position at the clinic to survive. They depended on allowances from workshops, tokens from clients in cash and in kind, money from clients to help them access medicines and other services e.g. health workers, and to minimize the inconveniences associated with collecting refills from the health facility. I observed that the counselors pick medicines for particular people, some of whom take months without stepping the clinic. Other patients are helped to jump the queue and avoid long waiting hours. For instance it is a routine that each patient is reviewed by the doctor/ clinical officer before they get medication. During the review, the doctor indicates/prescribes other medicines if the patient complains of other disturbances or OIs or the doctor identifies them. However, while helping in the dispensing room, counselors kept on bringing files and books of clients who wanted to skip this procedure and save time for other things. The nurse in the dispensing room was implored to fill the chart without checking the client and fill other details then dispense the medicines ahead of those whose books had been brought earlier. The medication in this case was taken by the counselor not given to the patient directly/openly to avert any complaints from those who had come earlier. Similarly those with lost books, paid some of these volunteers to help them access services. The latter would improvise pieces of paper and include the details of the patient and then follow them through the doctor’s room and personally bring them for medication in the dispensing room. Other patients who went through the formal channels without clear records were often reprimanded or disturbed at the dispensing window.

While in the clinic the volunteers can easily access medicines whether prescribed or not.
After the clinical officer asking her to test and they found she had a UTI one of them went straight into the cabins where medicines were kept and dispensed the prescribed drugs to herself.

Amidst scarcity – other people (especially staff of the facility or former ones in other departments) come for the few medicines for PLHIV. Today 2 women – who were identified as formerly working at the unit in records and as medical staff came and insisted they are given a specific ARV regimen. The nurse present put them off by insisting it was out of stock. She told me that the lady’s child had been raped so she was looking for prophylaxis. When she failed to access ARVs she asked for medicines for UTIs and went ahead to check through the medicines that were on the rack. She decided to contact someone in another health facility when she got nothing from here. She’d hardly left then a man from the laboratory came asking for an antibiotic called doxycycline. There were a few sachets left but they told him they were finished. One expert client came and checked everywhere to get a specific regimen for her friend but it was out of stock. She said that her friend had nothing to swallow. When she failed to get the medicine she said that she would ask from a nearby facility.

Patients try to negotiate with the dispenser to give them medicines they consider useful for them even if they are not prescribed.

Some of the medicines that were out of stock, were available in the store and had been ordered two days earlier but had not been availed in time due to bureaucratic procedures in the store department. The first batch of the clients we served received ARVs for only 2 weeks. Towards noon a few quantities of drugs were released then the remaining patients were given refills for up to a month instead of the usual 2 months.

A set of new staff has not received pay for the last 5 months i.e. clinic is crowded, seats are not enough, patients anxious and take a long time to understand sometimes what I also found to be rather confusing instructions about how to take medication. Staff don’t wear uniforms, many of them don’t have them

There is no privacy at the triage. The table is placed in an open space at the verandah. The patients’ names are called one at a time for their measurements (weight, height, blood pressure, middle upper arm circumference (MUAC).

**22^nd^ September, 2015**

The clinic began late at around 9.30am. Several of the clients who missed out on ARVs and Septrin two weeks ago came back today. I talked to several of them about how they managed to access them when they were called to collect medicines from the dispensing room. Some of them said they did not get any solution and so stopped swallowing when the extra tablets they are given got finished. They said that they had no money to buy. When I suggested that they could have asked from another facility like the mission HCIV, they were quick to mention that they did not know any one at there and so feared to go there. One gentleman said for him he knew no one there, but he went and begged the *basawo* (health workkers) until they gave him. Others said that they were referred by the expert patient and they were helped. Another gentleman said he went to Kampala and bought the medicine. One woman said she talked to a sister whose friend is a nurse in a public health facility. The sister talked to the nurse then the nurse got her the medicines. Hajji said that he got the ARVs for himself and his wife Faridah through his friend the in-charge of his local HCIII. ‘I went to him and showed him our books. He wrote the medicines down and said he would talk to a colleague. The following day he got us doses for a month. He is always helpful. When we are told to buy medicines from here, I always check with him if they are available at his facility. I go with the books and ask him to check if the medicines prescribed are available. When they are there he gets them for us. Because of him we don’t suffer even when we have no money.’

The stock-out of ARVs and Septrin has been sorted. Septrin and ARVs of different regimens are available in plenty. However several patients are prescribed other medicines that are out of stock. These range from antimalarials, pain killers, antibiotics and antifungals. Forinstance today amoxycycline which is commonly prescribed for the patients was out of stock. The nurse kept putting a star against it and asking them to buy it.

**25^th^ September/2015**

I counseled a client who broke down when the nurse asked about her age. She had been called to collect her medicines from the dispensing room and in the process of recording the extra medicines she had been given the nurse asked for her age then she said 25 years. The nurse thought she was older and expressed her doubts to her. She then asked her the year she was born, but she didn’t seem to know.When the nurse insisted the lady just broke down and started crying to the surprise of the nurse. At this point the social worker in me realized there could be more deep rooted feelings that the nurse’s question had aroused. I decided to take aside and talk to and perhaps reassure her. We moved a few metres away from the crowd and sat in the grass. I once again introduced myself to her and assured her that I wanted to help as much as I could. It turned out to be a very long story. Amidst tears she told me of how she grew up without parents and how she her husband of 5 years had passed on recently and left her with two children. His relatives had chased her and the children from the husband’s home and she was now living with a friend but she was struggling to fend for the children because she had no where to leave them, to look for work. She had learnt about her HIV status after her husband passed on and was yet to accept her situation. She was still bitter that her husband had infected her, because as she said she was a virgin when she met him and had never cheated on him. I encouraged her to be strong for the sake of the children and promised to link up with her for further exploration of options for improving her situation. Since she had no phone, I challenged her to look for me when she felt comfortable to invite me to their home. She was calm when we finished the talk. I took her back to the dispensing room and handed her the medicines that the nurse had put aside and bade her farewell. I later explained to the nurse the emotional turmoil this client was in, although she didn’t seem to buy it. In her opinion the patient was pretending because she had been caught lying about her age.

We observed several mishaps in the prescription of ARV medicines to some patients. In one packet, a client was given efavirenz mixed with another drug. Another patient was prescribed efavirenz twice a day when it is supposed to be taken once. The good thing was that these were new patients who had only taken the drugs for two weeks. The nurse did not expect that the mistakes had caused them much harm. The mistakes were attributed to tardiness due to large patient numbers. Like I earlier observed, the nurse who dispenses medicines usually works alone without help, such mistakes are bound to occur.

The process of dispensing drugs was halted for about 30 minutes when the nurse I was helping to dispense medicines received an urgent call and stepped out. I took the opportunity to move out of the dispensing room and observe what was going on in the other units of the clinic. When I opened the door, a young woman- call her Peace who appeared to be in her early 30s was standing in the corridor facing the dispensing room. She seemed desperate and anxious. She asked to talk to me and told me that she was looking for one of the counsellors call him Badru, and that she needed his urgent help. When I inquired about the help she hesitated to tell me. I knew it might have been a private matter and went inside the file room to get him. He went with her to the file room, got her file, walked her to the triage and took her measurements. He then entered one of the treatment rooms and asked the clinical officer to see her, which he did. He then came to the dispensing room and personally packed her medicines and asked me to record her date of return in the diary. As I checked her book to record the date, he explained a few things to her and left. As I returned her book I jokingly asked for the magic she had used to get through the process quickly. Her response was, ‘*Kisa kya musawo Badru* (It is the sympathy of healthwork Badru).’ She explained that she had just sneaked away from work to come and pick her medicines and thus needed to rush back quickly before her boss noticed her lengthy absence. She noted she was working as a secretary for some Indians and that she feared to disclose because they are known to be discriminative. She shared that she always tries to come and her counselor Badru helps her to get treatment quickly.

‘I always try to do the work assigned to me very fast to create time for coming to the treatment centre. When I come, my counselor Badru helps me to get through the process quickly so that I run back to work before my boss complains. But many times, the boss gives me one assignment after the other and keeps monitoring my progress. It becomes impossible to leave. In such cases I call counselor Badru and ask him to collect the medicines for me, then I pick them from him after work.’

She said that she just reminds him of her number then he retrieves the file and collects his medicines and then links up with him later to collect them. Asked how she knew Badru she said that he was her friend. He had counseled her when she tested positive and enrolled her on treatment.

When Peace left I waited for the nurse for the next 20 minutes then we resumed serving the clients. We worked until 4:00pm.

**17/September/2015**

At the triage almost everybody is coughing and the nurse is worried about catching tuberculosis (T.B). One lady who looks really weak is wearing an oversized jacket and says she’s been coughing terribly since last week. The nurse screams, ‘*nyabo genda ogule akatambala* (madam go and buy a handkerchief)’, but she keeps coughing in the jacket. She also suspects that she has T.B. The nurses are generally worried about catching T.B. This is the same observation I made in the other treatment centre. It is not the same with counselors who talk freely with patients. Even the counselor in charge of T.B. patients at one of the facilities always talks about the risk but you find him interacting with patients without a muff (mask). I wonder if its ignorance about the real risk of transmission. At the triage it is important to get interested in the patient’s story some of them need urgent help. I observe that the nurse is too busy to get interested in stories (she normally works alone). One 25 year old has social problems. She had skipped two appointments due to what she said was lack of money for transport and was immediately referred to a counselor for further adherence counseling. When the counselor engaged her and realized her problems were more deep rooted, she decided to involve me. I had been discussing with the lead counselor about the need to help patients beyond emphasing the importance of adhering. Several of the lower staff did not immediately understand my profession and continued addressing me as ‘musawo’ even after I reminded them that I am a ‘social worker’ and a mere student (that is how they should treat me). Most of the counselors here hold certificates in HIV counseling and so tend to empahsise adherence without discussing the contextual issues that impinge on the patient’s ability to adhere. I was always discussing this with the lead counselor. So when the patient shared the social issues that were interrupting her treatment the counselor thought I was the right person to intervene, even though I had no tangible resources to give. I nevertheless talked to the patient and we came up with an action plan.

In the process of engaging her I realized that she had forgotten to report the child’s cough to the clinical officer probably due to distress. I asked what she was going to do then she said that the boy would stay like that until the next return date in December. I got concerned and decided to talk to the clinical officer and request him to review the baby. He accepted and told her to join the queue once again.

Because of seeing me talk to several people, many patients including those who have not come to the HIV clinic-which operates in the open adjacent to the general OPD- kept coming to inquire where they should start from instead of going to the triage at the OPD. Another asked me what to do because she doesn’t want to be pregnant yet. I referred her to the counselor. The implementing parters recommended that the patient sits next to the nurse but the nurse is not willing because she fears catching TB.

There are several people who are still pushed by their relatives to test for HIV. These devise ways including conniving with doctors to help them convince the patient to do an HIV test. An aunt to the patient came with a baby of 2 days old and said that the mother was still weak, but she insisted the baby is taken for checking where she has been receiving antenatal care. She and the sister who came said they heard that there was medicine given to babies of a positive mother immediately after birth so they decided to bring the baby as soon as possible.They said they had not managed to bring the mother to deliver because the labour started suddenly. She delivered from a private clinic but the doctor was informed about her status then he said it was okay since she was taking her medicine. The sister explained that the patient was married to some man in Bunyoro but left and returned home. She started getting rashes and falling sick every now and then. They asked her to test for HIV, but it is something she could not hear of. They then connived with some doctor who referred her to a treatment centre for tests without identifying them. She ended up here. She was counseled and had to accept the test. The results turned out positive and she was enrolled on medicine (ARVs). At first she defaulted, but an illness that almost killed her woke her up. She is now more positive. She met her new husband and left home. She said that she was not sure if she had disclosed to her new husband, but ‘her remarks, “I wonder what we shall tell him to give us money to take the boy to the hospital,’ shortly after made me doubt whether he was aware about the patients’ status.

I met several people who had skipped appointments due to lack of money.

It is a common reason that patients give for missing appointments. It is critical to meet routine appointments that are more or less compulsory to attend. Skipping them usually earns one reprimands and stress at the triage.

Then several others return for the subsequent visit without buying the medicines they were prescribed and told to buy because they were unavialable on the previous visit. The nurse always points this out at the triage and asks the patients why they did not buy the medicine. Almost all the patients I have seen mention the lack of money. Sometimes they are waiting for a relative to provide then they delay.

Weight is measured on every visit and one has to explain stagnant or lost weight. One woman said she had been stressed lately and that is why he she had lost weight. Patients are bit defensive when the nurse suspects that they are not feeding well. One woman said that she eats well even though her MUAC was yellow- an indicator of malnourishment. The nurse advised that she should eat eggs, take milk and other proteins, then she retorted that she ate all that.

**21^st^ September 2015**

I helped a bit at the triage and then joined a group of 4 patients who were going to be taken through a counseling session before initiating ART. I was introduced to the patients as a student who wanted to learn about the counseling process and that I posed no harm. The counselor informed me that two of the patients were attending the orientation for the first time, whilst the rest were attending for the second time. I understood that each patient was supposed to attend three times before beginning the medicine but the rules were overlooked when a patient was in a critical condition. Many of these were started on treatment immediately. Only one patient had brought a treatment supporter- her friend- to attend the orientation. The counselor observed that they were no longer so strict on implementing the rule of bringing a treatment supporter to attend. He could however cite no specific reason.

The counselor starts by explaining HIV, CD4 (soldiers) and the relationship between the two and how HIV weakens the body. He follows a chart with visual images that the patients are able to see. He then explains what the medicine does and then reminds them that this medicine has rules. ‘Our medicine cannot be mixed with herbs. You have to take the medicine everyday at the convenient time you have chosen. It is not for playing around with. Our medicine doesn’t augur well with alcohol. If you have been drinking alcohol, you will have to stop.’ At this point one of the clients inquired if one bottle of beer posed any harm. The counselor said alcohol could make the patients to forget their medicine and so it was safer to just stop taking it.

He continued, ‘You have to eat well. A balanced diet is a necessity. This does not mean eating meat or chicken but locally available food if well mixed can suffice.’ Whilst sifting through the chart he talks about the different categories of food, proteins, vitamins and carbohydrates. He further notes that they are expected to have enough rest, overstraining is not good for them. He then promises that if they follow those rules they will be okay. In his words, ‘eat well, take your medicine, don’t over strain, you will be well’.

Lastly he talked about the potential side effects of the medicine. He distinguished between serious and non-serious side effects. The former included burns, jaundice whilst the latter were headache, scary dreams and dizziness. For headache he said the patients could buy pain killers or seek care from the nearest health facility, whilst the dreams and dizziness would subside over time. He told the patients to come to the health facility immediately they experienced the serious side effects.

He opened up the session to questions, but none of the patients seemed to have any. He got their newly opened files and recorded that they had had the first and second pre-ART counseling sessions, respectively. He then informed them that they were expected to return the following week for the second and final session, respectively.

**4th October, 2015**

I had an appointment to visit 41 year Maria old at her home this Sunday. She preferred evening time after church. I set off from my home after 2:00 pm and stopped several times to call for direction. I finally found the place. I met her standing on the side of the dusty road. She directed me to park along the road. Her house had no access and was sandwiched between several others. She pointed at the back and showed me her father in laws’ house. She explained that her piece was given to her late husband by his father and that one of her brothers in law inhabited one of the rooms in their house. The house was on a small piece of land without a compound. It was made of mud and wattle and in a dilapidated state. The iron sheets are rusty and have gaping holes, the sides of the house have holes too. Maria tells me she once found a snake in the house, they are now careful to check around before sleeping. She said that she doesn’t have much land to dig. The land is highly fragmented. She tells me that part of the land she used to cultivate was given away to the son of her in-laws when her husband passed on. She tells me she is however able to grow food for her and the children on church land. Asked how she managed to utilise the space considering that much of it was fenced and patrolled she said that she was given permission by the lead priest of her local catholic church. She explained that being a member of the church choir she had opportunity to interact with the clergy on a regular basis. She just thought of talking to the priest about her predicament and ask him for land to cultiavate. When she shared with him, he was sympathetic and told her to get a convenient piece. The kitchen and latrine were both makeshift. She complained about the poor state of her house as she ushered me in. As if worried of seating me inside she mumbled, “I wonder if these children’s house is neat’. I sensed her fears and immediately suggested that we sit outside which she thought was a good idea. I was later shown inside and found that they had just a living and one bedroom. The living room serves as store as well. It was a mixture of chairs, sauce pans, shoes, books, jerrycans etc.

I was given a small wooden stool whilst Maria sat across on a mat. She called her two sons who were around to greet me. She said that 2 of the children were in boarding school, whilst the rest lived with relatives. I observed that the younger of the boys was jolly and very close to his mother whilst the bigger boy kept arguing with her. Before I started the interview, she told me about the bigger boy and how she and him had disagreed about his academic fate after he failed S.4 miserably. She said that he got 68 out of 72 but had refused to repeat even when she offered to change his school. She described him as rebellious citing his refusal to share his mock results with her earlier in the year. ‘*Azanyira mu sente zange ezekinaaku*’ she complained. She asked me to counsel him first before proceeding to the interview. When I engage the boy he tells me that he wants to study but he does not want the school his mum suggested.It is just next door and his mum thinks it will reduce the stress related to moving to and from school. The mum interjects and notes that he has been coming back home late at night because he is watching football. She said he loves arsenal and believes that is why he is not concentrating in school. The boy’s argument is that he has networks and students in his old school are more serious than in the one his mum is proposing. I advise the mum to let the boy to go to the school of his choice and explain that that’s the only way he will put in effort to pass and prove a point. The boy tells me he would prefer to study mechanics but his mum says he will need a lot of money about a million and good academic documents. Whilst I engaged the son Maria grabbed a meal of beans, posho, rice and pumpkin. We began the interview when she completed her meal.

**6^th^ October 2015**

The clinic once again began late. By 7.00 a.m. people were seated. The clinical officers came in after 8.00 a.m, but the actual work began after 9.00 a.m. In fact it was coming to 10.00 a.m. The expert client/counsellor in charge of the triage today began with a prayer, then a talk about adherence and then proceeded to triage. As usual she always suggests that those who are not sick should go straight to the dispensing room, rather than lining up to see the doctor as a way of cutting time. Since the second clinical officer was not yet there, the dispensing nurse had to do part of the work the clinical officer would have done. She was reviewing patients and filling their charts, counting their balances, indicating the date of return and dispensing medicines at the same time, a work load I thought was too much for one person. Indeed many mistakes in the prescriptions and dispensation were made by both the clinical officer and the nurse. Several books came from the clinical officer when the combination was changed. It was often sharp patients who noticed and alerted the nurse that the medicine looked different. One patient pulled out her tins to show the nurse that what she was taking was different and that is when the nurse checked back in her book and corrected the mistake. The nurse also misread the correct prescription several times and was alerted by the patient.

In one case the clinical officer came to ask about two medicines packed in different sackets. Both were orange, but the bigger one was of a darker shade than the smaller one, but both are Efavirenz. The client was on TDF/3TC/EFV, but only efaverenz was dispensed which means she was over dosing, by taking the same medicine twice instead of once. This was a new patient. Good enough the first timer is only given 2 weeks before returning.

Some of the patients on Dapsone were prescribed Septrin by the clinical officer. It looked like in the process of rushing to manage the large number of patients, they got no time to check the records of the patient in the books. Luckily the patients were keen to notice that they had been packed Septrin instead of dapsone which is significantly smaller in size and round rather than oval in shape like Septrin.

Instructions for taking medicines were being given so fast. Sometimes the regimen was changed a bit because of scarcity of a specific drug which confused the patients. The nurse was complaining about the inadequacy of Lamivudine (3TC) and Zidovudine (CBV combination). This combination is given to particularly 2 categores. It is combined with [CBV – N] but also with Efavirenz. So those on CBV/N were given the CBV/N that is already combined (3 in 1) to save some CBV for those on CBV-EFV. Thus a patient who was taking two separate tins was now given one. Some of them hesitated to take the medicine. It took minutes of explanation to convince them. Others were however more trusting. They neither protested nor asked about the reasons for changing. They just nodded and said, ‘I have understood *musawo*’.

The long waiting time creates despair among the patients but also the health workers tempers flair as incidences of rudeness on either side are not uncommon. One patient attacked a nurse screaming *(she looked sick and later told me she had headache),* “ You do not know how to handle people, we have been here since morning, I personally came at 6:00 am without eating because I thought I would be worked on early and go home. Look it is now 1:00 O’clock.” The nurse fired back by jeering and saying ‘stupid’. She too was complaining of headache after working for long on an empty stomach. She was tired because she had worked for long the previous day in a clinic where she decided to get a part time job to make ends meet having been not paid for 5 months. She later told me that particular client was quarrelsome and rude. The counsellors present nodded in agreement*.*

We were still discussing the patient when one counselor received a phone call and the person on the other end was informing her that he had arrived and that she should go and pick his book from the gate. She got up and left and returned with a neat book with the clients’ registration number. I inquired about the patient- call him Ronald and why they could not reach the clinic. She said that this client was afraid of reaching the clinic. He did not want to disclose to anyone, not even relatives and so feared that if people saw him at the clinic news of his status would spread. She had counseled and enrolled him on treatment four years ago and when he explained to her his situation she decided to help out. The arrangement was that he came up to the gate and called her to pick the book, then she picked the medicines. He linked up with her to pick them later in the evening. Asked how the patient’s diagnostic records were updated, the counselor said that she always notified him when the tests were due then he came, but he stopped at the entrance then she took him a filled laboratory request form. The laboratory serves all the patients of the health facility so it was easy for him to sit there without anyone suspecting what tests he is doing. Reading from the number on the book, the counselor retrieved Ronald’s file, updated a few things, consulted the nurse dispensing medicines about the days for the next refill and indicated in the book, then picked medicines for three months and put them in her bag together with his book. I noticed that Ronald had inserted 5000 shillings in his book for the counsellor, which she picked and pocketed.

**12th/October/2015**

The OPD on the general side is unusually full. The mothers and their children trickle in bits. A number of about 20 is expected but they end up being 60, because patients who had missed appointments due to lack of transport and other reasons also turn-up. While at the triage, I discover that the forms for CD4 count request I usually fill are missing. The nurse is alone as usual and the screen for shielding the clients at the triage from the prying eyes of others is missing today. I ask about the unusual scenarios. For the CD4, reagents are out of stock (*since Thursday last week*) and the patients won’t be tested until further notice. One patient who had come particularly for CD4 was sent back and told that ‘they’ would be informed. According to the nurse the IP) has not yet delivered although they received an order sometime ago. For the screen she explains that the place where it is kept was still locked by the time she arrived. I reached at around 8.30 a.m. over 20 patients had been screened at the triage and sent to the clinical officer by then.

I met 3 patients of interest to me. One (call her Liz) who skipped an appointment and had to answer the tough questions of the triage nurse. She claimed her son of 1½ had been on drip and she had no one to send for her medicines. The nurse asked her for proof. She was well armed with a medical form from a private clinic. That is how she got off the hook. I took some minutes talking to her about her experience and especially how she found out her status and why she treks all the way to this treatment centre. She was lively and smiled a lot. Looked good too and appeared not so needy. Initially I had asked her about her husband then she noted emphatically ‘*taliwo* (he is not there), y*agenda* (He left).’ She says she moves all the way because she expects a better service here. She narrates that she had delivered her 1st child from a nearby facility but the care had not been the best. There was one nurse attending to many mothers each with a different need some ended up neglected. For the 2nd pregnancy she decided to come straight here. This is where she turned out HIV positive and got enrolled on ART. She has not told many people about her HIV status apart from her mum (with whom she stays), dad, siblings and her best friend who also shares her secrets with her and is more discreet than most of the women she interacts with in the stone quarry where she works. She says that people often back bite other people on drugs they know. ‘*Sagala kunsoma nga bwebasoma abalala* (I don’t want to be gossiped about like it is with other people). They often say, ‘*oyo gundi omulaba, yafa dda, lwakunyirira, ali kubikerenda* (you see so and so, they are already dead, they are on medicine (infected with HIV), even though they look good).’ (I realized this could be another underlying reason for taking the long distance) she says her lifestyle has changed since she found out her status. She always tries to keep money in the house. ‘*Omutu alina akawuka sawa yona alwala* (an HIV infected person can fall sick anytime).’ I also need to have enough to enable me come for refill when I am expected to. I always strive to save my money now. Back then ‘*zenakolanga wo zenalyanga* (I used to consume whatever I earned).’ I would party with my friends, drink alcohol, dance etc. I also strive to eat well. I know that a PLHIV needs food to keep the body strong (*atalya bulungi agwamu mangu*- when you don’t eat well your health deteriorates fast). We are expected to eat greens, milk, 3 times and a day. I strive to eat well because I work hard. My mother also helps me with food since I told her.

She said she disclosed to her family members and they are very supportive, but the father of her child doesn’t know. ‘I can’t fail to get food to eat and transport. I disclosed to my family. These people remind me when to come here and send me mobile money to cater for my transport. I mean my parents, dad and mom then my siblings brothers and sisters because I opened up to them very well. They even tell me when you are about to go to hospital please notify us and when I tell them they contribute money for transport, user fees and anything else I tell them.’‘I don’t have much problems with sickness. The Septrin I take helps me a lot. I know it heals any illnesses, cough, flu, head ache. I take only Septrin, no other medicine. I work in a quarry but never cough or catch flu. If I get flu, it will take at most three days to heal.’ I raised a concern about the nature of work she was doing for her health. She agreed it was not good for her and told me she was working for capital to get better job *(knowing that T.B and HIV go together often*). She said that she had kept a good sum from a truck she recently sold.

I also met Joweria at the triage. She is thin and sick looking. Drags her feet and can not walk firmly. The lips are dry the skin pale, breathing very fast and she gasps for breath as she talks. While on the bench waiting for medicines I sit and have a chat with her. I ask her who she came with and she points at her ‘ideally maternal cousin’ although in Kiganda culture she is her mother (a daughter of her mother’s brother) (*Her mother is a paternal aunt to this lady*). We start talking about her experience with HIV. I wondered why she looked so sick and she said she has a chronic heart problem (*entununsi* – palpitations) and ulcers. ‘Those are my illnesses,’ she says. I was staying in Mengo renting a house with my daughter who was working there. I used to earn a living by frying a few eats and selling them around the hospital. My daughter took me for check up in Mengo and they found that I had a heart problem. I would get the medicines from her I did not even have to go to the hospital. The medicine used to find me at home. Recently at the end of last year, I fell very ill. My daughter had been transferred to Mubende so I had no one to stay with. Together with my daughter we talked to my aunt, who asked me to come home. She is now blind but her children help me at home. That mother of mine lives in the neighbourhood a walkable distance. She always checks on me. The mother found us talking and joined the conversation.

**Mother**

She came because of illness I brought her for a test although she had objected saying she had no HIV. I know how people with HIV are, I don’t hide, I am also on HIV medicine. I encourage her to move on, it was a clinic day and I kept on showing her people moving to the table to register and told her all of them were sick. *Ensi yona ndwadde* (The whole world is sick). She was started on Septrin but on our next visit she was retained. The doctor said she looked weak. They gave her 2 bottles of water (IV) and then they decided she should be admitted. I called her son and her daughter (who is still in school) to keep her while I went home to pick stuff. I also informed her aunt and her father. It is her father who met the treatment bills. He sent all the money we needed. He did not come to visit because he had another ill child (her brother who was admitted at Mulago but eventually died). But he sent us all the money we used. I would get it through mobile money. Even the transport we use, it is sent on my phone then I collect it. . She got four pints of blood and several drips of water. We bought all the medicines from the pharmacy. I always come to check on her everyday but sleep home. Her aunt also gives her money when she can. Both her father and aunt have rental property in town. This is where they get money to sustain them. She got enrolled on ART from the admission bed. I and her were oriented. She vomited at first and got dizzy as well. But I told her to hold on because we had been told that such things happen. I went to a clinic nearby and got medicine for vomiting then she stopped. I also ‘imagined that the side effects would have happened because she had just been transfused. Even the palpitations. She got medicine from here, which she has been swallowing but it is now over. Joweria tells me she feels bad but did not tell the Clinical Officer about it because he was rushing to write and did not ask her if she had any problem. The aunt says they will seek help from clinics nearby in case anything goes wrong. In the aunt’s opinion, she is better, she was told to take lots of fluids and she does. She can move around. Whilst I talked to the aunt, she asked to lie down that her legs were paining. She went to the grass. The aunt had bought her very cold water initially to which I objected and asked to buy room temperature. The patient was shivering so I thought cold water might compound the problem.

They use taxi from home to the road and then bodaboda to the health facility. The mother looks good. She said that she and her husband were living positively.

I also met grand parents who were taking care of grandchildren living with HIV. One was an old man who had brought his grandson for refill. He had ridden 8 miles on a bicycle to the treatment centre starting the journey as early as 6:00 am to arrive as early aspossible. The other was an old woman with a grand daughter who were escorted by a white woman and Ugandan man who brought them in a car. On an earlier visit, this old woman had come with two white women. She tells me that they returned home and gave these 2 people the responsibility to take care of the girl. They belong to a church based organization and were looking for a sponsor for the girl. She tells me the girl was given to her by her son after he disagreed with the mother. She was 1½. She is been falling too ill till people from this organisation advised her to test her for HIV. She was found positive.

I asked if the father is aware she said no. He has abandoned the girl with her, even when she started school, he promised to bring fees, and he never turned up. Today he has not come. The old woman makes mats and also lobbies the head-teacher to keep the girl in school.

**24/November/2015**

This sunny Tuesday morning I arrived at the treatment centre at about 7.30 a.m and decided to stay in the car till 8.00 a.m. The place is rather deserted, only one car in the parking, with mine they became two. I see people trickling in one by one. At exactly 8.00 I get out of my car and pick my bag and walk to the ART clinic. On my way I meet one of the expert clients. She asks which of the two cars is mine. I point to it. She then asks what time I plan to leave back for Kampala. I told her about 1.00 p.m. She asks if I can give her a lift. She has to go for a refill at JCRC – Lubowa. The scheduled date is Thursday 27^th^, but she reveals she wants to take the opportunity of a free ride to town so she will convince them to give her the drugs. She notes she has been broke lately. Later she reveals to me how she plans to convince the health workers at JCRC to give her medicines. Her plan was to tell them that she is travelling for days and would not be able to pick her medicines. She was confident they would give her the medicines. We moved together to the clinic and found the patients deep in prayer. At the clinic I found that one of the nurses was unusually early. The patients had already assembled, not very many though so far. The triage was set up and had files and the weighing scale but the Expert Client/counselor was inside, sorting some files. The nurse was arranging medicines on the trolley. She later told me that Septrin was not packed. I inquired why, then she said that one of the expert clients had not turned up yesterday (Monday) to pack them. Some Ministry guys looking for data had been around. They also helped them to organize the record books especially the dairy (booking). IP staff had also come and complained about the absence of the volunteers. They are planning to train them next week and start paying them. They are more interested in expert clients but because they are few, they plan to take up the volunteering counselors (they will be expected to come everyday) as well, a total of about 8 people (4 Experts Clients and about 4 professional counsellors). Her own salary is expected in 3 weeks time (but she notes her hopes are not high because they always promised and never act. We were chatting as we counted and packed Septrin for one and 2 months respectively, when one volunteer of the counselors walked in saying she had a problem and needed 100,000/- urgently because the electricity guys were going to disconnect her. She starts asking each of us for any money. The nurse said she did not have money.

Meanwhile the triage had started. The expert client/counselor manning it started by asking one of the patients to say a prayer like she usually did. She then started giving the health talk. She is talking about (swallowing medicines) and on top of her voice screams ‘*mulina okumira eddagala mpozi nga munafa*’ (you have to take the medicine, unless you want to die). She then tackles the subject of disclosure and urges the patients to be open about their status and to prevent infections among young children. She asks if there is any question. Then one person complains that they are given (sometimes) days of return on none clinic days and are sent away when they come. On hearing the complaint the nurse was offended and got up to go and clarify. She asks if anyone has ever been denied medicines when they came on days other than on the day they were scheduled! The patients quickly said ‘no’ in chorus. The complaint was overruled. But the expert client advised them to come on the nearest clinic day and they will be helped. Another question was raised by a middle-aged women wearing busuuti. She asked the expert patient what they should do to prevent being rejected by husbands when they disclose. Her question sparked murmurs among the clients, some of whom started arguing among themselves. I decided to move closer to capture the mini discussions. I got my chair and sat close to a group of women. I realized that there were two camps, those who insisted that disclosing to a spouse was ‘stupid and immature’ and those who ridiculed the former because they felt it was important to disclose. One woman in a discordant relationship openly pitied those who did not want to disclose. ‘*Mbasaasidde!* (I pity you!),’ she said. How can you sleep with somebody you have not told your status? You are murderers. It’s better to open up and face the consequences. Who knows, they may be supportive. Like for me, it’s my husband who brings me here, even though he is not infected.’ Citing the concern raised by the client who had asked the question, one of the women seated next to me- call her Stella, justified her decision not to disclose to her husband to me. ‘*Wama musawo*, it is sometimes difficult to disclose especially if you want to get married. I have been on medicine for a while now, and at first was open about my status to the men who expressed interest in me, but all of them took off the moment I disclosed to them. I decided to grow up and didn’t inform my current husband. It is now three years and we are very okay. I inquired how she managed to continue on treatment undetected. She said that she hides her medicines and comes to the treatment centre after he has gone to work. She said that taking her medicines when her husband was around was at first hard. “You see musawo, I had been taking my medicine at 8:00pm when I met my husband and it was a convenient time for me then since I was living alone. But when we started living together it became difficult. I had started defaulting. He normally returns from work by 7:00pm and watches television. We rent only one room. It was difficult for me to retrieve the medicines from under the bed, where I hid them, on a daily basis. He would have wondered what I picked from there everyday at almost the same time. I decided to keep the medicines away from home. There is a neighbor who is my friend, she is also on HIV medication like me and lives alone. I asked her to help me keep my medicines then she accepted. But now when it is time, I just walk to my friend’s house. It is a few houses away.”

The expert client eased the tension by encouraging the patients to bring their spouses to be counseled and tested together with them. She said that their counseling would convince them to remain in the relationships. She proceeded to call names of patients for triage, but the discussions about the issue continued among the clients with several of them insisting that they cannot risk disclosing to their spouses.

As usual there is no confidentiality at the triage. Personal details like age, reasons for skipping appointments are asked for openly. I observed that the expert patient was given tokens by supposedly satisfied clients. She in particular always receives gifts from patients. She is their big advocate and often comes up with pragmatic solutions e.g. she always persuades the nurse in the dispensary to work on some files to cut on the load and consequently long lines when patients indicate they have no complaint. The volunteers kept bringing in files and books or only books- some new and written in by them- to get medicines for ‘their’ clients. Among the clients brought by one expert client was a lady whose file and book indicate that she skips appointments. She last did a CD4 test (CD) in February last year. The nurse insists that she is given arequest form to do CD4 and CBC. The lady objects saying she will do that next time.Her records show that she was supposed to come in November last year but has just surfaced. She does not even remember when she last did ‘CD’ test, she claims 3 months ago, but there is no record at all. She explains that she left her little child at home with young children and needs to go back very fast. The nurse refuses to work on her and sends her away bitterly. I get concerned to find out what could be wrong. In this context a patient who skips appointments or is found not to adhere is no one’s friend. They are normally judged and criticized for not being serious without a hearing. Reasons normally fall on deaf ears. I decided to talk to the woman and ask her why she skips appointments privately. Well she told me that she is on Septrin and thought she did not need to come when she felt okay. She said that she buys the Septrin from shops near home. I realized she did not really appreciate the importance of measuring her CD4 routinely and being reviewed every two months when I asked her to explain the reason why she has to take Septrin then she responded that it would improve her CD4 count. I decided to explain to her the importance of both Septrin and the tests. After my talk she accepted to go for the tests and was eventually given medicine.

Then came a young woman of 22 years who lives in over 50 kilometres from this facility. I asked why she had to come all the way, then she said that she would have gone to a nearby facility buther mother- in- law gets her medicines from there, and may see her in the line. I asked about her husband then she said that he did not know she was on ARVs. She learnt her status during antenatal visits and feared to inform him. I asked how she managed to keep her status secret then she said that she hides the medicines deep inside -at the bottom of the baby’s suitcase where the man has no interest or access.

I complain about how illegible some names on the patients’ books are as I call patients to collect their medicine. One woman tells me she does not know how to write and several others said the same. This gives me an impression of how hard these people’s job is. Explaining things that are written to people including the dates of return; schedule for taking medicines etc and whether the quick manner in which they are explained really helps. It is no wonder that I often find patients seeking for a cousellor for further explanations, but many don’t. One of the girls who was oriented 2 weeks go by an expert client did not bring a treatment partner. Actually they were 3 that day who were studying for the last time but had not brought a partner at anytime. I had attended the orientation session with them and when I inquired why they had come with no body they told me they had a spouse (1) and sisters (2) who were already on medicines (ARVs) so they would guide them. The eexpert patient/counselor who was orienting had told me that they were no longer so strict on the guideline. Today one of them came complaining that she had got confused and messed up the medicine. The husband was not willing (*interested in*) to help her and probably because he too didn’t know what to do. I realized the importance of having access to someone who is knowledgeable on HIV treatment in promoting adherence.

Patients negotiate for medicines they consider important to have but are not recommended by the doctors e.g. a middle-aged woman who asked for multivitamins, after she was given ARVs. So the nurse asked which vitamin – she described them as green in colour – (multivitamins are commonly given to PLHIV on ART because the ARVs usually cause loss of appetite). I asked why she needs them; she said, ‘*bulinga kanyebwa* (they are like my nuts).’ Seems she considered them as an escort to ARVs.

I left the clinic at around 3:00 and gave the expert client the lift I had promised in the morning. A long the way we talked a lot about her health / experiences and the clinic history.

When asked about the resources she considered important for the management of HIV in the home she said that she thinks at home food and water are important resources because without them one cannot take medicine. She observed that many of them have to buy food because they have nowhere to dig. We arrived in Kampala City at around 4.30pm due to atraffic jam along the way. When it was clocking 4:00 O’clock she called ‘her’ counselor and alerted her that she was coming but was delayed by jam. The counselor promised to wait for her.

**26^th^ November, 2015**

I came very early today. I found the clinic already full of people. The triage table was covered by a screen and the nurse was working alone on a heap of books. The ones triaged were a heap and those yet to be touched were slowly heaping up as well. I joined her at the table. I started reading the books for her, even pointing out those whose appointments were earlier or later but skipped or very early. The in-charge brought her some long strip which was supposed to measure the arm circumference (MUAC). She complained bitterly, that it was a lot of work for one person. Indeed when a counselor came and attempted to measure, it would take about 5 minutes or more for her to finish one person. Later the counselor gave up and decided to help with the writing as I read the names and nurse measured the patients’weights. The books moved faster. A total of 143 patients were worked on. The customer care officer kept on checking and pulling out specific books noting that they wanted private. The nurse was suspicious that those were relatives who were being helped to jump the queue. Later a lady whose book had been pulled out came and said she had been asked to weigh herself, but the nurse refused her and told her to bring her book. She never came back. Another lady’s book was ordered for by a clinical officer. The nurse started complaining and told me that those were either relatives or money had exchanged hands. The counselor confirmed that the clinical officer knew that lady. They were friends / acquaintances. The nurse was telling me that it was unfair for other patients to line up. Several patients who had skipped appointments came today. Many of them said they had travelled far but still had no medicines to swallow. The Records Assistant is trying to iron out the problem and harmonise the appointments between Thursday and Friday as well as to reduce on the balances accumulating. They believe it’s the cause of a high rate of skipping appointments. An interesting health talk is going on. One of the peer educators a man who has been on treatment for long and is in a discordant relationship is introduced and straight away begins his talk. He talks about why it is important not to spread the virus and talks about saving human nature. *Akawuka sikalungi nakamu* (the HIV virus is not good at all) and we need to work hard to help those who have not contracted it. He says he is open about his status. (He is big and good looking). He repeats the usual instructions, swallowing medicine on time, no alcohol, eat well (leafy greens and fruits as common as guarvas are good for you). Avoid stressful situations. ‘With stress your CD4 will reduce, weight remain stagnant and the medicine will not work for you.’ He talks about interesting things. He brings up the issue of having sex all the time. He cautions, ‘Don’t have frequent sex, you don’t have that much energy. Twice a week is enough, not more than that. Those of you who are discordant, don’t hide use condoms. I and my wife use condoms, you also do the same. Hiding is one of the sources of distress and secrecy. Some of you hide your medicines very far, and people at home don’t know you’re taking medicine. It gets cold, you’re taking spoilt medicine!!’

At the triage I met Mayia who looked sick, she accepted to talk to me after the clinic. She had been blasted by the triage nurse for smelling booze, but she quickly said she sells it and it had poured on her. I later smelled it in her breath as we talked and she actually said sometimes she drinks because of distress. I later requested her for aninterview, we chatted for a very longtime. It was quite hard for her to understand my question on resources. What was on her mind was ‘*buyambi*’ (support). She kept bringing this up. I had to probe to get what I wanted. She is quite knowledgeable about HIV related issues and positive living. She told me Septin was to prevent illnesses (*bilwadde)* e.g musujja, lubuto. These used to disturb her a lot but she is now relatively better. She looks sick but is smart in a white gomesi and sash, permed hair and a bangle, shoes.

Later, I converse with a records assistant and two counselors about stigma. A counselor shares a case with me of a woman who came for PMTCT today and was shocked at the length of the line. ‘You mean this is how I am going to be lining up?’ she asked. The counselor tells her she will be helped from inside because she is on PMTCT but after birth, she will have to join the long line. She exclaims rhetorically, *wano nasobolawo*? (Will I manage this place?) You see that man over there *(she points at an old man seated a distance away*). He is my paternal grandparent and the other one there my uncle (paternal). All those guys get medicine here. I can’t manage this place. Even my uncle and grand parent to know that I am HIV +ve. Some things are supposed to be kept to oneself.’

Later I join a counselor orienting one patient who is beginning ART. She is smart and quickly understands the instructions. He talks about side effects using those for chloroquine and quinine as examples. He mentions rash, diarrhea, vomiting, nausea, jaundice (enkaka), sores in the mouth, headache, scary dreams. He tells her to drink lots of water and links it to a good skin. She asks whether taking just one bottle of beer is a problem. She has been taking a beer brand called bell whilst on Septrin as a way of fitting in with her friends. ‘Your friends can offer you beer, how do you refuse or to ask for soda when others are taking beer? I thought *waragi* (a local spirit) was the problem, she said.

In response, the counselor says that the choice is hers. But says booze obstructs the absorption of the drugs, increases toxicity of the liver and impairs its functioning and may make her forget to take the medication on time. She quickly says, she would stop taking alcohol.

The side effects he considers very serious and the person should straight away come to the hospital are; diarrhoea, sores, rashes and swelling. He says that the others can be treated from a nearby health facility or one can buy medicine from a drug shop and manage them.

**4^th^ December 2015**

I arrived at about 8:30a.m, by passed a nurse at the entrance of the treatment centre. We smiled at each other and I screamed, ‘I have caught you late’. She just smiled back and continued walking to the HIV clinic. The place was quite busy, many movements allover the place. Pregnant women in labour strolling around the compound. Mothers moving with babies to the immunization and antenatal section. The OPD was quite full too. People waited patiently on the benches. At the HIV clinic, people were already seated. The triage had began, but the doctors were not yet there. A nurse was organizing medicines on the trolley. I went into the dispensing room and found the Septrine packed. A counselor came in and told me she was going to orient a gentleman about ART. Most of the rooms were still closed but we sat in a small room called a store, there were no chairs but we used wheel chairs and the session went on. The gentleman was going to begin ART. He had come with a CD4 test count of 79. He told me he had tested last year in around June (his nephew a doctor – in –charge of a health centre III in an island had tested him). He is from the city but decided to come all the way to this facility because his nephew had indicated it would not disturb him and he knew people here. The decision to test start ARVs and do CD4 and enroll on ART were taken by his nephew. ‘He is my planner, he is the one who has brought me here to start medicine’, he said. When his nephew brought him he handed him to a clinical officer who often works in the HIV clinic, but is at the general OPD the rest of the week. It was a Friday he was not scheduled to work at the HIV clinic, but he brought his patient together with his nephew and handed them over to this counselor and told her to counsel him in preparation for ARVs, open him a file and return him to the OPD for him to review. The counselor quickly organized to counsel him and kept describing him as the clinical officers’ person. She informed him about the medicine, its rules and rationale for taking it, feeding, the need to adhere and travel with it, coming back for review. In the process, she followed the guidelines on the chart provided by the Uganada ministry of health to talk about each and everything. The side effects were however not explained in-depth. He said he had no questions and that he and his wife were aware that they had to use condoms because she was negative.

During the session his nephew brought him a book, he thanked him (smilingly) then he said bye. He was going to his duty station. After the talk, he was registered and a file opened for him. He was then taken to the clinical officer who prescribed TDF / 3TC/EFV for him and asked that he does liver and kidney functioning tests. The counselor brought the book to the dispensing room and identified it as belonging the clinical officer’s relation. We had not strated dispensing medicines but we immediately got his and put them aside. He later came for them. The nurse politely explained the instructions to him and shared with me that she was going to tell him to swallow all the medicines at night to make his life easier. The counselor followed up later to ensure that he had been worked on.

**21^st^ December 2015**

Today I helped out at the triage briefly before being asked to assit a counselor who worked on mothers under the PMCT arrangement. Many of them still had very little babies. I was told that they would eventually refer them back to the general HIV clinic, when their babies were of age. Out of curiosity I asked each mother I worked on whether her husband was also enrolled on ART. It struck me that several of the women were not sure of their husbands’ status or whether they were on treatment because they had not disclosed. One woman categorically said, ‘*yamanyi ebibye, nze ndikulwange* (each of us is on their own).’ When I inquired how they manage to keep their status discreet, I found that several of them used often costly strategies like travelling very far for treatment and hiding their medicines in all sorts of places. One 23 year old housewife, call her Sarah said that she kept her medicines with a sister who was a known HIV patient in the family. This sister had advised her to do so when she confided in her. But because the sister lives a considerable distance away from Sarah and her husband, she has to part with 2000/= everyday on transport to and from her sister’s home to swallow the single dose regimen (TDF/3TC/EFV) she is on every evening. Asked if she didn’t find it difficult to leave when her husband was around, she said that in such cases she gets excuses. ‘I can for instance tell him that I need to pick something from my sister or that my sister is not well and needs my help’, she said. She observed that her sister was particularly supportive and told of an incident when her husband returned unexpectedly and found her getting ready to take her tins of ARVs she had picked from the treatment centre earlier in the day to her. He was bitter when he saw the tins and asked her what she was doing with medicines that looked like ARVs, but she remained composed and told him that they were her sisters’ medicines and that her sister had asked her to collect them on her behalf because she was ill. He called her sister immediately to verify, then she confirmed the narrative. One of the mothers in a discordant relationship however noted that she had been tested together with her husband and that he was very supportive. I noticed that she travelled all the way from Kampala City, probably as away of maintaining anonymity.

In the afternoon I rushed to visit Rose. On my way out I identified a client from a distant place. I asked her why she decided to come this far. Her response was, ‘this is where I want’. When I probed, she told me, I don’t want to be gossiped about. They gossip about you everywhere you go’. I asked if she knew any people in her town on ARVs and her response was yes. Then she said that they are always gossiped about. I gave her lift upto a trading centre where she was expected to pick a taxi then I proceeded to visit Rose.

The journey to Rose’s home is smooth without much traffic. It is easy to trace it, the road is not potty but very dusty. I felt sorry for Rose as I drove through the dusty road coupled with the violent heat. I remembered she had braved all these hassles alone on Friday last week in her very weak state. She later told me she was feeling very dizzy that day. The reason she comes alone is that the treatment supporter (her niece, a daughter to the brother she follows) is not around. She is at school. He mother is well known. I simply asked a *bodaboda rider* at the stage then he immediately knew who I was talking about. He took me there and charged me three thousand. Rose told me they charge them only 1000/= for the trip to town, I was just exploited. She was delighted to see me, she wore a broad smile as I parked and she realized I was the one she was anxiously waiting for me in the shade, her phone beside her as she expected me to call.

She extended her ‘frail’ hand to greet me as I walked towards the verandah where she was sitting. She invited me inside the house (a semi permanent house with a quite suitable sitting room well furnished with sofa sets and several pictures). She quickly gets into the house and gets a cleaner mat, where she directs me to sit.

I suggested we sit outside.

I sit facing her. She had a bowl of food and was about to begin eating. She called one of the girls to clear the food but I suggested she eats while we talk. Her big brother came to greet me. She introduced me as a student who helped at the clinic, the guy greeted me again. She started eating but kept on thanking me for coming. She kept on calling for someone to eat. He later emerged and was introduced as her other brother who was almost dead a few months ago. She insisted that he is now fine, even though he looked evidently weak. She tells him come and eat, he checks the food (sweet potatoes and tomato sauce) sits a bit and goes away. She tells me ‘*anzijanjaba nga nange bwenamujanjaba’* (we care for each other). I always look out for him to eat.’ She asks me to save for her my number, which I do. I ask about her family, mum, siblings, children. She tells me this is her home where she grew up. She stopped in P.4 when her father died and went to the city to be a house girl. She served as one for six years, and then she got a man. She said she eloped with a man. He took her to Mpigi, where she produced 2 children. He died in an accident whilst driving a taxi which he did for a living then Rose returned to her birth place. There she started a business of selling bananas in the nearby trading centre. She found a new man then they got two children. The youngest was conceived when she was on treatment. She started getting wounds and eventually herpes zoster, then her mother sdvised her to test for HIV. She was found positive, but at that time her treatment centre just had Septrin, which must have been around 2005 to 2007 and eventually put on ARVs. Last year her health started deteriorating. She got rashes and sores in the mouth. The health workers changed her to a second line regimen, the last time she visited but her condition is yet to improve. Asked how she ended up living with her mother, she said that when she was critically ill her business started collapsing. Her husband was care taking but not giving her the money to cater for her treatment. She was finding it hard to find food, transport and medication. But she feared to tell her mother and siblings. She said that she didn’t want to bother them. Besides, they had warned her against marrying her current husband but she had insisted. When she heard that her daughter was ill, her mother went to visit her. Seeing her condition, she was concerned and told her to ask her in case she needed anything. ‘She came to visit and found me in a terrible shape. I was thin and basically struggling to survive. When I shared my challenges with getting money for transport to come there [treatment centre], food and buying medicines she got concerned and told me to inform her when I need anything.’ She started providing transport, money and food. Rose said that she would use money from her banana business to meet her needs, but when she didn’t have would inform her mother then she would provide. She said that she left her husband last year because he didn’t care about her at all. It was difficult to get money from him for treatment. She decided to pack her bags and return to her mother’s house. They even had a row over assets, chickens, goats, and a cow. Rose had to compensate the husband for his input into their rearing especially during her illness.

I observed that Rose’s mum seems to be the strong hold in the family. She takes care of her grandchildren as well. These are in turn deployed to help her children. One a boy helps Ernest her brother who is also HIV positive and recuperating from home ill. He knows all the corners. Ernest tells me, he is managed to befriend the counselors especially by being vocal. He is now well known and is always helped to jump the queue. Rose and her family are Anglicans and are quite active in church. The priest and the congregation always pass by to say hi to her. Once in a while, the mother engages a bodaboda to take her to church. She needs about 10,000/- every visit. She tells me her appetite is good and I witness her eating lots of sweet potatoes. ‘I eat everything,’ she tells me.

**15^th^ January, 2016**

This Friday was busy as usual. The foyer, verandah and corridors were all full of people. The activities had begun in full swing when the expert patient at the triage called Ken (another expert patient) to receive a particular client. I looked closely. I could see that she was very thin and frail. Ali took her by the hand and took her to see the doctor immediately. After about 15 minutes she was sent to the triage to weigh herself. Ali followed her shortly and inquired how much she had weighed. The expert client says 20 kilogrammes. Ken joked that she was doing well, he had expected her to be 15kgs then both of them laughed. He took her by the hand once again and led her back to the doctor’s room. About 15 minutes later they were back, this time with the clinical officer who was holding her book and what seemed to be an x-ray. The clinical officer informed the nurse that Alisaba was going to be admitted and that they suspected that she had T.B. Ken was asked to go to the T.B clinic and invite the person incharge to come and examine her. She was meanwhile taken to the ward, where I followed her. I introduced myself and conveyed my sympathy about her illness. She told me she was struggling to keep alive in vain and that she had swallowed all sorts of medicines including Septrin and ARVs. Shortly after Ken returned with the T.B incharge and the clinical officer. The T.B incharge started questioning Alisaba. He asked her if she usually sweats and gets cold at night to which she replied yes. He then asked her if she had sputum, then she said no and explained that health workers at a big public hospital where she had been taken in a critical condition had failed to do the T.B test due to her lack of sputum and asked her to do an x-ray instead. She also said that sometimes she finds difficulty in breathing. The clinical officer said that the x-ray was not clear if she had T.B. She added that there were several other tests they could conduct but they lacked time because the samples had to be taken to to a big hospital for examination. The two health workers agreed that Alisaba should be immediately started on T.B treatment as they determine the next step. To assess her readiness, the T.B incharge asked Alisaba if she would adhere well to the medicine. In response she said that she had no choice but to adhere because she wanted to live. She explained that she is a good patient who always empties her tins of ARVS before returning for refill. ‘I would be dead by now if I wasn’t taking my drugs well,’ she said. The T.B incharge asked her where she lived and whether she had a telephone contact in case they wanted to follow her up. Alisaba said that she didn’t have a phone and explained that she sold everything including the phone to raise money for treatment. ‘The problem is that I have been going to public health facilities but there are no drugs. We had to buy all the drugs and then pay for the x-ray. It has been hard, I had to sell everything,’ she said. The T.B incharge said that it was okay and informed her that he was going to start her on T.B treatment. He said that he would return later to orient her about the treatment to which she replied okay. Ken teased Alisaba that he was supposed to marry her, but now he can’t because she is like a sister to him. The two of them giggled together and started talking about a party that Ken and his colleagues had organized for PLHIV at the end of the year and how Alisaba had enjoyed it. He then went and and called the nurse incharge of the ward to attend to her.

When the nurse looked at the prescription, she said that none of the drugs was available. She told Ken that they had to buy them, then she left. Ken took the book and walked out of the ward. 15 minutes later he was back with a black polythene full of medicines. He handed them to Alisaba and told her that he had bought all the medicines she needed. The nurse returned to put her on drip. She screamed with pain as they pricked her hand. I urged her to look away and joked that she should be used to injections by now, to which she replied that it was difficult to ever get used to being pricked. She implored the nurse to handle her with care and said her whole body was paining. Her pleas were echoed by Ken. Ken and nurse left when the drip started running. Ken said he was going to buy her food. She reminded him not to forget her 10 year old son that had escorted her. He was playing outside. After Ken and the nurse left, Alisaba told me that she was now hopeful that she would survive. ‘I had been taken to the big Hospital when I was almost a corpse, but with this treatment I think I will recover,’ she said. I asked her to tell me about her illness experience.

She said that she was strong and running a restaurant in when she suddenly fell ill. She was already on ARVs having been detected with HIV during her fourth pregnancy. She had broken up with the father of the children shortly after. She had met and started living with a new man when her health deteriorated. It started like fever, but soon her whole body was weak and full of heat. Time came when she could no longer work and relocated to the village to her mother’s house with her four children. They took her from one clinic and hospital to another but the condition was not improving. Eventually they tried herbalists and that is when she got some relief. She was okay for sometime and back to doing simple garden work but fell critically ill again. She said that she was taken to the big hospital when she was almost a corpse and has been on and off for 3 years. ‘The worst thing is that I don’t have much support. I have been struggling almost alone and sold everything, *musawo* everything. The only peole who have stood by me are my mother and one brother but both of them are lame. My brother tries to look for money to help me, but he is a mere petty trader and has a family to fend for. Sometimes he fails to meet my needs. Like tea last time I was here, they told me to buy some medicine to treat sores in my mouth, but it was very expansive. My brother could only buy a few tablets, I did not finish the dose.’ I asked if she had no other siblings, then she said that they were there. She had sisters but they didn’t care that much and that none of them had bothered to visit her during her three years of illness. She said that she had decided to ignore them. I asked if any of them had been informed that she was currently admitted, then she said that most likely they didn’t know, because she had not informed them. ‘If someone doesn’t care about you, then you have to let them be. The world is so changed, you beg them and get tired. So I left them to be.’ She added that even her friends abandoned her when her condition deteriorated. ‘How about your new husband?’ I asked. She laughed and said that he was supportive at first. He even moved with her and the children to the village. At the time she was still looking relatively physically healthy but when she started losing weight and became thin he got scared that she could have HIV and left. He asked her, ‘Alisaba, isn’t that HIV?’ then she denied but he took off and has never come back. She laughed and said that he even forgot his son from an earlier relationship they had been living with, with her and has never come back to check on him. She said that it was now two years without any communication from him.

(Ken comes back with two plates of food for her and the child and said he would return later. She starts wondering where the kid is and complains that he is stupid and stubborn. He may get lost. I offer to go and bring him. I found him sitting in an isolated part of the clinic and playing with sand and told him that his mother was calling him to eat food, then he responded. The boy sat on the opposite bed and started eating whilst I continued sitting beside Alisaba on her bed. She had laid the thin cloth she had wrapped around her waist (*leesu*) on the bed because she had come with no bed linen. She didn’t know she would be admitted. We continued talking).

I asked what her mother did for a living, then she said that she was a peasant farmer. I asked if she had a big piece of land to cultivate then she said that it was very small. She explianed that the place they were staying wasn’t theirs. They were living on land for her maternal relatives. It had been given to her mother to stay when she separated from her dad and returned to her birth place with her four children. I asked about her father and whether he was supportive then she said that they were not in touch. He had remained in theier initial place when her mother left and since then their relationship has been strained. I asked about her relationship with counselor Ken. She said that she had met him here at the health facility and that he always helps patients who are ill to get treatment faster. ‘You see he is good, when you come here when you are sick, he really cares for you and comes and checks on you.’ She added that he had visited her at home several times. She said that this time she had called and informed him that she was not okay and her brother had no money to take her to hospital. He told her to come and that he would be waiting for her. I inquired where she got his phone number from then she said that he shares it openly at the triage and tells patients to call him incase they face any problems. As if to illustrate how good he was she told me that he has given her transport to take her back home on several occassions when she didn’t have enough money to travel back home on clinic days. ‘I get my brother’s phone and call him. Counselor, I am supposed to return on such a day but I only have money to come. Will you help me get transport back so that I come? He always tells me come and gets me transport back home’, she said. I inquired how she managed to come in her weak state? She said that it was abodaboda rider who is their neighbor who rode her up to the main road, where she boarded a public taxi. ‘I told him I was going to hospital, then he picked me from home. And you know what he never charged me any money. When we reached the highway I reached my bag to pay him then he said leave the money, travel safely.’ She added that several riders have been good to her throughout the illness. She said that she rarely pays the 2000/= from the village to the highway, because they excuse her, and that this had helped her to save the little money she gets from her brother and wellwishers for tough times. She observed that she had used the money she keeps for transport this time round because her brother was yet to mobilize her transport to the health facility. She explained that she had returned earlier than she had been scheducled and so her brother was not yet ready.

I reminded her to eat her food then she said that she was scared of vomiting. She noted that she had a big appetite but vomits whatever she eats. ‘But do you get enough food to eat?’ I asked. They said that they didn’t have adequate food as a household but the neighbours are supportive. She said that they sometimes provide food when they have, whilst others give her money, and this support helps her to survive. She mentioned a particular lady from her Pentecostal church who offered her a daily supply of a cup of undiluted milk, when she shared her problems with the congregation. She said that she has been receiving the milk for the last 3 months. I observed that she said her mother was disabled, so I inquired who tills their land. She said that it was her mother, but the problem was that she was almost always caring for her in the hospital and so seasons usually passby without them cultivating anything.

The T.B incharge and Ken returned to give her the T.B drugs. The incharge cautioned her that she should make sure she takes the drugs everyday. Alisaba responded that she had no problem with taking the drugs but getting food was a problem. Ken observed that he had realized that they didn’t seem to have food, when he visited. ‘I didn’t see any food in their home,’ he said. Ken challenged me to find a way of helping Alisaba address the food scarcity. I laughed and told him that I would teach him social work so that he becomes a more effective resource mobiliser and problem solver. He laughed it off as he walked out and told Alisaba to engage me more about it. We continued talking. Alisaba told me that she was big and that she has a good body but the heat in the body keeps disturbing her. She said that she keeps buying medicines prescribed here from clinics in bits because she has no money. She said that she may get for 500/- then 1000/- as and when money flows in. when I inquired how much the medicines she was talking about were, she said that they were very expensive and that sometimes she used aloevera when she had no money for the next dose. She had shared that she thought the vomiting was the one keeping her in and out of hospital when I noticed that the drip was almost finished. I rushed to call the nurse. The nurse stopped it and said that they were going to put her on another one. It was late in the afternoon; I thought she needed to rest. I said my good byes, gave her 10000/- for buying a drink and promised to visit her together with Ken in the near future.

**19^th^ January, 2016**

I helped at the treatment centre before proceeding to to interview the mother of one patient Mukasa. I arrived early at around 7:00am. The sun was already shining brightly. I found several patients seated on the verandah outside the clinic. The health workers were yet to arrive and almost all doors to the key rooms, dispensing, store were still locked. At around 7.30am a counselor/expert client arrived and opened for me the dispensing room, where I sat for a while whilst observing what was going on outside through the window. Whilst there a gentleman- call him John walked to the window and asked to talk to me. He whispered that he wanted me to receive his book privately and explained that he didn’t want to be seen at the triage, some of his wife’s friends could be there. I inquired how he manages everyday, then he said that he always talks to one of the basawo to receive his book for him. When I accepted he reached for his leg, pulled a book out of his socks and handed it to me. I engaged him about how he managed to wade off the wife’s suspicion. He told me that he was very careful and that he kept his medicines with a workmate (fellow bodaboda rider) who was his friend. He said he went there to swallow his morning dose on his way to work and took the evening one on his way home from work. He observed that his wife was very hard working and he didn’t want to lose her. But she is very tough and was always warning her about promiscuity. ‘I know she will leave, I just know it,’ he said. ‘That woman is very tough.’ I had to take him to a consultation room for his weight, blood pressure to be taken before lining him up to see the doctor. He came to thank me before he left.

By 10:00 a.m the clinic was a hive of activity. The triage, corridors and waiting area were packed with patients. The sun was shining brightly and had by now affected part of the clinic compound where patients who could not find seatssat as they waited for their turn. Th clinical officers, counsellors were all actively seeing clients and several of them were waiting for medicine outside the dispensing room where I and a nurse were serving them. In the midst of parking medicines for one client an elderly man probably in his late 60s –call him Mzee, appeared at the dispensing window and dumped a black polythene bag containing his newly initiated ARVs there, saying, ‘*eddagala lyamwe liryo* (Here is your medicine). I do not want it anymore. I have taken alcohol all of my life, but I have never felt this drunk. How can you sleep and wake up drunk for a whole week? I stopped taking this medicine a week ago but I still feel dizzy. Take it I am no longer interested.’ The nurse was dumb founded and stared at him for a while before asking one of the counselors to talk to him and convince him to continue with the treatment. The counselor emerged after about 45 minutes of engaging him and said that it was difficult to convince Mzee that he would eventually feel better. She said that she cited several cases of patients who had experienced similar side effects but recovered to convince him.

The clinic ended at about 4:00pm. I left the staff clearing up the medicines, records and files then I excused myself to go and visit Mukasa. The direction his mother had given me was not difficult to follow and within a few minutes I had found her.

**22nd January 2016**

I started off in the morning to the treatment centre. The drive was smooth and by 7:30 am I was at the health facility. It was deserted. A few people in the newly constructed waiting area of the maternity ward. Surprisingly too a handful at the HIV clinic. I greeted those seated outside and those standing in the walk way. I tried one door after the other they were all locked, save from one consultation room in which a strange youngman was standing. I greeted him and asked him where these people were. He told me they had not come and walked off in to the wards in the opposite direction. I later learnt that he is an intern. I decided to sit on the bench and wrote my notes. A few minutes later, a young girl accosted me and at first failed to explain what she wanted. Then later she told me that she was a nursing student who was told to come for internship. I told her to sit and wait, they were not yet here. We chatted a bit about her career as I continued to write my notes. The student went to see someone in another unit and left me seated with a group of female patients, who always seem to be the first to arrive at the clinics. I started conversing with them and asking them how long they have been on treatment and their experiences thriving on it. I heard several stories and mobilising transport emerged as a key issue for several of these clients. A retired teacher said she had been very ill for about three years but had now improved. She said that she stays with her parents who give her transport to come here. ‘Even, my daughters one works as a mobile money agent. My husband is a teacher who also helps he is now very fat, I think he started the drugs earlier and didn’t tell me. I have had the disease for 3 years but had not known until I got herpes zoster on the arm when I came to the health facility I was tested and I was positive. I would vomit everything I would eat, but now I like to eat posho very much and I love to eat a lot,’ she said. She said she had been overworking whilst teaching but her daughter told her to stop working. Getting medicine when she was working was difficult but she engaged one of the counsellors to pick it for her. ‘When I was still working I used to call counselor naka and she would pick it for me and I would go to her place on Friday or Saturday to pick it. So I would survive on the extra given due to the demands of private schools. But now because I am very far I have to come for it myself.’

Another woman call her Eve said she lives in a city suburb and that she came all the way to this facility to avoid being gossiped about, but sometimes she got caught up due to work commitments and failed to make it to the clinic on time. She said that in such cases she called one counselor and asked her to pick her drugs, then she went to her home and picked them from there later in the day. She said that she had only disclosed to her siblings and mother and they were very supportive. ‘My brothers and mum call me to find out whether I have taken my medicine and if I have forgotten then I take. They also tell me to eat this or the other and if I tell them like I have no money then they send on my mobile money. I take milk at least twice a week. So that is how they help me.’

Another woman probably in her mid 20s shared that she sometimes finds difficulty mobilizing money for transport but her mother-in-law to whom she is very close and also helps, gives her when she asks. She added that sometimes she walks half the distance to cut the costs. A middle aged woman from a neighbouring district said that she spends between 14000-15000/= per trip and that she was increasingly finding it difficult to mobilize the money. She said that at first she had money from her savings where she used to work in the islands. But when she fell critically ill, her parents picked her from there and took her to their home. She was now living with her parents, was feeling better but was yet to find a job. She said that her parents were unable to support her financially. I encouraged her to ask for a transfer to facility near home but she seemed hesistant, perhaps due to stigma. A woman in a discordant relationship shared that she had no problem with treatment and that her husband was supportive. ‘I don’t face any problems with the ARVs and my husband is supportive he gives me transport to come here and back home. We came with him here 3 times to test and he is negative so he is supportive and he reminds of the time when I should take my drugs,’ she explained. Another woman –call her Cathy shared that her mother was supportive since she learnt about her HIV status. She said that she was concerned that she is not eating well, so she sends her food from the village on a weekly basis to ensure that she gets no problems with feeding.

From the stories I observed that a couple of the women had not disclosed to close relations including those that were supportive. I asked one of them how she managed. She said, I just tell them that I have a problem please send me a ten thousand or twenty and you survive like that.’ A teacher in a private school with a history of heart disease said that she managed to get permission to come for refill because she told her bosses that she comes for regular check-up for her heart disease.

We were interrupted by the arrival of one of the expert patients and counselor. She greeted me and I joked, that I had got her late. She was delighted to see me. We hugged and then she asked me to help her sort the files. The counselors started arriving one by one. The drugs were assembled on to a trolley but the nurses/clinical officers were nowhere to be seen. The first nurse arrived a few minutes after 9:00 and started giving instructions and deploying intern students. I commented that the clinic had began late, she told me that health talks had to first be given. I listened to some of them. The patients were informed of the new changes. They were not allowed to crowd the narrow corridor, only 5 people at a time. They were supposed to carry the balances, even those in sackets removed the tins. They had to be patient and disciplined if they wanted a good service. No file would be moved around. Some patients complained that the people, who come first, leave last. Their details and counting were to be done at the triage. I personally did not like the idea of counting drugs at the triage as it was too intrusive. One patient was reprimanded for removing her medicines from the tins and keeping them in sachets. She looked uncomfortable as other patients stared at her. Soon the clinic began. I learnt that the first line regimen for children had been changed from CBV/N to Abicavir Sulfate/lamivudine/EFV because the former drug was associated with aneamia. Those already on it were going to remain, but no new people are going to be started on it. So much happened as I helped with the counting of some drugs. One guy an accountant with a popular radio station, walked to the window and exchanged greetings with the nurse. The nurse said he was lost and asked him if he had just come for a visit. He said he had come for refill and proceeded to the file room to obtain his file. He was clean, eloquent and confident. He stormed in a few minutes later and pulled a clean note book out of his bag. Before he returned from the file room one counselor plotted to get money from him. She asked for soda for all of us but he laughed it off. They teased him about getting them a radio to listen so that he could send them greetings. The nurse received his book and as he gave it to her a counselor commended him for keeping it as clean as he looks. The nurse looked through the book and asked ‘how many months?’ Two? He replied 3. She asked, ‘Are you going far?’ then he noted, ‘I am here and there. Coming here wastes a lot of time. Today is gone, I won’t work.’ The counselor urged him to go and work because they had attended to him very fast. He packed his medicines for 3 months and noted he had not tested CD4 in a long time. The nurse advised him to come another day for only that if he was worried about the time. He was told that the CD4 people work on only clinic days when he indicated that he would come on Saturday.

Another patient came with a loaded bag. He squatted and talked to a nurse. He handed her his book and noted that he was supposed to come next week but had safari and needed 3 months. He wanted another favour, the nurse to keep the medicine for him as he went somewhere. He also gave her some food which he had brought, in a bag. She thanked him for digging and *kwetika* (the gesture). He left the bag behind and left. Another woman lobbied for 3 months, arguing that she was going on safari. The nurse complied and tried to explain that she should swallow one particular tin first because it was expiring in (March). So much was going on. I realized the counselors are very free with the nurse. Too much whispers in the ear and the patients too were free with her. One counselor pulled me aside and said she wanted to tell me something. She told me she had got some money and bought bricks and sand. She needed cement about 10-20 bags to construct her house. I promised to help and told her to inform me when she is ready to start. I left the clinic at about 11:00a.m to visit Mutetsi.

**12^th^ February 2016**

I arrived at the treatment centre early and decided to keep in the car to eat something. I also called Namatovu to ascertain if she was already around. I had asked her for an interview on an earlier visit but she had said she had to rush back to work and offered to talk to me on her return date which was today if possible. She did not pick. I later went to the clinic. There were over 20 people already at 7:30 a.m. an expert client had already arrived and started looking for files. I bumped into Allen. I was surprised that she was already here when the pregnancy is almost due. I joked you are too early with your big stomach. She responded, do you see all these people here, if I don’t come early musawo, I will just sleep here. Maris the baby girl was not around today. She had left her with a neighbour and friend and simply told her she had to come for ANC. She told me the baby disturbs her a lot. I dropped my bag inside the dispensing room and came outside to try and call Namatovu again. I noticed the corridors were lined up with people. The benches outside were also full. There was already nowhere to sit. Even Allen the pregnant woman was standing. Her phone went through she told me she was at the gate. A few minutes later, she came in rushing. We smiled at each other, exchanged greetings and she moved on to check the register. I noticed she only greeted one of the patients seated on the front bench. One of the people I found already at the clinic. They talked for a while then she proceeded inside. A few minutes later she was back. She told me her file was already found. I suggested we talk while she waits for the clinic to begin. We moved away from the triage/reception and sat on a verandah of one of the latrines adjacent the clinic. We started talking. I learnt that the lady she had greeted was one of her friends she had made at the HIV clinic. At first, they were a big group but some people ended up with other TCA days so they have lost touch. She particularly exchanged numbers with the lady she greeted and Eva the friend who often helped her to collect her medicines when she could not make it. She explained that the arrangement was suggested by the lady when they were seated as a group in the compound and started complaining about the long waiting time. The strategy seemed to be working for her. She came late but was called for triage even before Allen. I was surprised. She told me she was number 5 on the list and I wondered how. Her response was that the lady had registered her. They knew each other’s file number. She had called and informed her that she had to report to school first, but was coming. She should help and register her. Whoever is late in their group often does the same. Sometimes they check on each other. She for instance called Eva to ascertain if she was coming when she realized she was not around. Eva told her she had sent someone. She spotted the man and showed him to me. I asked how she had got to know him. She told me he is one of them. He stays in the same town like Eva. They met here and got to know each other. The interview was often interrupted because she thought they had called her name. She kept running to check. She was anxious to go, telling me her lessons were in the afternoon, but she had a lot of stuff to prepare and tests to mark before then. I was forced to stop the interview after covering only a few questions about how she knew she had HIV and started treatment when her colleague came to inform her that she was next. She said that the doctor was seeing somebody but she was next in the queue. As she sped off to the doctor’s room, I stopped to speak to Allen. She was still in the line despite having come earlier than Namatovu. She was bitter when I commented that she had taken long to be seen. She told me ‘with the voice raised high’ to the dismay of her colleagues seated next to her, it seems some people are registered by those who come early. I see the people who have just come being worked on. It is difficult to control the flow of books on a first come first served basis here. The books are distributed to different clinical officers and sometimes nurses whose speed varies. One intern spends lots of time on particular patients another clinical officer prescribes too many medicines so sometimes it’s difficult to keep the order. Allen complained that she had not received any jerrycan or mosquito net for the years she has been on this programme. ‘Today, I am not leaving until I receive them,’ she said. I told her people had received on Tuesday. He colleagues told her the packages are distributed on only Tuesdays. One of them told me the jerrycans are commonly abandoned at the clinic. People only remove the mosquito net. She told me she can’t take the jerrycan anywhere either. I joked that she should take it and hide it in the house. Allen also added that she would tell those asking where she gets drinking water from that it is in a pot. She noted I have no house, I am in a rented single room (*muzigo*). This suggested fear of stigma. I excused myself to go and help in the dispensing room but it was still empty. Medicines were loaded on the trolley but there was no one. Namatovu and several others who had been seen by the clinical officer were standing anxiously at the window. Namatovu told me, the staff were in a meeting. She was going to leave the book behind she talked to the man her friend Eva had sent to pick her drugs, ‘receive my medicine too. I have to go to school. Give them to Eva, I will come later and pick it.’ In the process the nurse came. I informed her that a teacher needed help to go to school. When Namatovu appeared at the window, the nurse knew her. She greeted her. Namatovu had told me earlier that she sometimes talks to this nurse to help her. She always helps. We prioritized her then she left. But others had to keep waiting. The nurse still had a few things to sort before dispensing the medicines.

I decided to help out at the triage meanwhile. Whilst, there I met Tinka a partially blind patient. He was walking slowly and calculatively without help. He told me he had an untreated cataract but no money to treat it. That he had been asked for 350,000/= for surgery but he was yet to mobilize it. We worked on him and as he waited to be seen I sat with and engaged him about how he was managing. I learnt that he was a migrant from south western Uganda and had come to the city to look for work. He had been working on a farm when his eyes failed and he was no longer actively employed but he used his savings to hire land, farm and sell. That is how he managed to pay rent and generally survive. He told me that when he was constrained his tribe mate he had met while working on the farm helped out. He brought him food and contributed towards his transport. But when he did not have money he said that he talked to his friends the boda-boda riders who brought him to the treatment centre, on credit but sometimes for free. He said that it was difficult not to find one who sympathized with him. Asked if they were aware about his status, he said that they didn’t. But they knew he had an eye problem, so he just told them that he wanted to go the health facility but he had no money. Asked how he managed to know the time for taking the medicines and read the prescriptions yet he was living alone, he said that he relied on the radio to know the time. When it was time he asked his immediate neighbor to help him to sort the medicines. He added that the same neighbor helped him to light the charcoal stove when he wanted to cook.

As I walked to the HIV clinic later in the day, I met nurse Phoebe. She had not recognized me. I greeted her first and told her I had been at baby Nakato’s home. Her health was not bad but she is fed on only water with sugar. Ya, she noted that they have not called her in a long time to inform her that the baby is sick. She said that she took her for an X-ray and bought her a flask. ‘What can we do?’ She asked. I noted the mother (Mutetsi) had plans to leave the man and look for a job. Phoebe told me she had had the plans earlier, but she (Musawo) sabotaged them. She wanted to abandon the child with the Dad and look for work. But I told her the baby would die. She would be given to the grandmother who is not aware of her status and die. I recalled that Mutetsi had told me she wants a job where she can be with her child. ‘I am working for my children’ she had told me. So she had been influenced by Musawo. Musawo told me she promised to get her a job. She has been looking around for a job which requires one to keep a house whether unfinished but she has not found it yet. Besides, she has been a bit disoriented by her daughter’s wedding. I told her I had given them some money to buy milk. She said thank you and reiterated what she had remarked in the beginning. That baby is beautiful. Her sister too, even the father.

I met a new client a taxi driver. I asked where his wife was, then he told me that he had informed her but she was yet to test. I doubted because he left the book behind with the counselor. He even refused the medicine we had packed in a tin. He wanted it in a paper bag. We changed it but explained that it was safer in the tin. The counselor told me several clients leave the books behind. They are afraid to keep them at home. I have seen several stapled or punched and filed with the health facility records. Another patient an elderly woman came to the window and immediately told the nurse it was not her day but she should be helped and reminded the nurse that she was in a hurry. I had seen her on Tuesday. Even then she had been impatient and kept complaining. I noted that her aggressiveness will enable her manage the HIV clinic then the nurse agreed. Allen also stood impatiently in the window, complaining that the people who had come after her were leaving. After so much pressure from her, the nurse decided to pull her book out and served her to ease the tension. Some of these patients are also crafty, one came to the window and told the nurse that the Musawo in the room had told her to be given medicine quickly because she had come early. Of course it was not true and on further pressing she conceded that she was lying. Later at around 2:00p.m, I excused myself to go and visit Alisaba together with two expert patients Ken and Benon.

**14^th^ January 2016-**

I helped at the triage today. We worked on over 100 patients. We had to do several measurements, MUAC, BP, height and weight but also give people lab requests for CD4 and full blood count CDC. Several patients came back earlier than scheduled and said that the medicines had got finished. It seems the policy of giving medicines for the exact number of days that is being implemented poses challenges to the patients. Initially they have been giving patients full tins, and days less than 90 (3 months) to ensure that in case they face any challenges they will have extra to push them for sometime until they return. I was informed that the IPdid not like it so it was changed. They thought it was encouraging laziness and skipping of appointments.

Among the patients was a man call him Brian who came back for treatment after two years. He said he had received a call from the treatment centre informing him that his file was going to be closed. He said that he panicked and decided to come to save the situation. Asked why he had disappeared, he said that he had got discouraged the first day he came and decided not to come back. He narrated that he was living with his girlfriend who was in senior 6 by then and did not want her to know that he was HIV positive because she would have left. He said that when he came here he saw several people he thought he knew and got worried that these could inform the girlfriend. He said he reflected and decided that he would not come back. ‘I said I am on Septrin which I can buy, why should I go back there to expose myself.’ He said that he bought the Septrin for a couple of months, but soon his girlfriend complained that he seemed to be taking too many drugs, then he stopped. He explained that the phone call was like a wake up call. He had started feeling sickly but had not mustered the courage to come until he received the call. We referred him to a counselor to engage him further and before he left he told me that they had asked him to come back the following day with his girlfriend for testing.

**26^th^ February, 2016**

I had plans to go to the treatment centre and then visit Bwire yesterday but failed due to a pounding headache. I managed to do so today. I arrived at the facilityat about 8:30a.m but stopped on my way to buy bread and milk for Bwire’s family. I found the staff in a meeting, the triage was set up but not operational. The waiting area was packed with clients, many of whom were engaged in seemingly interesting discusions, whilst others were glued on the TV screen that hang high up in the waiting area. I decided to interact with a few patients as I waited for the staff meeting to end. I looked around and saw a bench that had only two patients, both of whom were men. I joined them, introduced myself and explained why I was at the facility. They said they were glad to see me. I asked them about their experiences with HIV. The one seated next to me- call him Joshua seemed happy to talk about his experience. He told me that he had been on ARVs for close to 5 years now and was doing well. The first few months were very difficult for him though. He came to the facility after a lengthy illness and when they tested him he was positive. His ‘CD’ (CD4) was only 60 at the time and when they started him on medicine, his condition got worse. *Nasesema, nensesema, wiki emu, bbiri, satu, nga buli kendya kadabuzi. Natuuka nga eddagala ndikoye. Nali njagala lisulaayo.* (I vomited and vomited for one, two, three weeks. I would vomit anything I ate. I reached a point where I had got fed-up with the medicine). I felt a strong urge to throw it away. I was fed-up. I called the counselor, explained my condition to him and inquired if it was okay for me to get off the drugs, at least for a while. The counselor said that getting off the drugs was not a good idea. He told me to continue taking the medicine, but go to the nearest health facility to manage the vomiting. *Munange Yanzizamu amanyi okulemerako, era wayita ekiseera nenterera* (My friend, he encouraged me to persevere and after sometime I got better). He said that now he is very strong and does his businesses normally. He observed that following instructions on feeding had helped him and that fellow patients were not improving because they did not know the right foods to eat. He said that he no longer eats fried food but tries to eat as many leafy vegetables as possible. This revelation sparked interest in his colleague-call him Eric. Eric had been listening quietly as Joshua narrated his story. He suddenly had many questions when Joshua brought up the feeding issue. He said that he had started treatment 2 weeks earlier and was feeling weak, and also worried about what would become of him. His wife was negative, she was supportive and always prepared for her leafy greens but he didn’t like them. He was surprised that Joshua said that they had performed wonders for him. Joshua assured him that they work and went on and on explaining how they should be prepared. Eric was impressed and commented that it was necessary to get patients together to share their experiences as such discussions can help new patients like him to easily cope with the demands of managing HIV. As Eric shared how much he was struggling to accept that he was HIV positive and how worried he was that he may not be able to live long enough to see his dream of becoming a successful farmer come to life, the staff returned from the meeting and distracted all of us. The triage nurse spotted me in the crowd and called me to assist her, then I parted with the two gentlemen. Whilst at the triage, I observed that they continued talking and appeared to exchange phone numbers. I imagined that that initial interaction had sparked off a lifetime bond of friendship between the men.

At the triage I helped with the registration, weighing of patients and pointing out any inconsistencies in the dates of return. Here I met several patients as far as from Kayungai. Some had skipped days, others months, yet others had days ahead. One woman was supposed to return in October, 2015 but she came today. She quickly noted that her son had been admitted in Mulago hospital so she could not keep her appointment. The nurse commented that that is why she looks bad; she has not been adhering well although the lady had indicated that she had asked for medicine in Mulago during that time. Another had gone to visit her mum in the city and failed to return in time because she lacked money for transport. Another young lady had come to pick medicine for her mum, claiming that it had got finished before the next appointment date. The nurses objected and questioned her intentions. They noted that they were perhaps planning to travel and wanted to get all the medicine for the next 3 months. She was turned back. While at the triage, I met another client. Her name Carol, only 24 years old. She had been the first to arrive that day. She had come to talk to the nurse about her missing CD4 results and request for a request form. I commented that she looked very young. What had happened? Her eyes immediately got teary. I requested to talk to her privately after she had finished with the laboratory then she accepted.

When I parted with Carol, later in the day I set out to visit Bwire and family. A few metres outside the gate, I met two of the PLHIV I had chatted with a lot at the clinic. One Tolo 40 years looked good and I complemented her even asked her for the secret. She replied ‘mwe’ (you). I had asked about the husband then she told me she had left him. I stopped and asked her where she was going. It turned out that she was going in the same dierction. I offered her a lift and she was very excited. She put her luggage behind and sat in the co-driver’s seat with me. She thanked me several times indicating ‘omponyeza olugendo (you have made my travel easier).’ Another patient Namujju was at the roadside, we also took her and she was happy. She later called me to inform me she had arrived because we dropped her on the way She said, ‘musawo webale okumpa omukisa ntuse (health worker thank you for giving me luck, I have arrived).’ I promised to visit her and she said she would call me.

As we drove towards Bwire’s home, Tolo shared her life story. She had been married to a policeman and in 2003 caught him with a woman where he had been posted. The woman had a rash all over the body which the hubby called measles. She went to a big public hospital to test and turned out positive. The hubby had apologized and even got herbs for both of them to use arguing that it was expensive to be on ARVs. She tasted the herbs, they were very bitter comparable to the taste of raw aloevera. She was a housewife with three children and cultivated for survival. She fell out with the hubby and decided to leave and go back to her parents rather than die in the man’s house. She was pregnant but did not take things seriously and delivered with TBAs. She suspected that the baby got infected because it died. She tested like 2 times more from different places including a private clinic but the results were the same. One man in the private clinic is known to be HIV positive. He helps those he finds negative. He gave her Septrin which she used to collect monthly. Other patients were also receiving the free Septrin from the man. She was always sickly and worried. Some of her relatives did not treat her well when they learned she was positive. One paternal uncle tried to throw her out of her cousin’s house (his son) and embarrassed her one morning when she found her going to pick medicine in town. She stopped her, called her mother and stopped a passerby. He told the mother to remove her daughter from his son’s house because she was almost dying and even gave her 1000 as a condolence to eat while still alive. Several people pointed at her as ‘*kalina silimu*’(she has HIV). The musawo eventually told her to go to her treatment centre in 2007. Here she was counseled and given Septrin. Her CD4 turned out 97, so she started ARVs. She noted that Seka counseled her and she still calls him her friend. But she also knows counselor Fatuma. They came from the same village. She always lobbys for her to be seen quickly. This treatment centre has treated me well. She says it is better because she does not know many people there compared to nearby facilities. Here, *bakwogerako sagala bandabe* (Here they gossip about you, I don’t want to be seen). At first life on ARVs was tough. A week after starting medicine her stomach started swelling. She and her mother were confused. Her mother suggested that she discontinues the medicine for a while then they observe what happens, but Tolo hesistated and instead suggested that they consult a fellow patient (peer educator) who had once mobilized them to form an association for PLHIV in that area. She noted that he died last year though. She had enrolled in the association and they paid 5000/= per month in the hope of benefitting from food which never came. She paid for three months and gave up. The teachers were growing rich yet they got nothing. Her mother accepted to escort her to the peer educator. They strolled to his home and explained her situation to him. She said that the man looked at her sternly for a while and said that he knew what was wrong with her. He said that I was swallowing medicine without drinking enough water and that ARVs are supposed to be accompanied with a lot of water. He advised her to drink a full cup of water after taking the medicine. When she implemented his advice, she got better within a week. She got no other complications while on medicine but she was changed to line 2 TDF/3TC/ Alluvia because her first combination could not improve her CD4. It had stagnated for months. She is now strong and even digs. Her source of income is digging (*kibimbi*) (although at the clinic, she had told me she runs a canteen). She continued to tell me about her life as we drove. Eventually I dropped her off and I proceeded to Bwire’s home.

**12^th^ June 2016**

I called Bwire to say hello, then he told me his wife was sickly. He said that she had been diagnosed with diabetes a very expensive disease to treat. She had to visit a clinic every two weeks. He had some good news though that the wife to landlord had started sending them Dapsone in tins. This had relieved their resources so they can atleast meet the costs for managing the diabetes. I advised that they should visit the treatment centre about the diabetes then he said that he would consider that.
