# Supplementary material for: Social capital and resilience among people living on antiretroviral therapy in resource-poor Uganda
Source: PLoS One. 2018 Jun 11;13(6):e0197979. doi: 10.1371/journal.pone.0197979 (PMC5995438; doi:10.1371/journal.pone.0197979)
Supplement: S1 File — (DOCX) [file pone.0197979.s002.docx]

**Client ID: 001**

Name**: Matiya (Pseudonym)**

Status: **ART (CBV/N)**

**Section 1: Socio demographic characteristics**

Age: **83 years**

Sex:  **Male**

Highest education level attained: (A certificate holder)

Main Source of livelihood: **Pensioner/ farming**

Household size: **10. He has 35 children in total. . Some of the children he stays with are HIV positive.**

**Resources considered Important for the management of HIV and related OIs at home and how they are mobilised**

**Food:** You have to eat well. This means you should not stay hungry the whole day.

**Probe Qn: Which foods do you eat most in your home?**

In my home we eat rice, matooke, cassava, meat, beans, posho, milk. I do not consider rice food so that one is mainly for the children. My children buy the rice and the posho, which I always stock at home. I do not like posho at all. Neither do i like sweet potatoes, they trigger heart burn. I mainly eat matooke. My home is not by the road side but I depend on bodaboda riders to buy me meat, matooke, beans and virtually everything else i need from the centre. They are my friends. I have the phone numbers of the ones I trust. I just call them then they come. A sick person like me also needs fish. But as you know the lakes were sold, we no longer eat any fish. My children eat well. The attendant takes very good care of them.

It is rare to find my home out of enough food. Some of my children care and buy me food. But I also have my own money. I get a pension of 150,000/= Uganda shillings a month and also have a coffee plantation. In a good season I get a minimum of 1,000,000 Uganda shillings. The market is available; I do not move looking for it. Buyers move through the village looking for coffee.

**Safe water:** this is also important. As you know ‘*amazzi bwebulamu*’ (water is life). Here they always tell us to take safe water. We fetch water from the borehole, boil it and put it in a jerrycan. They gave us jerrycans some time back. But they have taken awhile without giving us any.

**Medicines:** for a sick person like me, medicine is very critical. I take medicines from here, but also buy from clinics. You know I have a problem of constipation. It disturbs me a lot. I have reported here. They always give me some yellow tablets but it is on and off. ‘*Bankubako ne enema mu bu clinic okumpi newaka wange*’ (they forced it out (enema) in clinics near my home). But it came back. I tried using ‘*ekisanda*’ (a local herb). It worked for a while but failed later. Every time I go to the clinic for treatment they give me yellow tablets. I do not allow the medicine from here (ARVs) to get finished. Even the yellow tablets, ‘*nabufula mmere*’ (they are like food to me). The children are given tablets by the helper before they go to school at 6:00 a.m. At that time I am still asleep. I take mine later in the day after taking breakfast. I love tea, mine never gets finished from the flask. It is what I survive on the whole day. Eating food is sometimes a problem because of the constipation.

**Probe Qn: Rank the resources you have mentioned**

All of them are equally important. I cannot say one is more important than the other.

**Resources important for the management of HIV/AIDS at the health facility**

Medicine

Health workers

Diagnostics

These three are triplets. The health worker gives you the medicine and without the medicine the health worker cannot treat. The tests run help the health worker to know what to treat. To me they are all equally important. Every 3 months, I come back here and check my ‘CD’ (CD4) then they tell me where I stand.

**Resources important for the management of HIV/AIDS in the community**

I do not see anything much.

**Probe Qn: Even neighbours, friends and relatives are not important?**

‘*Abekyalo obategera nyo*’ (Do you understand the people of the village a lot?). They are always busy with issues that concern them, for instance gardening. ‘*Omuntu atagudde wansi, tebakitwala nga ekintu ekikulu*’ (Helping a person who is not bedridden is not their priority). They only visit in the event of serious illness.

‘*Entegera yabantu yakyuka nyo. Ntono, tebakyaffa kubanabwe. Towulira bwebetema kukyalo. Owulira amatemu agali munsi, edda galingawo*?’ (People’s reasoning is twisted. They no longer care about others. Haven’t you heard how they butcher each other in the villages? You hear tragedies in the world, were those things there in the past?). Poverty and ‘*obutasomeddeko ddala*’ (total illiteracy) have compounded this problem.

I do not get any support from relatives apart from my children. All my siblings died and their children ‘*banejako*’ (distance themselves from me). They neither visit nor call to check how I am doing. Even those I supported with school fees. They only check on me in the event of severe illness like when I got bedridden. ‘*Obuntu bulamu bwakendera mubantu*’ (feelings of Ubuntu- are limited amongst people these days).

**Probe Qn: Have you heard of any Associations of PLHIV in your area?**

Yes. There was an exhibition. World Vision used to give PLHIV sugar, cooking oil and other things. I did not go. The things they were giving out I already had.

**Which support do you receive from fellow PLHIV?**

Which ones? ‘*Abantu bakyekweka*’ (people are still hiding). There are some from my Village I always find here at the HIV clinic. When I ask them why they are at the clinic, several of them claim they have patients admitted in the wards. The annoying thing is that later their names are read at the triage. People like me who are open do not have to hide. What kind of support can I receive from such people?

**How about your village mates?**

(laughs loudly for about a minute) what do you expect from such people. ‘*Mukulaba okwange nze nina okubawa okusinzira kumbera*’ (the way I see their socio-economic situation, it is me supposed to support them). My neighbours always pass by my home, not really to visit but to eat the food I have prepared. Others come to me looking for work. ‘*Bamanyi nze luwombo lwabwe*’ (they know I am their solution).

**Common Illnesses suffered and resources needed**

Flu and cough. The cough mainly affects me, but it can be triggered by anything, not only HIV. Septrin ‘ *byona abivumula*’ (Septrin heals both cough and flu). The neighbours cough and cough, ‘*ffe ngatuli balamu*’ (when we are fine). I fear malaria most. It can kill. But we sleep in mosquito nets and hardly get it.

I always ensure that basic medicine is available in the house. I stock panadol. I always feel ‘’*nga menyese menyese* (general body weakness). One of my children who is a health worker told me to swallow panadol when I feel weak.

**Problems/adversities**

(Laughs) I don’t have much problems. I told you (touches the rosary) Maria blesses me, she never fails. Like I told you some of my children take good care of me. May be the issue of transport. Transport to and from the health facility is particularly challenging, especially when I have to move with all the children. We use *bodaboda* but they are very expensive. A man living in a village like me cannot sustain it.

But one of my sons who stays in the city always comes to my rescue when he can. Like today, he brought me with that whole team. I call him in advance so that he can plan to be in the village the day before to bring us by 6:00a.m. By the time we got here there were not many people. We managed to avoid queuing for long. When we finish I will call him to come and take us back home. When I am alone or with only one child I take a bodaboda up to the road and then board a taxi. I just call boda boda home. When my son is unable to come, there is a special taxi he hires to bring and wait to take us back home.
