# Supplementary material for: Social capital and resilience among people living on antiretroviral therapy in resource-poor Uganda
Source: PLoS One. 2018 Jun 11;13(6):e0197979. doi: 10.1371/journal.pone.0197979 (PMC5995438; doi:10.1371/journal.pone.0197979)
Supplement: S2 File — (DOCX) [file pone.0197979.s003.docx]

**Client ID: 002**

**Name: Kagoya (Pseudonym)**

Status: **ART (CBV/N)**

**Section 1: Socio demographic characteristics**

Age:  **32 years**

Sex: **Female**

Marital status: **Cohabiting**

Highest education level attained: None

Main Source of livelihood: **She has no source of livelihood. She is a** **House wife**

Ethnicity: **Musoga**

Household size: **6.**

**The bigger part of the semi-structured interview took place at her home. We had talked briefly at the health facility but the interruption was too much. She was being called from time to time to answer questions about the baby she had brought for an HIV test. We agreed that I should visit her home to complete it.**

**Who did you register as your treatment supporter?** Nobody. I told the health workers all my people had died. Whom would I have taken?

**Did you disclose to your husband when you found out your status**? No, I have never told him. He just sees me swallowing medicine, swallowing medicine.

**I clarified, so you mean he does not know why you are swallowing the medicine?** (lowers the voice) No.

**Does he ever ask you what the medicine you swallow is for?** He sees the medicine. Sometimes he quarrels, what is this medicine for? Especially when he is drunk. Is it for family (contraceptives)? I will throw them away. I tell him to leave the medicine alone, that I will report him to the health workers. They always come and persuade him to test, but he just keeps lying that he will test but does not. They just gave up on him. When they come they always ask me if he finally tested, I tell them he refused.

I refocused the interview to explore her journey to the ART programme I asked how she went to the treatment centre She asked, *to get medicine? I t tell him that I am going to the health unit, then I go and get the medicine.*

We were briefly interrupted by the children who were playing nearby. The young baby was playing in muddy water and put soap in her eyes. She had to first rescue the baby for us to continue.

**Commonest illnesses**

**Flu and cough –** particularly for the children

**Fever (omusujja)**- these mainly affect me and the boy on ART. We were interrupted by the children who were fighting for a basin. Kagoya had to get up and mediate. She then showed me one of her girls who had very tiny sores on her thighs. She told me it was measles. The concoction her husband was preparing was particularly meant to treat her and protect the rest from the measles.

**Esther: How do you manage when you fail to mobilise money for transport?** I walk to and fro. When I take the boy, he too walks. When I have to take the young baby, I put her on her back and walk all the way. Sometimes we only have money for one way. We normally use it for the journey to the treatment centre so as to arrive early enough, then we walk back home. I rarely go with the boy. This helps me to save money for transport. I only take the boy when he is sick or when his time for testing CD4 is due. **Esther: You have land around here?** No, but we have several places for digging offered by neighbours and friends. You can’t finish all of it. An old man that owns big chunks of land where we used to rent before coming here told us to cultivate wherever we wanted. We actually can’t till all of it. The energy to cultivate huge chunks is not there. We would be facing problems with obtaining food but at least we have where to cultivate. The only problem is the unfavourable weather. For instance last season we cultivated a substantial amount of cassava and sweet potatoes, but the sun dried almost all of them. Our current landlord also allowed us to cultivate on the portion of his land he was not using.

**Probe Qn: What was your relationship with the old man who gave you land for cultivation. Did you do anything special for him?**

Laughs...No, we didn’t do anything. My husband used to converse a lot with him. We were neighbours. You know he is a cobbler. The old man gave him shoes to mend and when he returned them they conversed as usual. He just asked the old man for some little space to dig but he offered us a much bigger plot. He wanted to add us more, but we declined the offer. We don’t have capacity to use it. We still use his land, but also the one offered by our current landlord. Recently, I was going to dig when a car by passed me. It had a loudspeaker, which was used to advertise the medicine they had. They said they were from Namasuba (a suburb in Kampala) and had medicine for cough, skin rashes and many others. I bought a tin for the skin for 1000/= (0.4 cents). I will show it to you when we finish here.

She diverted a little and told me about one of her colleagues at church who had offered her family 2 cups (1 litre) of milk every day. **Esther: What motivated them to give you the offer?** The spirit. In Pentecostal churches, one has to give as directed by God. If your heart tells you to give out the beautiful thing you are wearing to a particular person, you have to comply. Even if it means taking off the beautiful dress you are wearing and give it to another person. This sister just got touched by God to give us the offer. We have been getting the milk for the last 2 years. People here always ask where we get the money to buy milk on a daily basis. The pastor’s wife told me to be discreet about such things.

But whenever the boy goes to pick the milk, people point fingers at him all the way, saying see a person with HIV, see how it looks. The boy returns and tells me, mother as they abuse me that I have HIV. I always allow him to go and watch films. When he asks me I give him money so that he does not get bored here and get into deep thoughts. But sometimes people start pushing him out, telling him to take his slim outside. One day he was in the *kibanda* (a makeshift shelter where films are watched by the public at a fee), then one young boy fixed hands in his pockets. He asked him what he wanted from his pockets, then the other person said that he was looking for money for him to buy medicine for slim so that you can treat yourself. Fellow children hurl insults at him, offering to give him money to treat himself. I always tell him to just ignore such talk and leave it to God. Yet when he goes to the films, it helps him pass time and relieve his thoughts. You would rather get many diseases but not one related to the skin. [Her voice shrieked as she narrated the ordeal of her son].

**Esther: Does he still go to films?** Yes. His fellow children always hurl insults at him wherever he goes. But he has learnt to ignore them. He has nothing to do. You may have this illness when the skins are fine. We do not have any problem, apart from these skins, they are our biggest problem. Some time back he used to be down with fever all the time. He would vomit a lot, but these days he only gets fever when the rash develops blisters.

**Esther: Is there any other particular things you use to manage the blisters from home?** We use warm salty water for bathing to treat the wounds. It is a remedy I learnt from medical workers. I do not know if it works, but I give him warm salty water to bathe daily. **Don’t your friends in this village give you advice on how to improve your skin?**

I do not go to people’s homes to converse.

**Esther: You always keep at home?** I am always here. When you see me moving away from here then I am going to a health facility a garden or church. But saying I am going to another person’s home to converse, aaa...

**You do not have any friends in this neighbourhood?**

No

**Even in church?**

Apart from the church only. There I have some friends. The pastor’s wife is my very good friend. Sometimes when I do not have money to facilitate me to go for refill, I tell her, my friend I do not have money to go for refill, she gives me. I tell her about all my problems.

**How did the pastor’s wife become your friend?**

We met in church.

**How did she get to know you, did you seek counselling?**

Uuummh, we saw each other in church. Now she is the friend I to talk to.

**You confide in her everything about you?** uum...

**and she does not back bite,** uum, she does not backbite

**and helps you...** Uumhu... (sound means yes).

**Does she lend you money or just offers help when you have no money for refill?**

She just gives you. Eeeh she just gives you.

**You have never gone to her then she did not give you money**. aa... (No)

**What do you do for her?** There is nothing. It is just her kindness. She helps me in everything, clothes for children, she is a tailor, you just see her sending a bunch of clothes for the children. She also gives me clothes to wear. Recently my 3 children needed uniforms. In this school uniforms have been increased from 11,000/= to 15,000/=. I told her my children had no uniform but I did not have money. She told me to give her only 20,000, then she would buy for me material and sew the uniforms for free. She is already sewing the uniforms for the children. If I had bought from the school, I would have paid close to 50,000/=. Sewing a uniform for a person like the big boy may cost up to 5000/= in this place. She forewent all that income. She is a real friend. She gives me everything, even food. We do not have a banana plantation, on important days she surprises you by sending you a bunch of *matooke*.

**Do you always go to thank her?** Of course I do. (laughs). I always go and greet her. Even when I am tired of keeping home and i have finished my work, I visit her and we chat. I do not even give her or do anything for her in return, except, when she has shoes that need fixing, since my husband is a cobbler, she collects the shoes for the children and herself and brings them here for fixing. He does not charge a single coin. He says this woman does a lot for us, I can’t charge her even 100/=.

**Are you the one who lobbies for her not to be charged?** Yes, but he also sees it. She just gets clothes for children and gives me to take to them, her children have many clothes. She buys all the time, but also sews for them. She sews clothes for my children too, using material that sometimes you look at and think it is very expensive.

**We were talking about the things you consider important for the management of HIV and related infections such as the blisters. Which things do you consider important to have in the home?**

Laughs... silence

**Which things do you consider important resources since you learnt that you are HIV positive that you never attributed so much importance before you knew.**

Silence, laughter... silence, important things...Before I started medicine, what I saw... but I don’t have much problems. What is disturbing my peace most is this skin. The skin is the only issue I see.

**What I am saying, which things do you consider important to have, for instance, some of your fellow PLHIV told me that having medicine in the house is key, because you can fall sick anytime.**

Of course medicines (ARVS) are the first. I always ensure that I have ARVs. I have never skipped an appointment. It is also important to have medicine that relieves fever, when it attacks you. This medicine (ARVs) does not relieve fever, it is important to buy medicine like panadol, coartem. For instance, yesterday I felt feverish then I bought panadol and coartem and swallowed. **Esther: May be the blisters are coming?** It is possible. **Esther: Is that how they present?** Yes.

**How much coartem do you normally buy?** For 500 shillings. **How many tablets are these?** About 6. **Coartem?? I thought coartem is sold according to the recommended dose?** They give you what you want. If you want a dose, they give you. **Did they conduct any blood tests?** No. **How many tablets did you swallow?** They told me to swallow 4 tablets of coartem and 2 panadol. I swallowed, now I am feeling better. **Esther: Do you sometimes treat the blisters without going to health facility?** Yes. My blisters are small but they are very itchy.

**How do you manage them?** They are like that. I do nothing. For me sometimes I mix salty water with the vaseline I smear when I don’t bathe salty water. This relieves the itching a bit when the salt penetrates in to the wounds.

**Where did you learn that from?** Umhu, it is pain for my children. When the child’s body starts to itch he does the same. Before we learnt the option of bathing salty water he used to put salty water in Vaseline before smearing himself. (She touched the boy’s hand. Who was sitting beside her) I see, they are about to come (the blisters). **So how did you learn about the option of bathing salty water or mixing it with Vaseline?** Uumhu, nowhere, it is all a matter of struggling. One time they told me about a salt mineral block. Do this and smear yourselves. Do you know it? **Esther: Is it the thing the cows lick?** Uumhu. They told me to buy it. We used to have it at home, but these days I am lazy to buy it. I would touch it and smear, I would sometimes put it in water then we would smear the water all over the body. It works. The itching would stop. But sometimes this water would not work for the boy. So I would put the mineral salt in vaseline, then he would smear himself. When the salts enter the wounds, the itching reduces and sometimes ceases completely. You could then get some sleep. The things can itch you until you fail to sleep. If you wake up and mix salt with water, put it in Vaseline and smear, you get real sleep. **Esther: That is interesting. I had never heard that the salt mineral can be used for such purposes.**  (Raises her voice and says emphatically) The salt mineral works for real!

**Esther: Who told you about the salt mineral? P**eople we meet in health facilities. **Esther: Bantu buntu (lay people)?** Yes

**Esther:** **Is there any person you have met at the HIV/Clinic who has seen your skin and advised you on how to manage the rash?**

It is only the counsellors I have heard teaching. There is one man, or what is his name... (she scratches her face and frowns as she tries to remember but fails and proceeds) He said if you don’t want mine, you can buy ingredients and cook for yourself. He told us a list of things to buy, *kanzironziro* (not sure of botanical name), lemon, jik (bleaching detergent), protex (an antibacterial soap), and... i don’t know what. The things were about 6. We were supposed to cook all these things together and also buy 10, or 5 tins of tip-top vaseline, liquid dettol and mix them, then boil, stir them and put them aside to cool. You then smear yourself. I hear this concoction works.

**Esther:** You were telling me about the counsellor, do you know *kanzironziro*?

**Kagoya:** I don’t know it. But he said he had it. Even when you go to shops that sell indigenous things, you will find it. It is also available in the market. She explained, you get jik, scrap pieces from the protex, get the tip top vaseline, it serves as water, boil them until they are ready, get them off the fire and squeeze the lemon while they are off the fire, mix them well, allow them to cool, return them to the tins. When you finish bathing well with your salty water, dry yourself and smear the whole body. That it also works. When he is the one who has cooked it, a tin goes for about 15,000/=. When you cook for yourself, you may save some money, and perhaps spend in 1000s.

**Esther:** Have you tried it out? No (laughs). **Esther:** What are you waiting for? (talks while laughing) I haven’t yet got money. **Esther: How long have you known this information?** Not long. I learnt it from the most recent visit to the HIV/AIDS clinic.

**Esther: We have explored the medicines. So you normally use panadol and coartem, do you keep them here or you just buy?** Most of the times we buy. When we buy we keep it in the house for first aid. When we feel feverish, we swallow some.

**Esther: How about cough?** It is not a major problem except when we catch flu. Still it is not the serious cough.

**Esther:** **Besides medicine, which other things do you consider important for the management of HIV?** Silence... **Esther: How about food? Do you consider it important?** Yes, food is important. The medicine needs when you have eaten something then you can swallow it. Swallowing it when you have not eaten (laughs) it will treat you badly. You may get dizzy sometimes.

**Esther: We have talked of medicine and food, which other things do you consider important to use at home?** Silence... (she seemed to be finding problems with answering the question. I decided to provide some cues)

**Esther: sleeping well... (then I remembered) Many of your colleagues mention money, do you also consider it important?** Money is also necessary. It is actually the most important. **Esther: More than medicine?** **(**she had second thoughts) laughs and says, but medicine is more important, my friend even when you have money, how many rich people die and leave the medicine there after paying millions. I would rank medicine first.

(I asked why but she could not give a direct response, but emphasised, that medicine is the most important. Then I asked what she considered second to medicine, could it be money, she said yes and added having a good life (*okuberako obulungi*), *okuberako obulungi mu nju omwo* (literally being okay in the house). She added, that all are necessary).

**Esther: Medicine, money, food, how about care takers (abajanjabi)?** I don’t have any. I do not consider them important. I am able to do all my work. Apart from the skin that is disturbing me, none of my work can defeat me to do. I even dig. If I do not have malaria or any other illness, I dig in the morning then I come home and execute my duties.

**Esther: How about friends?** I do not have any friends in this neighbourhood. **Esther: But do you consider them important in the management of HIV?** friends... (silence). In this village I do not have any. **Esther:** But you told me about the pastor’s wife? That one stays in the trading centre. **Esther: That means eddinni (religion) has helped you a lot.** Yes it has helped me a lot, it has helped me for real. Praying lifts my spirits. When you tell the pastor about your problems, they read for you verses in the Bible, then your spirits are lifted. I would not be doing any more work, because of the way people backbite me. Since we were in the former place, I and my son have been the talk, talk, talk, talk... but when I pray, I feel my spirits lifted deep inside. Like here these women talk about me, they back bite me. They are always on me. I just walk past them and sit here and do my work.

**Esther:** How about at the health facility, which things are important for the management of HIV? silence... medicine, health workers, what we want is for them to work harder, they made for us medicine and renewed our hope, you stay there and feel strong, but these things (touches her hands to show me the rash on the skin). The medicine has worked for me. Also diagnostics are important.

(I asked her to rank the resources, but she was not decisive on which were more important. She instead told me that it is necessary to talk to the health workers so that they can lift their spirits. Whenever she goes, she talks to one of the counsellors about her skin and that of her son. He always urges her to go to the skin clinic of Mulago. I urged her to try going back one day, but promised to bring her the concoction I had seen at one of the facilities. I told her several people had told me it worked for them).

**Esther: which things do you see as important for you and your son who are on medicine, but may not be particularly important for the rest?**

(she keeps quiet for about 2 minutes and then says), like what? **Esther: May be you see no difference.**  Like these children? **Esther:** **Yes, those who are not infected with HIV.** Those who are okay don’t need much. We the sick ones need health care (*obujanjabi*).

**Esther: Have you involved in any associations for PLHIV?** No. **Esther: have you heard of any?** Yes, but I haven’t seen any in this area, but I used to hear about them in the former place, but I didn’t participate in any.

(I asked if she has ever skipped a clinic day. She told me on one occasion. She forgot the date and realised later. She decided to go. They cautioned her but gave her the medicine. I further asked about the availability of safe water. She told me they use water from a protected spring. They use this same water for drinking and do not have a specific storage container for the drinking water. I asked if they had been given a jerrycan, she said they had not, and whenever she tells the health workers they do not give her. I urged her to insist, because I had seen some jerrycans being given out recently. I asked if they sleep under mosquito nets, she told me they were given, not by the hospital but they received ‘Museveni’s ‘ mosquito nets which were distributed at village level. We were interrupted by her young baby who was crying for bread. She had told me earlier that she had sent for that bread as her lunch. When we started talking she somehow deferred the eating. The baby was now fighting with her big sister for the bun. This reminded me that we were yet to talk about how she schedules her meals. So i took the opportunity to ask whether she prepares a morning meal for her and her son to be able to take medicine. She told me she doesn’t. If there are leftovers for last night they eat and swallow. Much of the time she and the boy take tea and swallow the medicine because there is nothing else to eat. I asked if this does not affect her, she told me it does, but she has nothing to do, she has to swallow the medicine. She started looking for some money and asked the children if any of them had her money. I noticed a 200/= coin behind her and told her the money was behind her. She gladly picked it and put it in her pocket and then told me she always feels bad when there is no food yet her son has to swallow medicine and go to school. **Kagoya:** What I do, I give him some money. Even when I have only 1000/= I give him at least 200/= per day, so that he can buy something to eat. It is not good for him to stay hungry until 10:00 O’clock when they are given porridge at school. When he gets nothing to eat, you look at him and feel bad. When I had my cassava, i would buy cooking oil of 500/= and fry him some cassava. He would eat it and drink enough water, swallow medicine and then go to school I would be happy. But now I don’t have any cassava that is ready to eat. It is difficult. When he goes hungry my heart pains. I always forego meals to ensure that he gets something to eat. Sometimes I warm the food that remained overnight then I give them to eat before they go to school. When there is no food, I boil warm water and give them even if there is no snack to eat with it; it at least warms up the stomach. The rest of the children do not bother me as much as my sick son.

**Esther: What support do you receive from your relatives?**

I do not have any relative who supports me. **Esther: Even your mother?** My mother aaaaah... **Esther: Did you disclose to them?** Yes all of them know, even those in my home village. But we talk. They used to call me when we had a mobile phone. These days when I feel like talking to them I go the neighbour’s place and call, but rarely. But they do not give me concrete support when I am sick. Nobody has ever sent me money or anything for instance. It is instead the pastor’s wife who helps me. But the good thing, I rarely fall sick. In the former place, I got so sick to the extent of becoming bedridden. Since I came here, God has helped me, I have never experienced illness and got bedridden for more than a week.

**Esther: Did anyone help you when you were in the other place?** No. There is a time I fell sick and decided to go to my older sister’s home. I had just lost my first husband by then. When I reached my sister’s place, the sister told me she was not comfortable with my son’s presence. That the boy looked awful, her husband would quarrel because I had brought a sick child amongst his children. I just packed my bags and went back to my home. That taught me a lesson, if you see me falling sick and moving away from my home to another person’s home, then you will know that the illness is very serious. That time I had not yet started ARVs, but used to be in and out of hospital treating fever. I even tried herbs. People told me about aloevera and *mululuza* (local herb) I would buy or ask from neighbours and boil and drink a little. I hear it is very effective against fever. Even around here somebody has it. I can ask when I need it. I still use herbs to treat cough and fever that afflict both me and the children. **Esther: But I hear the counsellors telling you that ARVs and herbs cannot be mixed, what do they mean?** I am not certain but it must be herbs that people use to manage HIV. There are people who us the jerry cans, but what I know is that they do not work. All my cousins who used to use these herbs for managing HIV all died long ago. When you reach our home village, it is full of graves and no people.

(The landlord interrupted us when he came back from the garden. She told me he does not charge them money. They had spent two years without paying. He had asked them to come here to help him guard the place. He feared that thieves would steal the metallic door shutters. **Kagoya:** We also help with other chores, for example weeding even when he has not asked us to. He is a good person; he gives us some tomatoes, green pepper etc).

**Esther: so your relatives do not support you.** No, I can’t even ask them for anything. When my sister chased me, I learnt a lesson. The medicine has helped me. The most I stay bedridden because of illness is 2 days. And when I can’t do domestic chores my husband helps. He fetches water and cooks food for the children when I can’t. (I commented that he is a nice person, even though he sometimes gets drunk and insults her. She nodded in agreement).

I then asked her if she had any questions for me. She asked me the cause of not menstruating once again. I told her I would find out and come and tell her, but first I needed to see the combination she is on. She sent her son for the books. Meanwhile I asked her which contraceptive she was using. She had earlier told me she did not want more children. She said she was not using any contraceptive. She did not seem worried that she could get pregnant. I also asked her if she swallows the Septrin. She said she swallows it every day, that she was told she has to use it for life, it prevents cough and other illnesses (she however explained the importance of Septrin with some doubt. She did not seem to be very certain about how it actually works). I asked about how the ARVs operate. She spoke more directly but also used a word ‘*simanyi*’ (an equivalent to I don’t know) which suggested some level of doubt. When I asked which of the two, ARVs and Septrin she considered more important for her condition, she said it is ARVs because they weaken the virus. I asked if she suffered from any specific side effects when she started ARVs, she told me both of them got no issues. I asked if her husband has attended any training on HIV/AIDS and the use of ARVs, she told me he has not. Then she said she had a question and I gave her opportunity to speak. She said that they say the ARVs for children and adults differ, but she and her son are always given the same tins. I noted that perhaps the dosage is not the same. She said they swallow the same number of tablets. I asked where she got the information that ARVs for children and adults differ. She told me she had heard over the radio that ‘Rv’ for children is different from that of adults. I was amused I did not know that such information is aired on local radios. I asked her if such programmes are frequent on radio, she told me she always hears them. When I asked what she had learnt she skipped the question and took me back to the issue of being given the same medicine as her son. I noted that what i see is that it is very young children who are normally given different Rvs from adults, but her son was rather old. She seemed to agree and told me that her girls were given different Septrin from hers when they were babies. The boy also used to take different Rvs when he was younger. She seemed contented with my explanation after relating it to her experience. I took her back to the issue of radio programmes. She told me she often listened to programmes that discuss HIV/AIDS issues exclusively and knew when they are aired, but is more interested in listening to programmes aired by Pentecostals, because ‘they lift her spirits (*binzizamu nyo amanyi*). For PLHIV religion is an important thing. When you listen to sermons, you become strong hearted, minimise thoughts and go about your work with determination and hope*.*’

I asked if she can distinguish between illnesses caused by HIV and those that are not. She kept quiet and smiled sheepishly. I said, you don’t and just treat whatever presents using all your knowledge biomedical and herbal. She laughed and said softly, yes. Whatever they advise me to use when a child is sick, I try it. The boy came back with the books. She turned towards him and showed me his legs. You see, they are full of scars. The blisters keep coming back from the same spots. When he heals, shortly after others develop. If they were not continuous perhaps the scars would have disappeared. I asked if they have ever tried tablets, besides the tubes. She told me they had been given medicines and tubes in one place, all sorts of tubes. She used to smear the boy and sometimes he would look like ash. That time the rash was bad and people would talk. Whist in the former place, she had tried to seek employment in a restaurant to look for 1000/= to take care of her son. She got a job in one restaurant. She worked for 2 weeks only. When her boss looked at her critically, she told her to seek treatment and then return. She went and talked to a restaurant owner next door. This one empathised with her and gave her opportunity to work. But the workers in the restaurant she had been working in always persuaded her new boss to chase her, that she would chase away customers. She was nevertheless allowed to work and worked until she got tired and left on her own will. ‘I used to help with the preparation of porridge for the children in this school when the cook left. The head teacher used to give me some money once in a while. But when she scrutinised my skin, she started backbiting me and even stopped me from cooking the children’s porridge. I also lost interest when I heard that they were back biting me. If they can talk about you like that, can you touch their food?’

I asked her what was preventing her from asking for a job. She laughed and said, if she prepared snacks or food, nobody in her village would eat it. ‘With the way I look, if I brought tomatoes or silver fish, nobody would buy them, people in this village can talk.’ I suggested that she could try looking for jobs that are more physical like digging. She told me those jobs are there, but many times they are available when she has a lot of domestic work to do. She used to dig when she had just come to this new place. She had just weaned her two year old baby and did not have enough money for milk. She used to dig for money and would manage to raise enough to pay suppliers of milk on a monthly basis.

I was given the books. They were neat, and not creased, unlike many I see at the HIV clinic. I thought I knew why, she doesn’t hide the books like many patients have confessed to. I noted that both mother and son were on CBV/N (lamivudine-zidovudine/ Nivirapine). I wrote the combination in my book and promised to inquire if the amenorrhea she was experiencing was due to the combination. I said goodbye and promised to come back with the concoction for the skin the following week. It was late, approaching 4p.m by then. I realised the pile of clothes was still lying on the ground untouched. When I asked if she would manage to wash, she said they would wait for tomorrow. She took me inside the house to see the herbal ointment her husband had bought lately. I looked at the ingredients and noticed that one of them was ‘*kanzironziro*’ the mystery plant that the counsellor had talked about when he was teaching them how to make herbal concoctions for the skin. She said it was yet to work. They slept in one room. The parents on the bed while the children on the floor. The bed was separated from where the children slept by a curtain. The room was spacious I thought perhaps they fitted, although what came to my mind was how they ensured their privacy when the children who are of age were just on the other side of the curtain. Her ARVs and those of the boy were placed on some stool besides her bed. I did not spend much time in the house. We then started walking towards the road where I had parked my car. I complained that the road was impassable then she showed me another route. As we walked she shared with me that the pastor’s wife had offered her an opportunity to hawk some of the clothes she sewed then they would share the proceeds. I told her I thought it was not a bad idea, but I was concerned that it was strenuous work. She would have to move from door to door under the heat, and then where would she leave the little girl who does not go to school. She concurred with me, and told me she had hesitated to take it up because of the same reasons. She noted that the pastor’s wife always thinks for her what to do, she wants her to work. She continued, May be I should go back into serving food in restaurants. But I am worried that they will chase me as soon as they see my skin. I suggested that she should buy long sleeved blouses to cover her skin. She thought it was a plausible option. She also told me that her husband had proposed to relocate them where he had been given family land. He was yet to implement the decision because he was waiting for her position on the issue. She asked for my advice. I asked what she had in mind. She told me she thought it was better to sell the other land and buy land here. *These people especially those in my church love and care for me. Also my relatives are not so far from here,* she said. I told her that was a good idea. She should sell it to her husband and see what happens. We reached the roadside and exchanged good bye.
