# Supplementary material for: Social capital and resilience among people living on antiretroviral therapy in resource-poor Uganda
Source: PLoS One. 2018 Jun 11;13(6):e0197979. doi: 10.1371/journal.pone.0197979 (PMC5995438; doi:10.1371/journal.pone.0197979)
Supplement: S3 File — (DOCX) [file pone.0197979.s004.docx]

**Client ID: 003**

**Name: Matana (Pseudonym)**

Status: **ART (TDF/3TC/EFV)**

**Background**

I met Matana on a clinic day when I was talking to a peer educator about the insurance scheme he had just introduced to the patients. He had informed them that with a contribution of 1500/= per month, they could pool resources to meet their treatment costs when they fell ill. A few patients had paid up and he gave them receipts. My concern was that this scheme was not registered anywhere and that the patients could be cheated. I had sought him out to educate me about the scheme. We had just started talking when the nurse dispensing medicines that day brought Matana to the peer educator and asked him to re-educate her about how to use the medicines. The nurse explained that she had discovered that Matana was taking both hers and the child’s medicines (ARVs). She reprimanded her for not paying attention as instructions are given and accused her of always being in a rush at the dispensing window. Matana became defensive and started arguing with the nurse. The peer educator was forced to intervene to calm the situation down. He calmly inquired about how she has been using the many tins of medicine. They were eight in number (2 for her and 3 for each of her two children on ART). Her explanation showed that she was overdosing the children. She gave them the same medicine from separate tins twice instead of once a day. The children were taking 6 instead of 3 tablets. She too was overdosing on both her drugs and those of the children. The nurse said that Matana had not listened to her advice to buy polythenes to pack the medicines separately. When the nurse left, the peer educator gave Matana money to buy 3 polythene bags. He checked their books, identified and labelled the drugs for each of them and then took her through the prescriptions. He advised her to keep either book in the polythene together with the medicines. I asked to talk with her afterwards.

**Section 1: Socio demographic characteristics**

Age:  **35 years**

Sex: **Female**

Marital status: **Widowed**

Highest education level attained: P.2

Main Source of livelihood: **Subsistence farming**

1.8 Ethnicity: **Muganda**

1.9 Household size: **4**

**Esther: What was your experience with the medicine:** oh! It made us sick. I personally felt dizzy whilst the children were weak. I could not work or even attend to my domestic duties. My grandmother decided to send me one of his relatives to help me at home. I was forced to sell the pigs I was rearing to sustain the home. When we came back here after two weeks they told us to take a lot of fluids and that we would be fine. After about a month we got better. We have been fine, but a couple of weeks ago we got sick again. The children were sick, i was sick, i didn’t know what was going on. I went to a HCIII then they said we had malaria and told us to buy some medicine, which I did. The sickness did not go away. I kept wondering what was wrong. But today when we came the nurse said we were taking the medicine wrongly. **Esther: what happened? Did they make any changes that confused you?** They were giving us 2 months because they wanted to monitor the children’s progress closely. This little one has been sickly, she had improved only recently. The last time we came I asked for 3 months. I talked to the *musawo* and told him that I was overburdened with the transport costs. I don’t come from near. I need 20,000/= every time I come, because I have to transport the children and also feed them whilst we are here. I told him it was costly. He understood and decided to give us three months. The tins became many and confused me. When the musawo asked me how we have been taking the medicine i could not explain properly. I was confused. She counted the tablets in the open tins and said I was giving the children too much medicine and mixing theirs with mine. Now I know why we have been falling sick. **Esther: I hope the polythenes will help you to manage the medicine better.** I hope so. (she asked to excuse herself to buy the children something to eat. She got them sodas and cakes. She left them eating a few metres away and came back for the interview).

**Now, let us talk about the illnesses that disturb you and the children most**

Fever and cough for the children. Like I have told you we have been very sick. Those children have disturbed me for years, although now there is an improvement. **Esther: Are they in school?** No. They have been sickly for years. I could not take them to school. **Esther: But they look quite old to me. How old are they?** The boy is 8 and the girl is 6 years. **Esther: eeh you need to consider taking them to school.** Matana: I will see.

**Now let us look at the resources considered necessary for the management of HIV/AIDS at home**

**Food**

A person with HIV/AIDS is supposed to eat well. Eat matooke, meat, rice. But many of us cannot afford to eat well. We mainly eat sweet potatoes,’ but you get tired of eating sweet potatoes all the time. I grow much of the food we eat, although the land is small. I don’t know what I am going to do when my in-laws give away part of the land I am using. They are saying that I am using much more than what my husband was given. My brother-in-law who lives close to us says it is his piece and that if I want to use it I should first get married too him. **Esther**: **Doesn’t he fear that your husband could have died of HIV?** They say that HIV was not made for animals. They don’t fear it.

**What else?**

**Medicine**

‘Medicine is my life. I always ensure that I attend the routine clinic to get the medicine. I have attended all my appointments so far. I swallow Septrin and RV (ARVs), but other illnesses require their own drugs. When I get fever, I go to the HCIII for treatment. But sometimes the health workers there chase us. When you give them your book then they realise that you are on HIV medicine they tell you to go where you get your medicine from. It happened to me recently. The musawo looked at my book and said, go to the treatment centre, we can’t manage your condition. **Esther: Do you find the medicine in the HCIII?** Sometimes. When it is not there they write for you then you buy. There are many drug shops in our trading centre.

**Money** – with money you can get whatever you want.

**Sleeping well, a good home-** You need a bed, beddings. Getting sleep from the floor is difficult. I sleep badly. My husband left me in a mud and wattle hut. We can hardly fit there. It is now almost collapsing on me. An anthill developed within the house. It brings mushrooms. I used medicine to remove the anthill, but the house has gaping holes, snakes enter though them. My children sleep on the floor. One of them found a snake in their clothes. I need a lot of money to get a better house.

When I asked her to rank the identified resources she ranked them as follows;

Money is the first. She noted, ‘money makes life easy (*sente wekuba egonzawo*). When you have money you will accomplish everything. She followed it with medicine, good food and then others.

**Resources at the Health Centre**

**Food**- We come here before eating anything. I have to leave my home as early as 5:00am to catch the only taxi in our village. Actually they pick me from home. I tell the driver overnight that I will be travelling. Moving away from home at that time is risky. When the taxi leaves you, you either forget the journey or use bodaboda which are very expensive. They bring me at 3000/= in the taxi. I would need about 10,000/- on a bodaboda. Then what would I use to , feed the children and then return home. If they could find a way of organising food for us here.

**Medicine**-This is the most important at health facility level followed by **health workers**. The health workers here are good, they care about us. **Esther: How about diagnostics?** Yes, it is important to know how you stand.

**Other resources**

**Support-** in form of money, food, paraffin, sugar and other household needs. We need an organisation that can help. Some of my fellow widows got help from an organisation that built for them houses. I would have loved to benefit but I did not get information in time.

**Income**- You need a source of income. Transport requires money, treating illnesses, buying food. We dig but the drought has not treated us well. (We are interrupted by one of the children who whispers something to his mother. He wanted something else to eat. Matana excuses herself to go and get her something from the canteen. The line at the canteen is long. She takes about 10 minutes to return). **Esther: you were explaining why having a source of income is necessary.** Yes, now like me, I am the father and mother. I have to fend for the home, then I need money to come here. I have to pay user fees for the 3 of us, add transport, sickness. One needs money if they are to manage HIV. **Esther: What is your major source of income?** I do casual labour. The rich people know me. When they have a job they come here and alert me. Like yesterday one of them came and told me that he had a job for me and that I should go and see if I can take it up. **Esther: what kind of job is it?** He wants me to open up some land for cultivation. It is bushy right now. I will go and see if I can manage. Since I have been sick, I may do it with my relative at home, he is an energetic man. Who knows, it may provide the money i need to fund the next visit.

**Esther: What problems do you encounter in accessing the resources you have mentioned above?**

**Poverty:** The biggest problem is poverty. There is too much poverty here in the villages. At times you can’t even find 1000 shillings to buy cooking oil. We eat poorly. We have to buy much of the food because the weather is not good. Like me, I have failed to construct a better house. We just squeeze ourselves in the small hut my husband left me.

**Transport:** moving here to the treatment centre is expensive. Like I said I need at least 20,000/- to accomplish everything. That is a lot of money for a person who resides in the village. I can’t save any money because I keep every coin for the visit to the health facility. *Obulamu mbwagala* (I want to remain alive). **Esther: Have you ever missed any appointment due to lack of transport?** No. I have managed to come on all my days. When i realise that the month for the visit is approaching I inquire from the rich people in my village if they have any job I can do. They normally have, so I take it up and raise the money. Sometimes I have to take more than one job to be able to get enough money. It depends on what work is available. The few times I have failed to raise the money in time, I have asked my paternal aunt for a loan. She is married to a reverend and lives a walkable distance from my home. I visit her and explain my challenges. I have learnt her over the years. When you ask her for direct financial help she gives all sorts of excuses, but responds fast when you say you will pay back at an agreed time. She usually bails me out. **Esther: How about other relatives. Do they support you?** No. My father has many children and wives, so he is not very close to me. I told you I was the only child of my mother and grew up with my grandmother. Yes, my grandmother cares but she can’t do much for me. Instead it is me who supports her. **Esther: How about friends?** Ha...i don’t have people i can count on as real friends. These people in the community would just laugh and gossip about you if you shared your problems with them. We talk but I never share my personal issues with anyone, especially regarding these issues of the health facility.
