# Supplementary material for: Social capital and resilience among people living on antiretroviral therapy in resource-poor Uganda
Source: PLoS One. 2018 Jun 11;13(6):e0197979. doi: 10.1371/journal.pone.0197979 (PMC5995438; doi:10.1371/journal.pone.0197979)
Supplement: S4 File — (DOC) [file pone.0197979.s005.doc]

**Client ID: 005**

**Name: Micheal (pseudonym)**

Status: **ART (CBV/N)**

**Section 1: Socio demographic characteristics**

Age: **45 years**

Sex:  **Male**

Marital status: **Married**

Highest education level attained:  **Primary Four**

Main Source of livelihood: **Cultivation**

Ethnicity: **Musamya**

Household size: **8**

**I met Micheal at the treatment centre when he had come to do a CD4 test. He had come straight to the triage and asked for CD4 request form. He told me it was overdue. He had been told to come here for a CD4 test the last time he got refill from the outreach, but he had been waiting for any opportunity to bring him to the health facility or anywhere close so that he could hit many birds with one stone. ‘Transport to come here is difficult, I thought of spending my money to come only for a CD4 test and go back, and I thought it was not economical,’ he told me. He had business to do in a town nearby and so saw this as a golden opportunity to test. I thought I should get an interview from someone who benefitted from outreaches and asked to talk to him after the test. When he returned I excused myself from the triage, introduced myself to Micheal and asked him for an interview which he accepted to give me.**

**HIV/AIDS experience**

Before we swallow medicine **w**e first go and they test us to see where we stand in our life after learning that you have HIV, the health workers explain to you that you have HIV and give you Septrin for 2 weeks and keep counselling you. When the CD4 is low you may begin medicine. But if it is high, they may leave you on Septrin. **Esther: what was your CD4 when you started medicine?** I do not remember the exact number but it was in 70s. I felt feverish that time but I did not know, until my wife complained about the persistent fever and suggested that we go and test. At first I refused. But eventually I decided to come to the treatment centre to test. That was 5 years ago. They told me I was positive and that I should come back with my wife so that we are tested together. When we came they said she was also positive. Both of us were enrolled here but eventually my wife decided to change to another facility. At first I was given Septrin. When I went back they told me to go for a CD4 count. The results came back after two weeks. They started teaching me about medicine and after two weeks I started.

**Resources considered important for management of HIV at home**

**Clean water**: they say it is good for my life. They always teach us to boil it. We get our water from a borehole, boil it and put in a jerry can. We were given jerry cans. They also used to give us some tablets to put in it, but I failed to use them. Whenever I would drink the water I would feel like vomiting, it had a smell. It is the one that discouraged me from taking it.

**Food**

Food is very important for us. Time comes when you feel like eating particular food like matooke, but when it is not available. We always plant matooke but the banana wilt attacks it and it gets spoilt. We mainly eat sweet potatoes. We grow most of the food we eat. For a change in diet, you have to buy. Sauce is also a problem. We mainly eat ground nuts, my wife normally grows some. I also rear animals like pigs, goats and cows. At least we get milk from the cows.

**Money**

We need money for everything. Buying food, paying school fees, the children have to study. Buying food, transport to come here. Life thrives on money.

**Medicine**

Medicine is very important because it is the one that keeps me alive. I always try to take my medicine on time. I have to remain alive to raise my children. They are still young.

**Ranking of resources at home**

**Medicine** – without it I would be a dead man.

**Food-** You cannot swallow that medicine without eating. It is very strong.

**Water** – All the cooking and eating rotates around water. Swallowing medicine requires water.’

**Money**- I have needs, buying clothes, meat, matooke to change the diet a bit, all these require money.

**Common illnesses**

It is rare that we fall sick. May be flu and cough and sometimes malaria. The medicine for malaria is normally there in the government health facilities and that is where we go.

**Resources at health facility**

**Medicine-** We are happy when we find the medicine there. And when we do not find it we feel bad. Many of us put in money to go to hospital to get money, sometimes after selling our property. It is disappointing to do all this, then you find no medicine at the health facility.

**Health workers**- Some of these health workers are rude. We go there because we are sick. Sometimes you are trying to show them where it is paining most but they are shouting at you. By the time you go home your heart has sunk. This is why some of us get fed up, stop swallowing medicine and say why don’t I die, after all I was made to die.

**Transport**- ‘Some of us come from very far. Such a service at the health facility would be very helpful, even if it is not all the time. Sometimes we reach here and find difficulties, yet they expect us not to skip appointments, but we also do not want to miss appointments because we want the life. Sometimes the money refuses, sometimes you get a friend to bail you out, but fail to mobilise transport to return home. If you go and they help you with transport back home, it would be good.’

(We were interrupted by a call from someone he said he had to pick. After the call we continued exploring the resources).

**Diagnostics-** the laboratory should have the personnel but also the things for using, for them to be able to follow our condition well.

**Ranking resources at the health facility**

**Health workers**- They are the ones who use the other things. They are the people I get to first, then they decide what tests I should do and do the tests then give me medicine.

All the things that are used such as diagnostics and other things like gloves that the health workers need to use and to know the status of the patient.’

**Medicines**- the medicine is given after understanding where I stand. Without a health worker, I may not be able to access the medicine even when it is available.

**Other important resources**

**Food** (says it with emphasis) - people on medicine really eat. When you start medicine, you can really eat. **Esther: Have you noticed a difference in your eating since you started the medicine.** I started the medicine when I was light. But I added some kilos and got more energy after starting. By the time I started, I was not eating. My appetite was very poor. I started eating, I started eating a lot the moment I started the medicine, sometimes the things to eat were not really there, I had to sketch for them, our place is dry. But they [medicines] worked for me, even though they were few, they replenished my energy. We eat sweet potatoes, leafy greens, they teach us to look for leafy greens. During the rainy season we plant some greens. When they get finished we devise other means. Sometimes we buy tomatoes. During the dry season we eat any food that is available. When you don’t cultivate ground nuts for sauce, you eat sweet potatoes without sauce. Sometimes we boil warm water and take it with the sweet potatoes and sleep. We eat only twice a day, lunch and supper. In the morning there is no time to eat, we have to dig. (He had told me he takes his medicine at 9.00am, so I asked him how he manages without food). He said he just swallows the medicine without food. It does not do him any harm, as long as he takes a cup of water.

**Esther: Did you get any side-effects when you started ARVs?** I did not get any serious ones, except headache. I did not understand why I had frequent headaches at first, I thought giving myself a break from the medicine and observe what would happen. I decided to first consult a fellow patient I knew had been on HIV medicine for while, to find out if he had got a similar challenge. When I went to him he asked me if the tablets I was swallowing were blue, then I said yes. He told me that he too had got headache and that headache is common among people on this particular medicine. He told me to drink a lot of water and to buy hedex (a strong pain killer). I followed his advice and got better over time.

I then reverted to the question we were discussing and asked him to identify other resources he considered important for the management of HIV/AIDS. He was silent for a while and seemed stuck. I probed if he considered relatives and friends important resources in HIV treatment.

**Micheal:** Yes, friends are helpful. I always consult my friends who are HIV positive. We visit each other during illness. There are not many people with HIV who are open about their status. But we know each other. We meet at the HIV clinic. Some of us are willing to help others with information. For example many new patients have confided in me that they want to stop ARVs because they mistreat them then I encourage them to persist. I always draw on my experience to convince my colleagues that side effects are manageable and that they should not throw the medicine away. When I am stuck, I always consult some of my friends like I told you about the headache.

**Things he considers critical since he learnt of his status, but did not consider so before**

(Laughs) and says, ‘When you do not fall sick, you would never know that medicine is important for life, aaa, you first fall sick. Since we tested we having been taking this medicine, we do not use anything else. The counsellors told us not to mix this medicine with herbal concoctions. **Esther: Do you use herbal medicines for treating other conditions such as cough, flu, etc.** Those ones we use, for instance *mululuza*, *bombo*, especially to treat cough among children. **Esther: Why do you give herbs to particularly children:** (laughs)...my wife and I detaste bitter things. I force the children to take them when I have no money to buy for them drugs. But when I have some money I buy for them syrups. **Esther: Where did you learn to use herbal medicines?** Yiii... my late mother taught us, we would always see her giving them to me and my siblings. **How about malaria? Do you sometimes treat it from home?** No, we always go the public health facility, there is medicine for malaria. We only try out clinics when the treatment obtained from the public health facility fails.

**(We were interrupted by another phone call. Micheal stepped away to receive it and took a few minutes talking)**

**When he returned I asked him about the support he gets from his relatives.**

He said, ‘It depends. Some of them help whilst others don’t.

**Esther: What problems do you face in accessing the resources we have been mentioning above?** From my view the biggest problem is **poverty**. A person with HIV without money dies early. You eat badly, you sleep badly... it is horrible. Other people [those without HIV] may easily eat food that fills the stomach like posho because it brings energy, but a person on medicine may not like it. Like me, I do not like rice and posho but I eat matooke (plantain), yet a bunch costs over 20,000/=. I am lucky sometimes I harvest a bunch or two from my garden, but when it is not ready I have to buy once in a while. We are also encouraged to eat fish, but it is expensive. People fail to buy fish because they have no money. Therefore a person with HIV needs money.

Another thing is **transport**. Coming here is very expensive for many of us. We would for instance have loved to come here more often, whenever we have illnesses but it is far. Coming here is a last resort. Before they brought the outreach it was hard. I would spend about 15,000/- on only transport. Esther: what would you do to manage? I would save. I told you I am a farmer. Whenever i sold produce, I made sure I saved enough money to cater for that. I still do, but at least now I just drive my bicycle to the outreach point. I don’t have to spend on transport. When I had no money I would borrow from friends and sometimes, I would inconvenience bodaboda riders to bring me then I pay them later. Being open about my status many of them never questioned why I was going to the treatment centre. They would instead empathise. I would tell them, my friend, my medicine is finished I need to go to the treatment centre to collect more but I have no money. I hope to get money on such a date. I request that you take me.
