# Supplementary material for: Social capital and resilience among people living on antiretroviral therapy in resource-poor Uganda
Source: PLoS One. 2018 Jun 11;13(6):e0197979. doi: 10.1371/journal.pone.0197979 (PMC5995438; doi:10.1371/journal.pone.0197979)
Supplement: S5 File — (DOCX) [file pone.0197979.s006.docx]

**Client 006**

**Name: Betina (pseudonym)**

Status: Pre ART (Dapsone)

**Section 1: Socio demographic characteristics**

Age: **40 years**

Sex: Fem**ale**

Marital status: **Married**

Highest education level attained:  **She did not go to school at all**

Main Source of livelihood: **Cultivation**

Ethnicity: **Itesot**

Household size: **9 (Both Bwire and Betina are living positively and are open about their status. Bwire is on ARVs but Betina is on only Dapsone because she is allergic to Septrin). We conducted the interview whilst Bwire was waiting for her from a few metres away.**

**HIV/AIDS experience**

I tested and got to know that I was HIV positive the same year my husband told you. I had my big sister who stays in the islands (she lowers the voice) she told me we should go and test. For me I was getting boils by that time. We went and got tested. Yusufu (pseudonym) was...what are they called? I said supporter? Then she said, when my results from the blood test came he is the one who received mine.’ I said enthusiastically, counsellor, she said, uumhu, he is actually my friend. My sister also got her own counsellor. (she whispers) I was found sick, but my sister was okay (*mulamu-*literally alive). (she raises the voice) Ho!!, tears. They asked me if my husband knew about it, then i said he did not know. They asked me if I told him to come would he, I said, I would tell him to come. They said what if he refuses? I said I do not know. They said if I tell him and he refuses I should go back to my home and when I start medicine I should go to my home and swallow the medicine. They told me If I had accepted I should come back the following Thursday with a book. We went back home and told our mother. She also cried. Where we stay is far, i would not have got transport to bring me back to the facility the following Thursday. We stayed at my mother’s place. On Thursday, I put my daughter in law on a taxi back home, then I went to the treatment centre. Another son of mine was at my mother’s place. I told him to ride me to the treatment centre on a bicycle. I expected to get, they had told me that I would be given a jerry can and mosquito nets. We went, he waited for me. When they gave me a jerry can and net he put them on the bicycle and rode them home. For me I boarded a taxi. When I arrived, I told him (points at Bwire) everything. He said he did not know anything. I told him they had told me to ask him to go to test his blood and know his status. I offered to escort him. One Thursday came and we went. (She is whispering...) He was tested and found positive. He began medicine. He swallowed Septrin for one month and began medicine. **Esther: you mean he was that badly off?** (speaks with seriousness and emphasis) He used to fall sick, ho! When I was pregnant with my last born he almost left me (died) when the pregnancy was only 4 months. Since he came here to the treatment centre, he has never been as ill again, to date, I tell you at this time he is normally in his gardens. He has never fallen sick again, those days when he would be sick, he would not swallow any tablets. He would vomit the moment he swallowed any medicine. But since he started medicine, he has never vomited tablets again.

They gave me Septrin but I was not taking it. They gave it to me, I swallowed one tablet then it burnt me. Here, here, here (she touched her hands, lips). By the time I tested, I was not taking the usual Septrin. Health workers had barred me from using it, this Septrin for fever; they had refused me to take it. It was once given to me from a public health facility. The moment I swallowed it like this, my body started shaking. I went back to the clinic, then they told me I should never swallow Septrin again. **Esther: so why didn’t you inform the staff of treatment centre**? I told them but they told me to go and try it. When I went home, I swallowed only one tablet, I felt a burning sensation in the stomach. When I went back on the next appointment, I told them then they told me to swallow Dapsone. By the time I went I was black. One would easily ask me if I had smeared soot on my lips. They changed me to Dapsone. But it is never in the health facility. We have had to buy it all these years. We buy each tablet at 300/=. Moving from our place, you need 15000/= to take you to and from the treatment centre, without eating anything. You pay money at the window but when the medicine is not there, you have not got it. Other people get medicine, you they tell you the medicine you use is not available, yet you have deposited your 2000/= at the window. You have to dig deep in your pocket for 30,000/=, because they give you 3 months to return, isn’t that 45000/=? Where would you be working to afford that money, my dear? The way you are seeing us, we live in a village. **Esther: but residents of villages have money.** **Betina:** Ha! There is no money, we do not have money. We just dig. If you say coffee, when will the coffee ever reach maturity, it takes a year. The children want to study, won’t they dress? The sun is blazing hot, we are about to start buying food. **Esther:** I understand most of you have sweet potatoes: **Betina:** they got rotten due to the heat. We are planning to buy maize, a kilogramme is about to rise up to 1000/=. **Esther: so how do you manage?** You see our children go to government schools but we give there some little money. We have 4 children going to school. We used 80,000/=. You have to give them porridge, 3 kilogrammes per child, we take flour. **Esther: I thought you could use some of the maize you planted rather than buying?** Our place is dry, you cannot get much yield from maize. Sometimes the beans also do not yield. It is better to get someone with a bush then you rent it, or you could go to a person with a portion to dig then you provide casual labour, then they give you money. There are villages, which are easy. People there do not pay little for casual labour, but here, someone gives you a portion to dig for only 20,000/=, then you struggle to dig it. Remember, right now we have no energy, like the people who are alive (without HIV) have. Poverty, health worker, don’t mention it... . **Esther: but you look very strong. Betina:** (laughs) Aah, all those children I have produced, haven’t they reduced my energy levels? If I had not gone in to family (contraceptives), I would have had another baby by now. **Esther: what method of contraception are you using?** I first used the injection, but got palpitations and could not dig any more. **Esther: so you and Bwire decided to change to another one?** He did not have to consent. I just told him that I was tired of producing, and if he was still interested in children to get another wife. I have produced a whole 6 children. **Esther: so why did they not disclose to him his status before discharging him?** (whispers) Is there anything he did not know? **Esther:** perhaps. **Betina:** you *musawo*, you joke with people. **Esther: You mean he got you when he knew his status? (**Silence for a while and then whispers, don’t you hear about those who say I don’t die alone. **Esther:** Perhaps they are there, because there are several people who do not disclose. **Betina:** (whispers) I tell you, we get serious problems in regard to money. **Esther:** I imagine you forgave him even if he didn’t tell you the truth. **Betina:** Everything looked like me, my big sister was telling me we go and she gets me something to do, but I asked myself, if I go would I have left the disease here, then I dump my children here to suffer and yet I am going to die then they remain suffering. I thought since I was still alive, I would work for my children, so I decided to sit. Besides the most important reason, he does not mistreat me. He has never beaten me. Even when I have my money and asks me to lend it to him, I say no, but he never takes it. He knows where I keep my money but he can never take it, another person would take it. There are times we harvest much crops, I sell some, but he never asks for any money unless you decide to give him some. I use my money to buy medicine, clothes for children. I decided to sit here because, a person loves you when you are somewhere. I might reach there then we fall out, she will be the one taunting me about my HIV status. **Esther:** I think you took the right decision, you would not have settled there when your children were alone. **Betina:** I was determined, I would have stayed there. I had not produced the last born, the second last born was relatively grown. Bwire found me with 3 children, but he has raised them like his own. If i do not tell you that they are not his, you would not tell. I considered that now my elder children are now big, I would be ashaming them if people started saying their mother had abandoned the marriage. All my elder children are aware of our status. One of them is really caring. If I tell him I have failed to get money, he looks for it and gives me. He always says we are the ones who pushed our mother in to awkward circumstances; she wanted to get a home for us, because their father died. They have their relatives they are rich.

**Resources important at home**

The important things like **medicine** (says medicine with emphasis). Even when a child is sent away from school due to non- payment of school fees when I have no medicine, I don’t pay it.

**Esther: any other things you consider important for the management of HIV/AIDS at home.** (silence) medicine is the most important thing. Previously, you would not convince me that I was sick because I had never been bedridden. The herpes zoster I got recently is the only illness that has put me down so far. I have lost some kilos. I now understand what it means to have HIV but when you are moving.

**Esther: What else do you consider important besides the medicine.**

I don’t know. What shall we do about that medicine? **Esther:** Which, Dapsone? Betina: Ummm, I feel it is overburdening me *musawo*. **Esther:** but Bwire told me that he is the one who buys it. **Betina:** sometimes he fails, do you call issues of men food. **Esther:** so what do you do when he fails to buy your medicine? When I have money, I inject in myself. But also the boy, I go and tell him. **Esther:** How do you manage to open up to such people? Many of your colleagues are not as open. **Betina:** You see that. If you go to the health facility, it is not good to hide, *musawo*. Tell them what you are so that they can know the medicine you use. Because I react to some medicines, I may hide then they give me medicine that is going to harm me. I show you, I move with my book. Even if you are the one, *musawo*, I show you. People always wonder about me, I don’t hide, I tell them how I am, sometimes they refuse to believe me, when I am not thin, they disagree for real, but I tell them the truth. **Esther: where do you get such courage?** (raises voice) even if I hide, the illness is going to kill me. But you see, if you have a friend, whom you call and talk, then they tell you, you should do this and this. Those people who hide want to kill their friends, but that is not my mission. How will I defend myself before God? Everybody knows how they will face their crime. **Esther:** Some PLHIV justify their actions by indicating that the person who infected them did not think twice about doing it**. Betina:** I can’t, I can’t, you see that, you would be reducing your life span. **Esther: How do you befriend the health workers to the extent that you can send them for medicine?** My friend, I tell them. Then they say they have friends, sometimes they call them and ask. I tell them that I have failed to find the medicine. **Betina:** Where my niece is enrolled they have associations which visit them at home. The medicine is taken to their homes. **Esther:** that arrangement is called home based care. Very few people have an opportunity to benefit from such arrangements. **Betina:** No, apart from me who takes Dapsone. But when it is there, they give me. There is a time my husband went to pick his medicine and found it there; they gave him to bring for me. **Esther: How did you convince the health worker to give him the medicine when you had not come?** The nurse who dispenses medicine is our very good friend. **Esther:** how did you manage to befriend her she did not seem like a very easy person to approach? **Betina:** (laughs) she is easy, you see my husband befriended her. She is his very good friend. His very good friend! **Esther: was she in charge of the ART clinic by the time Bwire got enrolled?** Yes.

**Esther: Anything else you consider important?** Things to eat. The truth is we get little things to eat. They tell us that if you have enough to eat, we should eat 5 times a day. But sometimes we eat once a day, when we don’t have. Sometimes, he swallows medicine without anything to eat. **Esther: why?** When the food is not there. **Esther:** But you said you have gardens of food. **Betina:** the yields are not that good. **Esther:** i thought it was impossible for someone to swallow ARVs on an empty stomach. **Betina:** it is true; he sometimes swallows his without any food. They gave us the jerry cans a long time ago, they have never given us others. It is now 6 years since we received them. **Esther: Is the jerry can useful?** Yes we store our drinking water there. But we don’t use the tablet (water guard). We used to, but abandoned it because it smells and causes nausea. We just boil our water. **Esther:** How come you don’t fear using the jerry cans, i understand other patients abandon them. **Betina:** for us we are open. You say what you are, sometimes those who help come through such openness. Like what happened with our landlord. My husband helps him to collect *busuulu* (ground rent) from other tenants. He had come to collect his money when he saw our white jerry cans drying in the compound. His comment was, these are very clean (this is spoken with an element of sarcasm). My husband immediately told him that him and I were sick, and got the jerry cans from the health facility to help us with storing water. They talked a bit about stigma. He later told him that his wife worked in a big public health facility as a nurse. He saw this as opportunity to ask him to talk to the wife about getting Dapsone for me. He promised to help. And may be when he returns he will bring me medicine.

**Esther: You have so far told me that medicine and food are important things to have, anything else?**

Money (silence). **Esther: How about caretakers?** They are not bad. **Esther who has been helping you when the herpes zoster put you down?** Sometimes it was my sister in- law and Bwire himself. **Esther; does Bwire know how to cook?** (whispers) why wouldn’t he know? He has been waking up early to cook, make passion juice and leave it there, then he would go to the garden. (She raises the voice again) When he would return he would start preparing lunch.

**Ranking the resources**

**Medicine** – (says while laughing), *musawo*, medicine. (continues laughing for a while). **Esther: what next?**

**Money**, we do not have support. **Esther: which kind of support do you want?** What we can manage, for instance, if we got opportunity to rear livestock from which we can get some little money. **Esther; why aren’t you rearing any livestock?** What shall we rear? **Esther:** cows for instance. **Betina:** Where will the money to buy them come from? There is a time I heard about associations. I heard that some of them were given, goats, cows, pigs and chicken. But those associations never reached here. **Esther: Do you have any associations in this area?** No **Esther: But have you heard of any?** Silence... **Esther: how did you learn that others had got support?** They have associations. I hear they go for trainings and even sleep there. They would feed them and sometimes give them money. . We wanted to join an association led by a man called Suubi. I don’t think you know him. He mobilised us they told us to contribute 3000/= each. After they told us to contribute 20,000/=. We contributed money, 20,000/= each, we had failed to raise the required amount, then I lent them 50,000/=. They said they would give me 70,000/= but now I wonder about those people, because I think I may never get involved in associations again. After sometime, they told us that our request had not been accepted.

**Esther: How were you supposed to benefit?** I have forgotten that, since I did not go to school. My money got lost there. **Esther**: (prompts) He said that you mobilise money then... **Betina: (**picks the cue and adds) then we would form associations, those for mats... then we would get people to buy them. **Esther: Where does Suubi get medicine from?** He might not be on medicine. He is a health worker. **Esther: Was he mobilising you as a health worker or as one of them?** He always got involved like he was one of us, but it seems he wasn’t. **Esther: Does that mean there is no other association for PLHIV?** There are none. **Esther: How do you manage to know other PLHIV on medicine, because many of them hide?** Majority of those of us in this area know each other. I think my husband told you that we are now receiving the medicine through the outreach. Today we came here because I was still feeling pain around my waist due to herpes zoster. Those days when we used to come here , we would check if our days for refill coincided, and when they would coincide, to save on transport we had to board motorcycles in pairs, they would charge us 15,000/= , 7500/= each up to the tarmac (main high way). One person is charged 10000/=. It helped me to save money for the next trip. I don’t think I would have managed.

**Esther: how did you manage to make such arrangements?** We always found each other at the treatment centre and then we would always consult each other about our return dates. I know three people in my village who go to the same treatment centre. In another Village I know another three people. We used to consult each other. Most of these people have since taken up the idea of receiving refill through outreach. . All of us were given an opportunity to choose the convenient place for us. **Esther: How much does it cost her to reach the outreach centre?** We board cars enroute to Kampala and are charged 2000/= for a return journey. **Esther: How about by motorcycle?** It is expensive, they can ask for 10,000/=, Bodaboda in this area are very expensive. But one can ride a bicycle. Most patients ride bicycles. You see if you skip an appointment you are fined. Esther: but I haven’t seen anyone fined here . **Betina:** (dramatically) What? Let me first sit properly. **Esther (laughs) up to now, do they charge money, do you pay it from behind there (meaning not on the triage)?** You have to pay it. **Esther: how much is the fine?** We used to pay 17,000/=. Now me who cannot read those things, i have ever cried when things had failed, i had gone on a day that was not mine, my day had passed without my notice. Sometimes you forget. It was like this Thursday then I didn’t go then I went the following Thursday. **Esther: How did you notice that you had skipped your appointment?** I often get my book and ask those who can read to read the date for me. They told me that I had already skipped the appointment. It is a hassle; they disturb you at the triage but most of all at the pharmacy. To get medicine, you first pay the 17,000/=.  **Esther: what happens when you don’t have it?** (silence) ‘uumhu, a person who is arrested can’t fail to raise the required money . When you don’t get medicine, it would be a problem.

**Esther: Why did you rank medicine first and money second?** Medicine is life. We are always looking for where to find money. When you don’t have money, will you go to get medicine *musawo*? What will you use to travel? Now like me who buys medicine, it is necessary for one to have a job from which such money can be raised. **Esther; so how do you raise money?** Cultivation but we mainly grow food for consumption. Even if you grow food for sale how much money can you generate, there is no market here. A sack of sweet potatoes that is over stacked is only 20,000/=. What can it benefit you *musawo*? **Esther**: It would have to be a lot of food for one to benefit. **Betina:** Yes. How many gardens would you have cultivated? He!!’ **Esther: You mean you do nothing else?** I make mats during my leisure time but they are not for sale. **Esther: Why, you should start selling them?** There is no market. People in my area don’t buy mats. Mats are not bought there, except in the big town. One may make them and fail to transport them there. **Esther; why don’t you take them to your mother’s place, there may be a better market?** It is also a village and I need transport by taxi to the main road of 10000 and then 3000/= by bodaboda to reach my mother’s home. Sometimes you don’t have that much money and during the rainy season we spend much time in the garden, we have to dig to assure the children of food.

**Esther: Why did you rank food the third?** *Musawo*, can you eat, starting from today until the year ends, only sweet potatoes, without changing? **Esther**: You could buy posho. **Betina:** (laughs) money, besides maize doesn’t yield properly here.

In her ranking she never talked about caretakers. So I asked her if she considered them the fourth. She just kept quiet perhaps implying that she didn’t consider them very necessary. I tried to probe but she remained dodgy. I decided to ask about resources at the Health centre.

**Resources at the health centre**

**Medicine**- this needs to be there. Even if you find your own transport. When you inject money in yourself to travel, it is important you find the medicine there. Then you won’t feel much inconvenienced because you would know getting medicine is sure deal. I think they should not ask me for the 2000/= or give it back to me because I hardly get medicine. **Esther:** even without getting medicine you use the services of the health facility, seen the doctor, taken CD4 tests etc. **Betina: (**laughs heartily) I once asked the doctor and he told me the same thing. He further told me that I was lucky here I was charged only 2000/= but where they go (the doctor) they are charged 5000/= (continues laughing).

**Health workers**- they have to be there.

**Diagnostics**- they help us to know where we stand. **Esther: Do you know your CD4 count?** I always ask about them when I go for refill. They are in my file. **Esther: how many did you have the last time you went for refill?** I don’t remember. (I offered to read them for her from the book and told her they were always recorded there). **Esther: Did you get any problems this time round when you came on a day you were not scheduled to come?** No, they didn’t disturb me at all. I even took a CD4 test. They do not disturb us when we are sick.

**Ranking resources**

As long as the medicine and health workers are there I would be comfortable.

**Resources in the community**

(She kept silent for a while and then said she had failed to think of any. I told her I was going to suggest some and cited health facilities.)

**Betina:** It would be good if we could get medicine to treat *musujja* (malaria, fever) and other things from such health facilities. Things that don’t require us to go to the treatment centre.

**Esther**: How about friends? (speaks enthusiastically) my friend they are very important *musawo*. What they have done for me, achhii (expression) they have really cared. They have given me everything, sugar, money, they have given me. They have been coming to visit me when I was down. My place always had people. One gets 4000/= another 5000/=, yii (expression).

**Esther: How did they know you were sick?** They are told. One person gets to know that you are sick and tells another and the cycle continues, then they come. **Esther: Was it only your friends with HIV?** No, all my friends. I always tell my friends to go and test to know where they stand. It is always good to know one’s status. It is better to know and start medicine rather than waiting to get critically ill then they admit you, it is not good.

**Esther: Are there people you have advised to test who have heeded to your advice?** They are many. I went with a woman to the treatment centre in December. It had been my day for refill. I offered to go with this woman, each of us catered for their own transport. When we reached I talked to the counsellor who then helped the woman to test. She had been found negative. I always tell them, I never hide my status, and all of them know I am positive.

**Esther: Has any of your friends ever got herpes zoster?** Yes I have seen one, (lowers her voice) and even my husband got it, but I did not recognise it then. We went to a health worker who gave him a tube then he got fine. **Esther:** perhaps Bwire didn’t know it either. **Betina:** I am not sure. **Esther: Did he recognise your condition?** As if he did, but it was a fellow patient who had suffered from it who helped us confirm that that is what I was suffering from. The health workers in the clinics we visited were not sure. I got up in the morning when I was okay. I ate lunch but didn’t eat supper because I didn’t feel like. I felt heat in the stomach. I first got fever (musujja) and went to the HCII, I got coartem and finished the dose. I then got sharp pain around the waist, I slept badly that night. I felt the stomach go cold and could not feel anything in it. I failed to sleep and thought my intestines had got problems. In the morning, I spent the day badly off, I didn’t eat. He took me to a clinic. They measured and said the blood pressure was high, I had also felt it. I felt a lot of heat. It was a Thursday; they gave me a dose for pressure to swallow and told me to stop worrying. We went back home and I started on it. My daughter had got lost. I felt better on Friday, but on Saturday, it started bringing sores around the waist. I had told my husband that I felt sores and when he checked they were there. On Sunday Bwire saw a nurse who is a wife to the in charge of Health centre II, and told her everything. She told him to take me in the evening after sunset so that she would give me an injection. Bwire had told her about mycondition while they were at church. Bwire took me. When she saw it she said it was herpes zoster and prescribed 5 injections. She gave me one injection and a tube. The tablets she gave me were the panadol that was sealed. I started swallowing them. Then on Monday I went back for another injection at night, but there had been no improvement. I started feeling like thorns were piercing me. I went back on Tuesday, Wednesday but there was no improvement, it was instead spreading. The nurse recommended that we go to the treatment centre. We had had plans to go to the treatment centre the following day (Thursday) but I got worse at night. We woke up at 3:00 am when I was complaining that I had started feeling like something was piercing my heart. (She opened her gomesi and showed me the scars that stretched across the whole back. She told me that whole stretch got paralysed. Even when she pinches she feels nothing around that part. She pinched and told me she felt nothing. She still felt a lot of itching on that part).

**Esther: How are you managing the itching?** I bought a tube from vendors of medicine who go through the village. They told me it was Chinese medicine and that it works. There is another woman these people bring medicine for. Her legs got swollen and she could no longer walk. They always come with a car and park in the trading centre and then move through the village marketing their products from home to home. The Chinese medicine has not worked for me, the scars are still itching. They had told me it would help relieve the itching. The first medicine they gave me at the treatment centre got finished but they have added me more.

**Esther: Have you disclosed to other relatives besides your mother and elder sister?** Yes. They are there. I have my younger sibling, our last born, she stays with me. She helps me a lot. My relatives care about me.

**Commonest illness**

**Cough and flu**- these are taken to the public health facility. When the treatment obtained from the public health facility fails, we go to clinics. Sometimes the public health facility has no medicine, we are told to buy and when we don’t have money we stay without treatment or cook herbal remedies made out of a mixture of guarva leaves, mango tree branches, and thorns called *kibeere*. These are mixed and cooked. They relieve the cough. They are boiled daily and the dosage ranges from a spoon to two twice a day. I learnt this from my mother.

**Malaria** is rare (although when I checked her book it indicated she got malaria frequently last year). We used to get coartem from the treatment centre but it is no longer available. Panadol also itches me. I wonder what is wrong with my body. That’s why I fear Rvs. I am likely to react to them. I am already worried about them. My husband did not get any slight side effect. I have heard of several side effects, some of them look burnt, others turn yellow, nausea. There is one who wanted to leave them because she was over vomiting. I told her to persist and eventually she got better. They tell us to go back to the health facility in case of serious signs like yellowing and burns. I will camp at my mother’s place near the facility when I start medicine, my place is far.

**Esther: which illness do you fear most?** Cough and flu, they mistreat me more than fever. (I kept referring to cough instead of the *kiganda* word. She often asked me what cough meant). The dust is too much, digging also sometimes makes my chest pain. I don’t dig for money. I failed to do that because I have no energy. I cannot expend the energy I would have concentrated at my home on other people’s gardens. I do not expect to work much because I was told that I need 6 months to get real relief and 2 years for total healing. My waist is feeling heavy, people are digging, but me I wake up to just sit. **Esther:** But the dry season is on. **Betina:** People are digging, they are scraping such that when the rains come they will just smoothen and plant, my husband is digging alone.

**Esther: who told you that the scars would take that long to heal?** My colleagues are the ones who tell me that. One who was afflicted by herpes zoster says when it starts itching, it is horrible and when the day one got it come, it itches and pains a lot. The other person who got herpes zoster got it around the neck , the scars are big. **Esther:** how is your colleague managing the scars, the pain and itching? I do not know, but most likely nothing, because they would have told me if there was anything they were using.

**Esther: Did you ask the person who had got herpes zoster before for any advice?** (Her answer is not definite) Yii, they have really visited me. **Esther: are there are any other medicines you use?** We go to public health facilities in case of any illness, whether flu, cough, malaria etc. May be for flu that is mild, we may pick lemon from trees and suck. **Esther: some PLHIV use herbal remedies like honey, how about you?** Where does the money to buy honey come from? For malaria (fever) we go to the public health facilities. When coartem is not there, we buy. Private clinics have it although it is expensive. A dose is about 8000/=.

(I checked her book to check her last recorded CD4 as I had promised. As we perused the book together, I could see that on several occasions last year, she had malaria and cough as well as something to do with the skin. I asked her what had happened to her skin then she said it had been itching. The records were of the treatment centre but also other facilities which she said were public health facilities; they are the ones who record in these books, private people normally have other sheets they record on. I noticed that on several occasions she had to buy some of the prescribed drugs including Dapsone. She told me they had bought all of it. As I flipped one page after another, she looked on curiously. We were silent for a while as we scrutinised one page after another. To break the silence I asked her if she was using any herbal concoctions and that I knew they are always told not to use it. She said she could not use it because, it reduces CD4. She had been taught well about the medicine from the treatment centre. She said that some people were using it. One counsellor whose name she could not remember had told her that she had an aunt who used to cook the herbal medicine. The counsellor had suspended tablets for a while and tried the herbs, but by the time she went back to the hospital to test her CD4 it had reduced tremendously. I asked if they were told it was okay to use herbs for other illnesses. She said as long as it was not medicine for treating HIV it was okay. (The education sessions I attended had not been very clear on this. They simply barred them from using herbs (what they call medicine in jerry cans), but from what I gathered through interacting with PLHIV it seems it is mainly medicine for HIV that is packed in jerry cans). The CD4 I saw was for early 2015.
