# Supplementary material for: Social capital and resilience among people living on antiretroviral therapy in resource-poor Uganda
Source: PLoS One. 2018 Jun 11;13(6):e0197979. doi: 10.1371/journal.pone.0197979 (PMC5995438; doi:10.1371/journal.pone.0197979)
Supplement: S6 File — (DOC) [file pone.0197979.s007.doc]

**Client ID: 007**

**Name: Allen (Pseudonym)**

Status: **ART (CBV/N)**

**Section 1: Socio demographic characteristics**

Age:  **25 years**

Sex: **Female**

Marital status: **Married**

Highest education level attained: **S.3**

Main Source of livelihood: **She has no source of livelihood. She is a** **House wife**

Ethnicity: **Muganda**

Household size: **3. (She has not disclosed to the husband)**

**Esther: Did you disclose to your mother?** Yes she is aware. She is the one I registered as my treatment supporter. Sometimes I would leave the book behind with her, then she would go and pick the medicine and keep it home, then I would travel at will when I got time to pick it. As my registered treatment supporter it was easy for her to pick medicines on my behalf. She would just say that I am not feeling well then they would give it to her. They don’t disturb people who are registered as supporters as long as they go with the patient’s book.

Before I got pregnant with my daughter who is now 2 years, the first time I tested my CD4 it was about 199 cells, but when I checked again after 6 months it had risen to 700 cells. Everyone wondered how I had managed to raise my CD4 that much in a short time. The counsellors proposed that I should be given a slot as an expert patient. They wanted to know how I was taking the medicine, because they had never seen anyone who had made such gains in CD4 within 6 months. I asked one counsellor and a doctor who were both my friends if I could now conceive. They told me I could conceive and that my child would be fine. That is when I got pregnant with my baby girl of 2 years. **Esther: How did you prevent pregnancy?** I was abstaining. I refused all the men and didn’t want to sleep with any man any more. By the time I met my current husband I had recovered and looked good. I felt like I needed another child. I told him that I would only get married to him and produce if he accepted to visit my home and wed me in church. He accepted, so we got married in a church. I produced my baby girl a year later. My hope is that she is free from HIV. I have tested her at the recommended ages and the tests were negative. I am waiting for the next couple of months for her to turn 2 years to test the last time. **Esther: did you inform him about your status?** No. He is not aware. I only informed my mother and she and I concurred that it was not wise to inform him, he may run away.

**Esther: what is your CD4 count now?** I haven’t tested in a long time. When I go to the facility they confuse me. I ask for the paper to go and test but they confuse me, I am yet to test from there. **Esther: when did you start receiving medicine from here?** (Thinks for a while). I was still receiving it from my home district in southwestern Uganda the other year. I haven’t spent a long time with my husband. Going to the village to pick medicine was not difficult. I had some money because I continued working when I got married. I worked until the baby’s pregnancy was 8 months. I then moved to the village to deliver and spent there several months. By the time I came back, we were preparing to shift to our current place then I changed to this treatment centre. When I was still working I had my money and would not ask my husband for transport. I would raise the 15000/= or 12000 to go and pick my medicines and raise another to come back. On average I needed 30,000/= for a return trip every 3 months. It was not hard to mobilise it.

**Esther: Now that you are no longer working, how do you raise money in case you cannot ask your husband directly?** I always have money I make from making differences on money I am given to buy food and other things. **Esther: Where do you keep the medicine?** I keep it in the side/cupboard amidst plates and cups. He never checks the side board. What would he be looking for from there? When you spend time with somebody, you can tell where they usually touch and not. **Esther: what if you leave one day, then he gets visitors and checks the side board for cups or plates then lands on it?** It cannot happen. When I am travelling, I remove it from the side board, put it in my bag and move with it. I keep it in black polythene so when I am going away for sometime I just grab the polythene, put it in my bag and move with all of it. **Esther: have you ever failed to swallow it because he was around?** (laughs) yes, there are times he decides to come back earlier than expected. I take the morning pill at 8:00am and the evening one at 8:00pm. He is usually at work at those times. He loves watching films. Sometimes he returns earlier and watches films in the living room, where the cupboard is, for hours. It becomes difficult for me to get the medicines. I am usually forced to skip the dose to avoid creating trouble for myself. But it does not happen all the time. It is rare. I manage to take my medicine most of the time.

**Esther: do you plan to get more children?** I don’t know but I wanted to stop on the baby in the womb. I asked the big nurse about the possibilities of stopping to deliver. She advised me to ask for caesarean so that my tubes are cut in the process. When I thought about it, I decided it wasn’t a good idea. A knife with the little life I have. How many people ask for it? It weakens the body. You cannot carry heavy weights because you are cautious about the scar, you cut down work, you can’t do your work. I don’t want the knife, adding a knife to my life, when I produce normally?

**Esther:** It is difficult for a married woman to get tubal ligation without the husband signing. If you haven’t talked about it and agreed it would be difficult for the surgeons to perform the procedure. **Allen:** My husband has no problem if you tell him that you no longer want to produce. But I also can’t manage it. They tell us there are contraceptive tablets that are herbal, but we asked about them then they told us they cost 200,000/=. There are ‘sengas’ (paternal aunt) on T.V. They say they can tie you and release you when you feel like producing. I hear you don’t get any side effects.’ **Esther:** how do they do it? In a ‘*kiganda*’ (traditional way). These modern family planning methods have side effects, you can feel weak.

**Esther: why don’t you try a coil?** I heard that it also has side effects. They told me that the implants also have side effects. I saw a woman at the health facility with my own eyes, who had come to have them removed. She looked bad and said they were the cause of her woes. She said they should remove them, even if they needed money she would pay. They told her to pay 50,000/=. The health workers said inserting them is free but removing them is 50,000/= (laughs), these health workers also (a negative expression). The woman was told to wait for the medical persons who could remove them, but she had to pay some money. Removing them before their time is expensive, they work for 5 years. The woman was thin, she looked sick like this illness (HIV) I told her is it the one (implant)? Then she said it is the one, these things of the hand are becoming unbearable. I don’t eat, I don’t drink, I am not myself, I am dizzy. I feared them, they are the ones I wanted to opt for.

**Esther: Try a coil. It doesn’t have much side effects but is not good for those who get frequent infections like candidiasis because they normally remove the coil in case of infections.** Eeh, i have problems with candidiasis. It is the first disease I am going to treat after delivery. Even when I treat, it still persists. At first I thought I was getting it because of sharing bathrooms with several people where we were renting. I am now in my own home but still get it. **Esther: how do you spend your day?** I don’t have much to do. I wake up and cook food, clean and other domestic work. I also fetch water from the well, we have no piped water supply in the area. Sometimes I work on my maize garden which I planted in the section of our land that is yet to be developed.

I hardly fall sick these days, but when I get things like cough, I buy tablets or capsules and swallow. There are several clinics in the trading centre, the same place where I buy food.

**Resources considered necessary at home**

**Food**- What I consider most important is food. Because I am pregnant I eat mangoes, pawpaws, avocado and pineapple are what I eat often. Even milk. I also drink milk. Every night I have to drink milk. Things like fish. These are the things I eat often.

**Esther: Did you suffer any side effects from the medicine you are swallowing?** Only when I had just started. I used to feel noise in the head and also became anaemic. I got 3 blood transfusions to get better. They told me to bring someone to donate blood so that I would be given, but I showed them slips I had been given when I used to donate blood during my teenage years. My blood group is O. At that time they changed me to the regimen I am currently taking.

**Esther: Is your husband’s source of livelihood only bodaboda riding?**  Yes. **Esther: he must be focused. Most people in that business are known to splash their money around.** For him he saves his money. **Esther: did you test for HIV before getting wedded?** No, we did not. He even never said a word about it. But I tested before the wedding and the results indicated I was negative. **Esther: Did you test before getting involved with your first born’s father?** I was tested when I was pregnant and found negative. But I was perturbed when I had just weaned the boy and got fever then they tested again and told me I was positive. I wonder why they had not detected the virus before. **Esther: perhaps you were newly infected by the time you tested and that’s why the virus hadn’t been detected. Were you tested during the 2^nd^ born’s pregnancy?** I was already on medicine so they didn’t test me. I told them I was on medicine. But I have ever tested during the mass testing exercises that were brought to my village and found negative. (laughs) I told the ‘*musawo* that I had tested and was negative, should I stop taking the medicine. The *musawo* said it happens and told me I would relapse if I stopped taking the medicine so I should continue.

**Esther: Are you aware of the status of the father of your first born?** I asked him then he told me he tested and was okay, but I think he could have lied. I have just come back from there. I had gone to check on my son. I asked him again then he said he was not sick.

**Esther: perhaps you got involved with someone when you left the first man.** No I didn’t. I left the father of my first born, not because of any other reason but because he refused to get introduced to my relatives. I left him when I went home to deliver and told him that I would not go back unless he came to pick me, then the bride wealth is set and he pays it. He had instead got angry and told me he had no money. I told him that I would not go back. That is the time I got sick. I first fell sick and became very thin and a shadow of my old self. My mother and other relatives took me to church, but I told them to take me for an HIV test. When they tested me they said I was sick. I have no problems with the medicine. I get food, I eat whatever I desire, apart from when I don’t feel like eating. **Esther: isn’t your husband stressing you with late coming or anything else?** Even if he comes back at 9:00 p.m or 10:00 pm, I get occupied by watching T.V, I watch my films, what would I want? When I got pregnant, the feelings disappeared so I don’t long for him. (She says while laughing) Even if he remains there. I only need him because he comes to give me money.

**(Both of us laughed and continued laughing for a while. I noted that not all women were that lucky).**

**Allen:** Mine doesn’t disturb me. I cannot feed my child on water. She is just small; I produced her when she was tiny. But I give her everything, blue band (margarine) is always there. I mix it in her food, rice, irish potatoes. That is how I cook for her. She drinks milk, I mix it with her porridge. It is always there in the flask.

**(I touched Maria who was seated next to her mother but playing with sand, and said, ‘She is still small but looks good.’ I noticed she had a rash on her skin and asked the mother what it was)**

**Allen:** It seems like mosquitoes. They are many in our area, when they bite her she pinches the sores. We sleep with her but the mosquito net has holes so mosquitoes get to us. I am planning to buy her a bed so that the baby takes her place. When I deliver she will start sleeping alone. **Esther: Did you breast feed her?** I did, until she was 9 moths then the ‘*basawo*’ told me to wean her that I would infect her. At that time she had teeth. I went to my mother’s home for a month, and while there I weaned the baby. By the time I came back the baby was used to taking other foods. When my husband asked me why the baby was not breastfeeding I told him she had refused, so he started buying her food.

**Esther: why did you have to do that?** It would have been impossible for my husband to understand why I had to wean the baby. He would have started suspecting something. The baby would have cried terribly for the first two days. My breasts swelled, I had to squeeze the milk out with a warm cloth. I got fever and swallowed tablets. My chest was paining, I couldn’t do anything, including bending, it is my mother who used to cook. I was either sleeping or sitting all day. I could not have weaned the baby from my home, my husband would have smelt a rat. **Esther: your mother seems really supportive. Allen:** (It’s my mother who cares for me. When I fell sick she used to give me whatever i asked for, and my sisters used to send me things to eat. When I was sick, I never liked eating beans, I only wanted meat and fish (laughs) they are the ones I used to eat. I used to eat pineapples a lot; they used to send them to me. **Esther: do you have any plans to work again?** I want to be self employed. Nobody can employ me with children. Even if you ask for a job, they will tell you you have children. I don’t want to be ruled over now. Getting mad at me, you have not done this and that. I wake up early at my time 8:00 am, then I wash a few clothes, go to fetch water from the well, then cook and eat, and that’s it. My daughter goes in the neighbourhood and plays. There are no problems there and it is quiet. Once in a while I move and go to converse with my friends. But when you have electricity, you spend time on soaps, you don’t get time to move around. Some of my friends come to my home in the evening to watch soaps. **Esther: Are there any people who pick medicine with you that you keep in touch with?** There are none. But I have ever seen one of the counsellors in my neighbourhood. I talk to other patients when we are at the clinic, but we don’t visit or call each other. I don’t want to get too close to people that may cause me problems. **Esther: Which problems?** What if they inform my husband? **Esther: you mean you have never exchanged phone numbers with someone you met here?**

We exchanged numbers, that day, I think they had brought some little money, I think it had accumulated, then they decided to give us some, they gave us 3000/= each, they wrote everyone’s phone number, and also looked for the chairman, but I have not seen, they have never called us. It looks like they were not giving the chairman money, because they have to give you money then you call your members. I also don’t understand them. And when I went back I didn’t find even the chairman. I also got confused. I asked around and said the meeting we made for our association, they told me there was no association.

**Esther: did they communicate that they were going to form an association?** They had said, the association, it was an association for pregnant women and PLHIV, that is how it was. We had said that we would also save some little money, but no one seems to be concerned about it. **Esther: whose idea was it?** The one who brought it was the tall man, the counsellor. He was the one who brought it but when I went back I found when there was nothing). **Esther: what did he say exactly?** He told us that pregnant women and PLHIV there was support coming. Write your phone numbers. Write your addresses, things like that. We wrote everything. That day we sat, you give us 3000/=, but we have not sat again, we have never sat again. He has never talked about it again, it stopped there. Things of that place are difficult, because even mosquito nets are sent when they are ours, but they tell you they want 3000/=, they want this much. But I believe that they are meant for us for free. But giving it to you, I have never got any. There is nothing I have ever got from the treatment centre, there is nothing for free. **Esther: even a jerry can?** I have never got any. **Esther: I think you should tell them that you didn’t get. Allen:** I have never got anything, and going there then you give 500/= which they say is for cleaning, what. **Esther: when did you start paying the 500/=?** Every time you go. You pay from the table outside (the triage)). **Esther: but I never see any of you pay. Allen:** do you stay outside? Sometimes patients refuse to pay at the triage, but you pay from inside, to the nurse who delivered a baby. She knows, she personally asks for the 500/=. Unless you lie that you don’t have it, but you see that it is embarrassing. But there are those who say aaa (expression indicating, No) I don’t have it, then they refuse and they don’t give it to them. As for me, I have never refused to pay it, I always pay it, but mum, there I tell them, counsellor give me a mosquito net, then they become dodgy, I am still busy, what, then I discern that they actually want money (laughs).

**Challenges in obtaining resources**

I don’t have any problem. May be when a person comes here, this place is difficult. You stand. It is the one that encroaches on your wellbeing it seems. You delay, then another person finds you there, for me I come as early as 7:00 O’clock, I am normally there by 7:00 a.m. I am normally about the 10^th^ on the register when I am very late. But a person comes when they are the 30^th^, then they pass you and get medicine. Then you ask yourself, what is this? It is because they are known, I think. I feel angry; those are the things that make me angry. I don’t want a person to bypass me when I came earlier than you, yet I have been patient. Those are the things that disturb me, but I have no other problem.

**Esther: how supportive are your friends?** I have no friends, its friends who would have cared for you but when they learn that you are sick they forsake you. I see many who are forsaken by friends. I see them avoiding you. Now if you don’t have any source of livelihood, and this illness weakens you, then you will be there without anything to do, you are there seated, yet you want to eat!! The illness requires eating! Especially when you begin medicine, the illness needs eating, and you want to eat good things, you would want to fry!! Boiled food would be smelling bad to you. You want fried things, you don’t have money, you don’t work, there is nobody who can offer you work when you are looking bad (not presentable). You see, they would be ostracising you. You would also be fearing to go and ask for a job, you have no energy, you have no energy to walk. Now if you have no caretaker, in that situation the illness will really torture you. Unless you have someone who takes care of you, a person who knows you are sick and cares for you. That is how it is. If you have no things to eat you cannot manage. Yet they require money because you would be desiring to eat nice sauce. I am not saying you will go somewhere and cultivate them. You have to go and pay at the butcher, what, everything requires money. PLHIV really love fish and meat, it is really delicious for them. But when you get better, you start eating everything. When I was ill I used to eat them alot. I never wanted them to bring me anything else except fish and meat. But now I eat beans, ground nuts. I eat everything now. Even leafy greens, I pick and eat and mix it with *Katunkuma* (bitter green vegetables). For this illness, even leafy greens have no problem, they are actually good to eat, but you would not be wanting them. Fish, chicken, meat, eggs, milk, those are the things you want. That is why you see people sad/ frustrated, you want tea, bread, it is not there, uuhm (expression), that situation. You want bananas too, you find them delicious. I used to eat, they would get for me bunches of sweet bananas, then they would hang them up in the kitchen. I fell sick from the village. I was the only one eating and finishing the bananas, nobody else would touch them (laughs).

**Esther: your relatives take good care of you, but other people are not so lucky with relatives)** Yes, there are people who converse and say that they fell sick, and went to their mother. Their mother told them to go back where they picked the illness from for care. My mother chased me and I suffered with the illness. But for me my friend my mother is the one who took care of me. She used her personal money. She gets her money from the sales of harvest. Sometimes when you would tell mum that you want chicken and the chicken is there, she would slaughter then we eat. I would eat, then she keeps some and says it is for the patient. It is not that she will slaughter for everybody to eat, she would slaughter it for you until you feel the desire for it has diminished. When I visit the people who know and my mother, what I want is what they give me. That’s why I love delivering from the village because I know I have no capacity but I do, you don’t have energy, but you have. My mother is already calling every now and then to ask me to go the village and always asks who will take care of me. My husband says I should stay. I produced my second baby from this way (not from the village).

**Esther: Have you disclosed to some friends)** No. Telling friends who are near here will just jeopardise your relationship. When the friend taunts you, you will sleep badly. You may be taunted before the whole village. I don’t have any friend. I haven’t told my husband, how will I trust a friend? What would come first, trusting a friend or your husband? If I could fail to trust him, I refused to tell him. The only person I trust is my mother. If she was to forsake me, she would have forsaken me at the time I fell sick. By the time I was blood transfused I couldn’t get up, but she would do everything for me. It is good to swallow medicine in time but when you have things to eat and drink. When you don’t have things to eat, drink, the medicine will drain you, you just see yourself wasting away.

The baby was urinating on herself I thanked her for the interview then she took the baby to ease herself.
