# Supplementary material for: Social capital and resilience among people living on antiretroviral therapy in resource-poor Uganda
Source: PLoS One. 2018 Jun 11;13(6):e0197979. doi: 10.1371/journal.pone.0197979 (PMC5995438; doi:10.1371/journal.pone.0197979)
Supplement: S7 File — (DOC) [file pone.0197979.s008.doc]

**Client ID: 007**

**Name: Mutetsi (Pseudonym)**

Status: **ART (3TC/TDF/EFV)**

**Section 1: Socio demographic characteristics**

Age:  **22 years**

Sex:  **Female**

Marital status: **Cohabiting**

Highest education level attained: P.4

Main Source of livelihood: **She has no source of livelihood. She is a** **House wife.**

Ethnicity: **Munyankole**

Household size: **3**

**History of Meeting**

The first time I met Mutetsi, I hadn’t paid much attention to her. It was clinic day and I was helping with the distribution of medicine. When she came to the window that day she had been carrying Nakato. Nakato was extremely thin and her skin was peeling off. The only big thing on her body was the head. The expression on Mutetsi’’s face had been flat. I couldn’t really discern what was going on in her mind. But she didn’t look bad at all. I asked her about the baby’s health and why she had delayed to get her tested. She told me she had thought she was just small. Her twin sister was doing fine, so she couldn’t suspect anything. All I can remember is that when I left that day, the picture of the little baby was stuck in my head. I thought she was a beautiful little girl and felt sorry for her. I thought she must be feeling a lot of pain and wondered if she would recover.

The next time we met was two weeks later. This time I paid attention to them because when they emerged at the window, I was surprised that the baby had improved tremendously. Her skin was better and she looked a lot healthier. Her beauty was now more conspicuous. It had rained heavily that day. The baby was wearing several heavy clothes. While they were still waiting at the window, she started crying and the mother couldn’t somehow make her keep quiet. My motherly instincts pushed me to go and find out what could be wrong. I excused myself and went to talk to Mutetsi. She was holding the baby in one hand and a handbag full of baby clothes in the other. A flask and a cup placed upside down on its mouth stood on the floor beside her. I asked what was in the flask then she said it was Nakato’s tea; she had stopped breastfeeding because she didn’t have enough breast milk for both the girls. I suggested that she gives the girl tea but she said she had been vomiting everything since morning. She had the appetite but vomited everything. I noted that she could be hungry then she concurred. She told me the baby liked bananas. Fortunately, I had bought bananas in the morning from one of the counsellors who was vending them at the clinic. I got one of the bananas and gave them to Mutetsi. She picked their medicines, left the window and sat on one of the empty benches in the corridor. She peeled the banana and started feeding the girl. She nibbled at it and didn’t let go until it was about half. She seemed to be enjoying it. Her mother was attempting to feed her again when she vomited everything she had eaten. I asked Mutetsi what could be wrong but she said it was because of the cold. She changed the girl’s clothes and showed me several bed sheets folded in to a ball which she had spoilt earlier. She had only one dress and sweater left. She dressed her in these last clothes. The mother then used one of the bed sheets to clean the spoils from the bench which she placed in her bag after. She told me she had to go because soon the girl would have nothing to wear. The rain had subsided so she picked her things and left.

Thirty minutes later Mutetsi and her daughter were back, but this time accompanied by somebody else. This woman brought them straight to the dispensing room. She opened and entered and greeted the nurse casually. While Mutetsi and her daughter stood silently in the door way the woman started explaining that the girl had severe malaria and should be treated. She told the nurse that a drip of quinine would be better. She looked dehydrated. The nurse took them to see one of the medical officers. Mutetsi and the daughter came out first and sat on the bench. I joined them and asked what had happened and who the woman was. She told me the woman was a nurse who immunised children. She was the one who had suggested that she tests for HIV. She had brought her children of 1 and a half years for immunisation. The nurse weighed the twins and was perturbed that Nakato was too light for her age, but Mutetsi explained to her that the girl had never been big. They were almost the same weight at birth, but somehow Babirye grew faster. The nurse had asked her whether she had tested for HIV during pregnancy. She told her she hadn’t. That time there were no reagents and they told them to come back for the test with their husbands. She informed the father of the children but he was rude. He told her if she had HIV she should go and test for him he was fine. She decided to abandon the idea of going back for the test. That day the nurse had suggested that they test her for HIV. She accepted. She personally took her to the laboratory and talked to the laboratory assistant. They tested her blood and told her she was HIV positive. The nurse suggested that the children are also tested. She took them to the laboratory. They told her to pick their results after 2 weeks. When the 2 weeks elapsed she came back for the results. Those for Babirye were fine whilst Nakato’s were positive. The nurse brought them to the ART clinic and lobbied that they should be started on ART without any delay. That is how they had started ART.

The nurse came out of the medical officer’s room. She had a piece of paper on which the medical officer had scribbled the prescription. She told Mutetsi that Nakato had been admitted. It was the only way they were going to manage the malaria. Mutetsi objected and said she didn’t have any more clothes; the girl had vomited on all of them. Secondly, how would she tell her husband that the girl has been admitted when he had left her at home without any problem? She noted that he was difficult and may refuse to come and check on them or bring them anything. The nurse insisted the girl had to be admitted and offered to call her husband to inform him. We asked for her phone to check for his number then she said that she didn’t have one. Her husband had lost his and instead took away hers.

The nurse guided them to the inpatient ward which is housed in the same structure as the ART clinic, but is separated by a narrow corridor. I followed them to the admission ward. She showed them an empty bed, on which **Mutetsi** lay the last bed sheet she had and rested the baby. The nurse said she was going to call the nurse in charge and also bring them some food. She left and left me with them. The baby was cold and her hands were covered in goose bumps. I told her to get the baby a sweater but she said she had vomited on it. She was contemplating to leave the girl with the nurse she had come with and then run back and bring clothes. I realised she didn’t mention the nurse’s name, but I was able to find out that she was called Phoebe (pseudonym). She reiterated that her husband was a difficult man he may refuse to come to the health facility. In the process the nurse in charge came. She looked at the prescription and went in to her room. She came back minutes later with a cannular and drip. She noted that the canular was for adults and told Mutetsi that she had to buy some 2 canulars for babies. Mutetsi’s eyes became teary. She pulled out 2000/= from her bag and said that is all she had. She had used the money her husband had given her for upkeep for transport. She told me this is what she always did. The man never gets to know that they come to the ART clinic. Nurse Phoebe came back with some food. The other nurse explained that there were no canulars for children, so Mutetsi had to buy. Phoebe didn’t say anything and was perhaps thinking of where to get canulars from. Seeing Mutetsi’s despair, I offered to buy the canulars. I went back to the dispensing room, picked money and came back with 10000/=. I also came with one of the expert patients who had offered to go and buy. Phoebe directed her to a pharmacy which she said had good prices. She told her to buy two canulars and some quinine. She went. Meanwhile, the nurse in charge of admissions examined Nakato’s hand and decided to try the canular for adults. It worked. By the time the expert patient came back the drip was already on. Phoebe decided that the canulars are taken back and in exchange more doses of quinine are brought. The expert patient told her they may not accept, so she decided to call the attendant of the pharmacy directly. She was told that we would have to add some money. When she told me I handed them the balance they had given me from the earlier purchase. The counsellor went and came back with the quinine. By now Nakato was crying hysterically and her mother was struggling to calm her down. I suggested that she starts eating and also feeds her. She accepted then I handed her the plate of food brought by Phoebe. Both of them started eating. That is how Nakato kept quiet. I moved out a bit and met Phoebe in the corridor talking to the nurse in charge. She asked me how I had got to know Mutetsi and her daughter then I told her we had met here. I was a student on PhD research. She thanked me for helping and said, ‘She is such a beautiful girl. If you see her twin you will know the potential she has. Unfortunately, her father is funny.’

I asked how she had linked up with the two because they had told me they were going home when we parted at the ART clinic. She said they had taken Nakato to greet her as they do always.

‘They never come here and don’t look for me. Even when I am at home they come. They know my home. I was excited to see the girl. She seemed happy and smiled at me all the time. She never fails to smile when she sees me. I was playing with her then she vomited all of a sudden. Her mother told me that she had vomited since morning because of the cold weather. I examined her closely and noticed she was dehydrated. I decided to take her to the laboratory for a B/S (malaria test). It turned out she had malaria parasites plus plus. She would have died because she had no temperature. Her mother was just going to keep her home.’

We both went back in. Mutetsi reminded her to call her husband. She gave her the number then she dialled and called. He picked. Mutetsi introduced herself as a nurse and told him that Nakato had been admitted with malaria, they needed clothes and other things from home. He was surprised that the girl was ill, but before he could say more the call dropped. Phoebe said she had to go, but would check on them later. I stayed with them for a while. I wanted to follow up this case closely but unfortunately it was a Friday and the following day Saturday I was engaged and so couldn’t come back. The admission was scheduled to last 3 days. I decided to ask her for direction to her home and explained that I would visit them at a later date if it was okay with her. She said there was no problem and went ahead to direct me. I wrote the direction down as she talked. I told her it was late and I had to go. I handed her 5000/= to help her in case her husband didn’t turn up. She thanked me and asked for my phone to make a call to her husband. I asked her to mention the number which I dialled. He picked. She told him the girl was cold due to lack of enough clothes, he should help and bring some as soon as possible. I could hear him saying he was coming soon. She handed me the phone back then I left.

I didn’t see Mutetsi again until I visited her home over a month later on January 22^nd^ 2016. Getting there had been tough. The place was slummy, so getting the direction clearly was difficult. Whilst riding on a *bodaboda* (motorcycle taxi) we stopped several people to ask them if they knew the place but the majority told us they were new in the area. After several tries, we eventually found someone who knew the house she had used as the significant feature for direction. We slopped there and didn’t take long to find it. I sat in the sofa. Behind them was a curtain that separated the bed from the sitting room. On my left were saucepans, jerry cans and other household items. Behind the door lay several fingers of matooke that were scattered on the floor. She told me she was planning to cook, but I thought it was very late, past 2:00 O’clock. She told me she had just returned from fetching water from the protected spring that is why she was late. I complained that Nakato was naked, yet the weather wasn’t the best. She got her some clothes and dressed her up. She sat in the sofa across then we started talking. My first question focused on what had transpired when I left Nakato admitted at the treatment centre.

**Illness Narrative**

She said her husband had come later that day and brought them money for using. They spent 3 days in the hospital. He passed by everyday and left them with some little money (*obusente*) to buy food.

**Esther: didn’t he over react about the baby’s illness?** I connived with the nurse in charge with the help of nurse Phoebe to persuade my husband to test. They told him that in order for them to know what was disturbing the child they had to test him for HIV and if he refused they would not discharge us. But he refused and made a scene saying that he cannot test. He said if they discharge us let them do, but if they can’t because he has refused to test they can retain us (laughs) then he left.

(**I also joined in the laughter and asked why she thought he never wanted to test)**

**Mutetsi:** I don’t know, (expression) I don’t understand things. He refused. When I was pregnant I told him, at the dispensary they told us as if their machines had got issues. They told us to go and find health facilities and test to know our status. When I came here, I told him. He told me, who told you I am sick? Until the time for delivery came. At the time we delivered the machines had been brought back. **Esther: Did you get any challenges at the health facility the day I left you in the ward?**

No, we didn’t get any problems. **Esther: was the medicine we bought enough for the whole treatment?** The medicine used to get finished. *Musawo* (Phoebe) would buy. You know she also had a persistent cough. She took her for an x-ray, then she paid the money. She fought the cough, because she bought expensive medicine which healed it. When I tell this one (her husband), aaah (expression) he is not interested in treating the child. **Esther: But he came to visit you at the health centre, didn’t he notice that you were buying the medicine?** We told him but my friend you think he cares. He tells you one thing I don’t have money. Even when they write for you medicine and you come back and tell him, they wrote for me medicine here, won’t you buy it? I don’t have money. One day I got mad, I wanted to abandon the child with him and leave. When I shared with that musawo, she told me don’t do it, don’t do it to abandon Nakato. I felt I was fed up. Right now I am looking for work, but they have refused to give me with a child. Even if I get a job that is domestic I will do it. But they have so far refused to give them to me. I don’t know. **Esther: maybe you should find someone to leave the child with.** Ha, they would require money. **Esther: can’t your husband pay such a person?** That one, he cannot. **Esther: how about getting somebody and paying them from what you earn?** There is one I had seen but she asked for 20,000/=, I looked into it, 20,000/= when the job I was going to do was 30,000/=, I would not be benefitting.

**Esther: maybe you should target a job of at least 50,000/= then you will be able to afford leaving the girl with someone. Mutetsi:** Even if i get one that requires me to stay there full time, I would do it. **Esther: How about our husband? T**hat man is big headed!! A man who cannot take care of a child. You see her (points at Nakato) I think she would have regained by now, but she is on water which serves as tea. **Esther: what?** (she continues) water. The flask is there, it is water in it. She stopped taking milk long ago. May be on the day we go to the health facility the *musawo* gets me some little money, because she knows all my issues. She tells me now get this money and buy Nakato milk. When the money gets finished, she reverts to her water. **Esther: does he leave any money for upkeep?** He leaves very little. He may leave you with 3000/=. 3000/= from which you have to buy milk, food. Apart from the other day when he escorted a friend for a burial, then his friend gave him that little food (she points to the fingers of matooke behind the door). It is the one on which we have been surviving. There is a time he dumps for you 500/=. **Esther: what?** I swear in the name of God in heaven. A time reached then I felt so fed up and told his mother. His mother told me, what should I do for you? What I will do, she said she was going to get me transport and give me my children so that I could go back to my parent’s home (Both of us laughed), She continued, ‘Now tell me.’ **(The girl I had found carrying Nakato screams something which I didn’t hear. Mutetsi tells her to help put her food on the charcoal stove and then continued from where she had stopped)** another time I went and told her, but why does your son come back towards morning. She told me, that son of mine, but don’t tell him, seems to have another wife (laughs heartily).

**Esther: he comes back at dawn?** She said there is a time he comes back at 2:00, 3:00 and 4:00 a.m. But it is not that he is working. The next day there is no money for food. He dumps for you 500/= and goes. **Esther: Nakato’s problem seems to be poor feeding. Mutetsi:** yes, it is the poor care. That is why I am looking for a job and leave him. I will go and labour for my child and ensure that she regains. **Esther: I think she has the potential to recover. She is a strong girl. Mutetsi:** Nakato would now be walking. **Esther: so did she get fine after the treatment at the health facility?** She got fine, but the cough had refused. They gave us medicine at the health facility but it refused. We were discharged but it still persisted. *Musawo* used to call to ask how the girl was doing. I told her that the cough had refused then she told me to take her back to the health centre. The nurse took Nakato back to the doctors who prescribed her another drug. The medicine they were prescribing was very expensive, costing 10,000/=, 8000/=, *musawo* would buy it, but the cough refused. Until the doctor wrote that we should take her for an x-ray. **Esther: Doesthe treatment centre have x-ray services?** No. *Musawo* took Nakato to a health facility in town. We went together. On the same day *musawo* bought Nakato a flask (shows me the flask).

**Esther: Nakato had a flask the last time we met). Mutetsi: (**laughs) those flasks didn’t have covers. (Laughs) the flask you saw at the ART clinic didn’t have an inner cover; I merely put the top cover so the tea was virtually exposed. When *musawo* noticed that Nakato had a problem with a flask, she bought her one. She took Nakato for an x-ray, they asked her for 35,000/= which she paid. They told her the girl had pneumonia, I think it was the cause of the cough. She started her on medicine. That is how Nakato’s cough healed. The children are due for immunisation. *Musawo* told me to take them back on 16/02/2016. That is what she wrote. I told him then he said, I don’t have money. I am now here just looking at him. Would you be bad if you left that one and went to work? **Esther: No, what does he expect the children to eat?** He doesn’t care. He throws for me 500/= and then comes back at night with chapati and beans and gives her (baby). Nakato pushes it away. This girl you see is picky on food. She doesn’t like posho, chapati.

**Esther: so where do you get money to go for refill?** I use what he provides for upkeep for that day. At least I know *musawo* buys us food when we come. Sometimes we walk. We use a shortcut and go. **Esther: it is far with that baby on the back?** Yes, you would have nothing to do about it when the medicine gets finished. You walk. Other times, I call *musawo* and inform her. She usually sends us mobile money, then we come. **Esther: where do you keep the medicine?** In my hand bag. He never touches it. The good thing he doesn’t check things around. He doesn’t even have the time to do so, he goes very early. I sometimes spend 2 weeks without seeing him. He finds us already asleep, then he wakes up early and goes. By the time I wake up, he has already gone. **Esther: does that mean he has his own key?** No, I just close the door, so he is able to open. **Esther: thieves may steal your things one day, given the time he returns. Mutetsi:** I complained once and told him that our place had many people on drugs, what if one entered to steal and found nothing valuable and then raped her. He had got angry, quarrelled and told me I wasn’t supposed to give him orders. He can come any time he wishes. I gave up on him.

**Esther: how much rent are you paying? Does he pay it in time?** Our rent is 70000/= per month. His payment pattern is erratic. The only thing is that the landlord is patient but sometimes he threatens to chase us. He gives him a month’s pay then he relaxes. Rent is due for two months but he is yet to pay.

**Esther: Do you manage to eat before swallowing the medicine?** I take one tablet at night. The food, whether it is there or not, we just survive. When you take the medicine without eating, it causes dizziness. You feel drunk at night (laughs). **Esther: how do you manage?** With or without food I swallow the medicine, because I can’t leave it. **Esther: You could at least borrow. Mutetsi:** From whom? **Esther: what is your relationship with the girl I found carrying Nakato? S**he is my sister. I brought her to help when I was pregnant but she ended up eloping with a man and left. She just comes to say hello once in a while. **Esther: Is she able to give you any support?** The person my sister got is a lumpen, she is equally struggling. She can’t offer much help apart from coming to say hi once in a while.

**Esther: are you able to get other essentials?** Soap. When he leaves you money for food you have to find a way of getting other things. I have spent 2 weeks without soap. But today he left me with 2000/= so I managed to buy some soap. It is the reason I have been fetching water since morning to be able to wash. When my husband finds his clothes dirty he quarrels. I always ask him whether I should just steal soap from the shop but he just keeps quiet. I haven’t washed yet; he is going to quarrel when he comes back. **Esther: He is not an easy man. Mutetsi:** He is difficult). (a moment of silence ensues, then she breaks it by saying) ‘Even if I get domestic work, I will do it to sustain my child.’

**(Nakato started crying. She’d been silent since we started the interview. She sat on and off her mother’s lap. I commented that Nakato was waiting for food. Mutetsi told me that sometimes she refuses the water, but after a while takes it again because she has nothing to do)**

**Esther: Does musawo always buy her milk when you go the dispensary?** Yes she gives me money. She may give me 5000/= or 10,000/=. She estimates that this may take her for several days if I buy her a cup every day. There is an old man in the neighbourhood who sells a cup at 500/=, that is where I go to buy the milk. When there is no sugar I buy a quarter kilogramme of sugar to put in her milk on the same money. Nakato doesn’t like plain water. I have to sweeten the water with sugar for her to take it.

**Esther:** **Is your mother in law aware that the girl is not bought food?** I told her everything, but like I told you (laughs) she said she would give me transport back to my village together with my children. What do you do for such a person? I have not gone back since she said that. We women suffer. I hear this one swearing that he will never wed again. He wedded a woman who left after 2 months. Now he is producing children and neglecting them. Now I have an idea about why women leave him. **Esther: Is Nakato taking her medicine well?** I force her. It is not bitter but a bit sweet. At first Nakato used to suck it but refused to take it with time. I now use force to get her to take it. I smash it, mix it with a little water on a spoon and force it down her throat.

**Esther: Do you see any difference since she started medicine?** There is a difference. Before she started she was thin and used to get frequent illness. **Esther: How are you managing without money?** You get ill and the illness heals by itself. **Esther: are you talking about malaria, how would it heal by itself?**  eee, when i fall sick you think he can find the time to take me for treatment? That would be a miracle. **Esther: can’t you go to clinics nearby?** They are there but they require money. Would I go there for free? I just stay home until the malaria heals by itself. **Esther: don’t you sometimes use herbs like mululuza?** I have heard about them but i don’t really know how to use those herbs. I just stay here and eventually the illness subsides. But on the days he leaves some good amount of money for food I squeeze it and get some medicine.

**Things considered necessary for the management of HIV/AIDS at home**

**Food**- The most important thing is food and may be sugar for Nakato. That medicine can make you dizzy without food.

**(Nakato started crying again. Her mother carried her and shook her to make her keep quiet. I observed that she could be hungry and asked when she ate last. Mutetsi told me she had last given her tea in the morning. I asked whether it was the water then she said yes. I asked if she puts any tealeaves in it, she said she doesn’t. She just mixes water and puts a little sugar. Water with tea leaves chokes her. She noted that the girl had eaten nothing since morning. She first gives her water and then she gives her medicine. She had to wait for the food she had started cooking minutes after I arrived. When her husband leaves enough money she buys her some bread for 500/= and gives her. She started preparing some ‘tea’ for the girl. We continued talking. She said when her husband is giving her money he doesn’t bother to find out their extent of need. He gives whatever he feels like giving that day. He sometimes leaves 200/=).**

**Esther: Was he staying here before you came to live with him?** We were renting in a different place. He had got me from my home in south western Uganda. He had come to see his maternal aunt who stays close to my parents’ home. She is the sibling who follows his mother. His aunt connected us and assured me her nephew was a good man.

**(The tea was ready. Nakato cried when she saw the cup. Her mother sat close to her and started cooling the water using two plastic cups. The sound of water pouring from one cup to the other filled the air. Nakato cried occasionally as she looked at the cups. I kept asking Mutetsi to buy the girl something to eat but she ignored me all the time. We continued talking about several issues as she poured the tea from one cup to another)**.

**Esther: do you have the nurse’s number?** Yes, but sometimes I have no airtime to call her. If the nurse doesn’t call, then we meet when we go there. The more times we go there, the more we see her. We last went there in December, then she bought her some milk.

**Esther: Have you been in touch with your family lately?** I have taken long without hearing from them. **Esther: do they call you now that you have one?** This phone is the one my husband had taken away from me and refused to give it back. When I started job searching I had to devise ways of getting it from him, because I gave people my number to alert or connect me to opportunities. When I took mine away he remained with none.

**Esther: Have you eaten anything since morning?** No, but I am cooking food. I have been fetching water since I woke up. I bought some soap today and want to wash. The baby has nothing to sleep in, all her clothes are dirty.

**(Silence ensued as she continued cooling the water. She tried to feed her but she cried. I told her to check if the tea wasn’t hot. She tasted and started cooling it again. We remained silent. I looked on as she poured the water from one cup to another)**

**(I broke the silence by asking whether he would let her work). Mutetsi:** He doesn’t give me the necessary support. My main intention is to leave this man for good. **Esther: maybe you never got to know him very well. Mutetsi:** we met in the village but I was working in a city suburb at that time. I had gone for Easter and to take my older daughter who lives with my mother support. He had also gone for Easter at his aunt’s place. His aunt had praised him as a good man. We connected and came back together. I didn’t even go back to the work place but went straight to his home. At first he was supportive, but changed when I delivered the twins. He used to give me enough money then **Esther: may be caring for the children had strained him. Mutetsi:** he does not have both of them so he can’t give that as an excuse. He is not injecting any resources in Babirye who lives with his mother.

**(The water was cool. She** **carried the baby and started feeding her. She sipped the water at a terrific speed and often choked. She didn’t let go of the cup until it was empty. I asked Mutetsi if she could get her milk now. She said there were diaries. I picked 10,000/= from my wallet and told her to think of getting milk for her now. She said she would buy later, Nakato, who was at the time playing with clothes, was now okay)**

**Esther: Did you disclose your status to any of your relatives or friends about her status?** I haven’t. The only person I have told is my mother. When I went to the village I told her. I am not really sure if she understood what being on ARVs meant.

**Esther: What challenges do you face in accessing services at the health centre?**

It depends. When you don’t go early, you spend the whole day there until your turn comes. But *musawo* buys food for Nakato. The moment she knows we are around, she organises something for her. We always pass by her unit before coming to the ART clinic. She is always there on Friday because it is a day for immunisation. She tells us to pass by her unit after getting medicine and when we delay to leave she buys food for Nakato and brings. **Esther: Does that mean you stay hungry?** I do. Sometimes I eat some of Nakato’s food. I leave as early as 6:00am when my plan is to walk. The good thing our days were harmonised so we come at once. At first the days were separate. Nakato would be due for refill this week, when mine is the following week. But I managed to lobby for our days to be harmonised. I told them that transport is a problem, then they changed. We mainly use taxi or boda boda. A taxi to the dispensary charges 500/= while a boda boda charges 1000/=. We are now given two months refill.

**Esther: why don’t you save some money the days he gives you a lot?** (laughs) Which much money? The maximum amount he gives you when he has helped you is 3000/=. **Esther: Does he provide other things like clothes?** He has no time for that. It is *musawo* who dresses Nakato. Their father last bought them clothes the day we baptised them. By surprise *musawo* tells you to pick clothes for Nakato to wear. She calls me. He has never given me any money to shop clothes for the children. Even when I was pregnant he gave me 10,000/= to shop for the baby (we both laughed). I got labour pains in the morning of Saturday. I told him I was in pain. When he realised I was badly off, he gave me 2000/= to go to a clinic for check up. I went to a clinic. I had never been told that I had two babies. She told me if I had bought few things, I should add more, as soon as possible because I would deliver by evening. I came back and told him they had told me I have two children, if we bought few things we should add more. He didn’t respond. He said nothing. That day he helped me and stayed around. There was a wedding in the neighbourhood. I stayed home. At 9:00pm I felt pain again. We went to the dispensary. He called a bodaboda rider who met us on the way and took us. I had only 2 sheets. The mid wife asked him for sheets. He got the two and gave her. I pushed the second baby. The mid wife came back and asked him to bring more sheets, he couldn’t find any. He didn’t even bother to look for more. The mid wife had to improvise by removing Babirye from one sheet and wrapping each of the babies in one. It is his paternal aunties who bought some clothes and also tore bed sheets into smaller pieces to create bed sheets for the babies. From what I see, he doesn’t care about the child that much. He has no time for that. I hear the mothers of his older children calling him to send school fees, but he doesn’t care. He hasn’t sent any of them school fees.

**Esther: how are you preventing yourself from conceiving again?** I am on an injection. I go to *musawo* she injects me every three months. *Musawo* advised me to start family. I opted for the injection. **Esther: Don’t you get any side effects from the injection?** I have not got any, apart from the disappearance of my periods. **Esther: have you discussed that with any health personnel?** No. **Esther: i think you should discuss it with *musawo,* it may bring you problems.** (She seemed to be surprised that I considered this a serious issue. Her facial expression indicated that she had taken it lightly).

**Esther: what food do you buy from the little money you are given? I** mainly buy posho, and then get Nakato a quarter kilo of rice. There is cheap rice that goes for 650/=. For sauce I know that the charcoal I buy cannot cook beans so I avoid them. I mainly buy silver fish and mix it with ntula (vegetables). **Esther: why don’t you use firewood?** It also requires money and there is no bush from where to pick firewood. **Esther: does Nakato like the silver fish? S**he does. *Musawo* bought her powdered silver fish that is packed, which I mix in her food. She also eats the unprocessed one. The only thing she refuses is posho.

**Esther: Which other resources do you considers necessary besides food?** Medicine (silence). **Esther: have you managed to keep all her appointments at the dispensary?** I missed once because I was sick. I was very sick with fever, I didn’t have transport, I didn’t have energy to walk. When the illness subsided I went and picked it. I was here buying some tablets from clinics. **Esther: but you could have gone to the dispensary for treatment.** **Mutetsi:** The issue is that at that time, I had no money and could only walk. I was too dizzy. I went there before I healed totally. I called mum. I called Nakato’s grandmother then she gave her maid 10,000/= and beans to bring for me. I called my mum in law and said i am dying of dizziness. She said it seemed I didn’t have food. I said I didn’t. There was a woman who had come this way to deliver. She was my friend and gave me her phone to call. I was there then her maid came and gave me 10,000/=. From the money she gave me I bought tablets, food and I felt better. When I got better I went and picked the medicine. That man left me here without giving me even 100/= to get medicine. You are sick and expect him to stay here and take care of you, it’s impossible.

**Esther: but did that man really love you or he was just looking for someone to keep his house?** (laughs) I can’t tell. But I no longer fall sick frequently like before I started medicine. At first the medicine affected me. I would get dizzy from the moment I swallowed it until the following day. But after a week I got better. It went by itself; I didn’t do anything about it. I think the body got used.

**(Nakato got hiccups. I noticed and told the mother to give her water. She got up, moved towards the utensils, picked a small white jerry can with a red cover, opened it and poured some little water in a cup. She came back and tried to feed the girl. She tasted a bit and pushed the cup away. I also tried to encourage her to drink the water. I spoke to her in Luganda, ‘drink the water’. Her mother always spoke to her in Luganda, although she had told me her husband was a Munyarwanda. Mutetsi became more forceful. She eventually drunk it then the hiccup stopped. I commented that perhaps it is the sugar that attracts her to drink the plain water she is fed on. Mutetsi concurred and said, ‘She doesn’t like plain water.’ I asked what she does when she has no sugar to add to the girl’s water. She said, ‘She starves until food is ready.’ I asked, ‘Even after swallowing medicine?’ She remained silent. Nakato started sucking her thumb. I commented that the sucking of her thumb could be helping her relieve the pangs of hunger. That could be the reason why she was managing to stay longer without food).**

**Mutetsi:** (narrates an incident with Babirye her other daughter) I brought Babirye here, because her grandmother had a trip somewhere, then she gave her to me. I was with her for 2 weeks. Babirye was not used to these conditions and cried throughout her stay. She would cry until the whole village gathered (we both laughed). There was no food but Nakato was quiet whilst Babirye wailed. Babirye is used to plenty. At her grandmother’s place, flasks of milk are always there. Their grandmother has cows. All the milk she gets from the cows is for Babirye. She doesn’t sell any of it. **Esther: what does your mother in-law do for a living?** She is a traditional birth attendant. She is well known at the dispensary. She picks medicine for several women. She also has houses for rent, about 8 cubicles. These also give her money. She sent me herbs when I was pregnant although I delivered from the dispensary. **Esther: why isn’t she giving you milk?** She told us to pick the milk, but the distance is big. It is about 5 kilometres and I would have to walk daily. That distance, aaa (expression for no).

**Esther: but her father should ideally have devised mechanisms of ensuring that the milk gets here. Does he even ever play with the child?** Love for the child. He shows you that he loves her. They told him that twins are problematic. His friend who gave him the food, he told him that I don’t wash his clothes, he has nothing to wear. His friend called me and told me that stuff. I told his friend, you man, instead of telling me those issues on phone, you should have come home, so that I explain to you your friend’s problems. Should I go to shops and steal soap, what should I do? I think his friend told him if he doesn’t show love to these children and mistreats them, twins are difficult, they will do you harm. He tries to support because of that fear. But he still doesn’t fulfil his responsibility to the letter. He doesn’t buy Nakato milk, I don’t understand. May be he thinks she is old.

**Esther: Does Nakato take porridge?** She does. **Esther: how about saving the milk by mixing it with porridge. Mutetsi:** you are right. In porridge one pours just a little milk. A litre of milk can take you for four days if prepared with porridge. If it wasn’t for *musawo*, I was feeling angry and wanted to abandon her with him. **Esther:** **she would have died long ago because none of them is aware that she is on ARVs or have you informed your mother in law?** She is not aware. I don’t understand how I got infected. My first born was okay. I tested several times when I was pregnant with her and further tested her. I didn’t sleep with any other man after separating with the father of the first born. The only man I have slept with since then is my current husband. I can’t tell him now before I get where to go. He may throw me out of his house. I wanted to get him tested through the health workers, but he was too smart for us. I had talked to the nurse who had been on night duty that day. When I explained my situation she agreed with me that he too had to be tested and started on medicine. *Musawo* asked me for his number. I gave it to her. She called and told him she needed to see him at the dispensary urgently. He came. It was in the evening. She told him, to discharge his people I want to first remove your blood and test it to prove what is really disturbing the child. If you refuse I won’t discharge them. I am going to retain them in the ward. He told her; if you retain them do so. I have gone, you won’t test my blood. I am okay. I am not sick. He went and left us at the health facility. We were discharged the following day. *Musawo* would come and see the medicine they have prescribed then buy it. He used to check on us in the evening and give us money for food for the following day. The first day he came, I told him to stay with the child then I went home and picked clothes, basins and other necessities. The day we were discharged, *musawo* called and informed him that we had been discharged. She gave us transport and gave me some money to buy her (touches the baby) milk.

**(She had been telling me about a woman who had called her when I interrupted her with a clarification. I took her back to the issue)** **Mutetsi:** A woman called and shouted at me. He had taken my phone together with my line. My line is Airtel whilst his is Mango (local telecom companies). I think he found it easier to give them my number, because Airtel is considered more user friendly by many. The woman called and abused me, asking me the capacity in which I was receiving her husband’s calls. That is the time I had got disturbed and wanted to leave. I asked him whether he knew the number. He abused me and said, in which capacity are you asking about this number, do you have anything to do with it? I told him a woman called and abused me. He jeered at me and kept quiet.

**Esther: Do you receive any visitors from home?** From my village? No. But I have a brother in Kampala. I often call this brother of mine, then he sends me 10,000/= when things are tight. This sustains us for a while. Even my mother sometimes I call her and ask for help. Even my mother in-law helps, but I rarely ask her. I mainly ask three people, *musawo*, my mother and that brother of mine in Kampala. I keep changing. If I ask musawo this time, the next time I can ask my mother or brother, like that. I don’t want them to feel burdened. **Did you inform any of your siblings that you were on ARVs)** No. My brother helps because he knows I am not doing so well. It is *musawo* who helps me with anything to do with HIV/AIDS. She advises and supports me. My husband is not interested. He will never know that the child is sick. He goes very early and comes back very late in the night. He has no time to look at the child. When morning comes and I feel the baby is hot, I take her to *musawo* who then shows her to the doctors. Since she got her cough treated, she hasn’t fallen sick.

**Esther: Do you cover her well, pneumonia comes with the cold?** Her grandmother bought them blankets. Also, since *musawo* learnt of the pneumonia, she bought her two overalls. They are the ones I dress her in overnight.

**(We were silent for a while. The room was quiet, except for Nakato’s bubbly sounds as she played with some clothes. I quickly thought of what to ask to break the silence. I asked if she had received any calls inviting her for jobs)**

**Mutetsi:** Someone told me they had a job at an island. It was a job related to cooking and serving food. I asked the person whether the baby would get a good place to sleep. The other person responded that it would be hard. I told them that the baby cannot stay in a cold place. The person promised to get me a better opportunity, but they are yet to call. **Esther: Who is helping you look for jobs?** My friends. I used to work with them in the city suburb. I called and informed them that I needed work, and asked them to help me look around for an opportunity that would allow me to work with my child. **Esther: do you have any friends in the neighbourhood?** (laughs) I only cooperate with one of my neighbours who is also a *nalongo* (a mother of twins). **Esther: Is your friend also on ARVs, have you disclosed to her?** I don’t know her status. She has also not disclosed anything to me. **Esther: have you made any friends at the HIV clinic?** My only friend there is *musawo*. **Esther: How about friends among PLHIV?** She kept silent for a while. The expression on her face indicated she was trying to think about my question. After a while she told me she had no friend among fellow PLHIV.

**Challenges**

**(She took a while to understand my question about the challenges she was facing in the process of managing HIV/AIDS. She asked me whether it was about swallowing medicine. I told her I wanted to know about the challenges she encounters in managing her illness in total.** She asked**, ‘**Getting medicine from the dispensary?**’ Esther: let’s start with that: Mutetsi:** May be transport. At the dispensary when you go early you don’t delay. But the people who work there are different. Some follow the order but sometimes you find those who start with the files for people who have just arrived and handle the people who came first last. But sometimes you find a responsible person. Sometimes we hurry, other times we delay.

**Esther: have you befriended any of the people who work at the ART clinic?** I don’t know the health workers there that much. It is only *musawo* who is my friend.

Another thing, they...tell me to pay money at the triage. **Esther: when did that system begin i haven’t been seeing patients leave any money there?** The system is there. **Esther:** **did you start paying from the first time you came?** Most of the time I don’t have the money. I have just been leaving the table without paying. But the last time I was there, last Friday one of the counsellors whose name I couldn’t remember, told her that they have been lenient with me for a while, but next time they need the 500/=at the triage. Be keen the next time you go there. You will notice that they ask them for money. It is only one counsellor who doesn’t ask for the money. I don’t know her name, I don’t know their names.

**Esther:** today I had heard people being asked, ‘Did you pay the 500/=?. The person said no, then they told them to bring it. **Mutetsi:** I thought it was an official charge by the health facility. People know it and they willingly pay the money. That 500/= charge is going to become another impediment, without it they will perhaps be sending us back home.

**Esther: any other challenges, what do you consider critical now that you know you have HIV and are on ARVs?** Getting food is problematic. When my husband leaves money we eat but when he doesn’t we survive on water. I now know that eating food before swallowing medicine is important, because the times I swallow medicine on an empty stomach I feel too dizzy. **Esther: Do you ever sit and share a meal as a family?** It is difficult, except on a few Sundays when he keeps home. He rarely stays at home on Sundays and when he leaves he never tells me where he is going. **Esther: perhaps he takes you for granted. He knows you have no money and so can’t easily leave. Mutetsi:** most likely, that is what he thinks. He knows I love my children very much, I can’t abandon them. That is what he is relying on.

**(Nakato started crying again. I told the mother that she could be hungry again and suggested that she checks on the food, it could be ready. She got up and went to check on the food. She came back after a few seconds and told me it wasn’t ready)** As she sat she said,

‘At home we are needy.’ **Esther: in the village?** ‘eeeh’ (an expression for yes). The socio-economic situation at my maiden home is not impressing. Both our parents are alive but we are many children. We are 12 children. Only the elder ones went to school. The girl stopped in S6 whilst the boy stopped in S.4. But the rest of us didn’t study that much.

**Esther: Is the boy the brother you said always helps with money?** He is not the one. The one who helps me is the one I follow. He stopped in P.5 and works around Kampala. He provides casual labour at building sites. The older one is in the village. He is a farmer. The girl who stopped in S.6 got married and lives in Sembabule district. She is currently not working.

**Esther: why don’t you ask for any support from these two especially the farmer? Farmers usually have money. Mutetsi:** yes he has money. As you know what happens amongst siblings. Some may be easy whilst others are difficult. It is my brother in Kampala that I find easy to ask, and when he has money and I ask him, he gives me. But the other one would ask me several questions. What are you going to use it for? How about your husband? Many questions and may eventually not give me.

**Esther: does your family know where you stay?** They don’t know this particular place but have been at my mother in laws place during the baptism of the twins. I produced my first born at 17 years. She had got married with her father, but decided to pull out because his clan was ‘dead’. They had no manners and were not developmental. I left him while I was pregnant and so had delivered the girl at home. I am tired of marriage. If I get a job and life gets better, I don’t think I will get in to marriage again.

**Esther: is your first born in school?** I am not sure whether she is in school. When I call her father to give me fees, he says he wants to stay with her. Her father doesn’t give her any support; she’s totally in the hands of my parents. I just took her back to the village. When I got this man, I went back and picked her. But I was compelled to take her back because this man didn’t like her. He was mistreating her. **Esther: how?** You can see a person who doesn’t like your child. That girl would fall sick but he would never give even 500/= to buy her medicine. He would instead accuse me of clinging on to the child when her father wanted to take her. There are some people I consulted. They cautioned me not to give my daughter to her father, saying, a girl child being given to a man living alone without a wife. Men of these days are crazy. I looked into it and decided not to give him the girl. When we went to the village with him, I took her along and left her with my parents. They try to take care of her, but I need to look for her school fees.

**Esther: how do you plan to leave this man?** I will just wake up one day and leave. **Esther:** but don’t spoil your relationship with the family, they are the ones who may remain with your children. You need to do it tactfully.

**(Nakato started dosing. She got up to take her to the bed behind the curtains. I decided to leave and told her I had to go so that she could embark on the washing of clothes. She laughed but I could sense she had welcomed the idea. She put Nakato to bed and came back to escort me to the road. I told her I had another home to visit but needed to eat first. She said there was no decent eating place here, but I would find several when I reached the main road. I got out, put on my shoes and waited for her to come out. She got a pair of sandals closed the door and wore them. We started walking towards the road. We walked side by side along the narrow dusty road. She greeted several people as we moved.**
