# Supplementary material for: Social capital and resilience among people living on antiretroviral therapy in resource-poor Uganda
Source: PLoS One. 2018 Jun 11;13(6):e0197979. doi: 10.1371/journal.pone.0197979 (PMC5995438; doi:10.1371/journal.pone.0197979)
Supplement: S8 File — (DOCX) [file pone.0197979.s009.docx]

**Client ID: 011**

**Name: Rose (Pseudonym)**

Status: **Second line**

**Section 1: Socio demographic characteristics**

Age:  **35 years**

Sex:  **Female**

Marital status: **Separated**

Highest education level attained: P.4

Main Source of livelihood: **She is ill and depends on her mother for survival.**

Ethnicity: **Muganda**

**I: When did you enroll on treatment?**

R1: I am not very sure of the year but at that time we used to come there to sing and dance.

**I: Singing?**

R1: Yes

**I: Now for you how did you manage to reach there?**

R1: We use these usual transport means since it’s a short distance from here. It’s UGX3500 to and fro when using a taxi.

**I: Meaning it might even cost you UGX5000 to and fro almost?**

R1: Yes

**I: How did you use to manage your life during your marriage?**

R1: During that time I used to sell bananas so that I can be able to support this child for school.

**I: Is this child for that particular man that you used to stay with?**

R1: Yes

**I: You used to sell bananas where you were living?**

R1: Yes. That was the business that I used to run in order to support this child who takes care of me, for school.

**I: Did you take this child for HIV+/AIDs testing?**

R1: Yes

**I: Was she positive?**

R1: No. She was HIV negative

**I: Okay. That was great**

R1: I tested all my children for HIV+/AIDs and they were all negative.

**I: What about your new husband?**

**Where is he anyway?**

R1: There at his home place. But it was me who took him to the hospital for HIV+/AIDs testing.

**I: So you’ve never visited him since the time you left?**

R1: No

**I: By the time you joined that group at the treatment centre, did it have that name “the support group” already?**

R1: Yes

**I: How often were you supposed to meet?**

R1: We used to meet every time you would go to get the medication.

**I: Did you use to pick your medication every month?**

R1: At first I was provided with the medication which could take me for one month. Then the next time i was provided with what could take me for two months. So in total there were three months.

**I: You used to get Septrin only for those three months?**

R1: Yes

**I: Did they use to examine your CD4 levels during that time?**

R1: Yes they used to

**I: How were your CD4 levels by the time you went there?**

R1: Honestly I don’t remember

**I: You don’t remember?**

R1: Yes

**I: When did they change your treatment?**

**When were you stopped from using Septrin?**

R1: After three years

**I: That means it was around 2008 right?**

R1: Yes

**I: Your CD4 levels were examined every after how many month?**

R1: After six months

**I: Okay. So what did they tell you after examining your CD4?**

R1: I was told that my CD4 is 9. So I started on a different treatment and it’s the one I am still on at the moment.

**I: Meaning you’ve been using the same drugs for the past years right?**

R1: Yes. They have just changed my treatment of recent.

**I: Why have you eaten little food?**

R1: It’s okay. But for me I don’t like posho

**I: Meaning you prefer sweet things**

R1: Yes. I don’t like eating matooke because whenever I eat matooke I develop wounds inside the month. So this is the type of food that I use most.

**I: Okay. According to your information it seems that you’ve been at here since 2005. Don’t you think you are going to make 10 years now!**

R1: No. It’s now one and half years since I left my marriage. (Since I left my home and husband)

**I: Remember earlier you told me that in 2005 it’s when you started developing the wounds and this forced you to leave your home and come here.**

R1: Yes I came here but after receiving the treatment again I went back to my husband in Kampala.

**I: Okay**

**Did you inform him about your HIV+ results after testing?**

R1: Yes I did. But at first I was afraid of going back home to meet him thinking that he might accuse me of infecting him with the virus.

**I: How sure were you that it’s you who transmitted the virus?**

R1: I wasn’t sure of who might have caused the virus in our family and I don’t put myself in that position either. But as you understand, men are so unpredictable and very difficult to understand my dear. I was so scared

**I: You never know it could not be you who might have caused all this…**

R1: After a few days he came for me because I had decided to stay at my parents’ home since I was scared. But my mother told me to leave with him since I had already explained to him about how my results were after HIV/AIDs testing.

**I: Did you inform him about your results?**

R1: Yes I did inform him. Then after like one year and half, my husband too developed herpes zoster.

**I: For him had not tested for HIV yet?**

R1: No. I always advised him to go for HIV/AIDs testing but he refused. He never wanted to go for testing.

**I: But for your case you used to go for your medication right?**

R1: Yes I used to

**I: Okay. By that time, were you still running your business of selling bananas?**

R1: Yes

**I: Nowadays herpes zoster is too much. Even when I visited the treatment centre I came across about four people with the same case. And they say it is so painful. Is this true?**

R1: Yes it is. It is too painful. So I took him to “Namirembe hospital” for HIV/AIDs testing.

**I: Was he tested**?

R1: Yes he was tested and found HIV+. Since that day it’s from “Namirembe hospital” he receives his medication.

**I: When they found out that he is HIV+, was he put on treatment immediately?**

R1: Yes he started the medication immediately they found out that he was HIV+

He was told to go back for the medical checkup after one week and he went beck immediately after one week.

**I: Did he start with Septrin too**

R1: Yes. He started with Septrin at first.

**I: Okay. When did you leave your husbands’ home?**

R1: Just a few months back.

**I: That means it isn’t that long since you left your home.**

**True?**

R1: Yes

Only that he was never providing me with anything and that’s the reason to why I was forced to leave.

**I: Maybe he doesn’t work. Does he have a job anyway?**

R1: It doesn’t matter. You mean he doesn’t know that it’s his responsibility to take care of someone who is sick in his home and any sick person has to be provided for?

**I: What is the type of his job?**

R1: He owns a shop

**I: Dealing in which products?**

R1: He deals in traditional, cultural and local herbals.

**I: Like which cultural products?**

R1: calabashes

**I: Okay. Maybe his shop isn’t that productive and he might be not earning that much. Don’t you think so?**

R1: I don’t know.

**I: Does he still report at the hospital for medication or not?**

R1: Perhaps he stopped from reporting at the hospital for the medication.

**I: Do you also fail to report for medication sometimes?**

R1: No. When I fail to go there by myself I make sure that I send my mother to get the drugs for me. I have never missed.

**I: How did it start for you to decide and leave your home?**

R1: Since he was not providing any more, I got more stressed. One day I woke up early in the morning and packed some of my belongings then left his home together with my children.

**I: Maybe he expected to support yourself since you were also working.**

**Don’t you think so?**

R1: Perhaps. The situation wasn’t that easy during that time when I was leaving to an extent that he never wanted to share any of our belongings with me at all.

**I: Things like what?**

R1: Things like cows, goats, hens among others yet they were all mine.

**I: Okay. Did you engage the local leaders for example: the L.Cs in your issues?**

R1: Yes. And my husband asked to be compensated with money

**I: For what reason?**

R1: That he also wasted a lot of his time taking care of cattle. As a result we ended up compensating him with one goat and he accepted.

**I: Did you agree on your issues as partners or you just woke up one day and left without informing him at all?**

R1: I just decided to leave. It was my personal decision.

**I: Then that could be the reason to why he decided to detain all of your belongings.**

R1: Perhaps. But he couldn’t handle my problems any longer.

**I: Okay. Earlier you told me that the type of medication you are using now is reacting well on you.**

Is that true?

R1: Yes it is

My health condition has improved now.

**I: Okay**

**When did the medication you were using before stop working on you?**

R1: If I remember, I have suffered for almost a full year. But I always submitted in my complaints to them. Though they always kept on promising to change and put me on a different form of medication.

**I: How did you use to feel during that time?**

R1: I was too weak and I could fall sick almost every day.

**I: What did you use to suffer from most?**

R1: Fever and headache

**I: You’ve been suffering from malaria and headache most?**

R1: Yes

**I: What about cough?**

R1: For the case of cough I have been experiencing for two months now.

**I: Were you tested for TB?**

R1: Yes I was tested for TB of recently because the cough was persistent. I didn’t understand why?

**I: Maybe your immunity level is getting low. You never know it could be one of the reasons why it’s happening now. Did they examine your immune levels for the next time?**

R1: Yes they did but I am not aware about the results yet.

**I: Okay. What reason did they give to change your type of medication?**

R1: They realized that the drugs which i was using were not working anymore. Maybe the virus is now stronger than the type of medication that I was using before.

**I: Okay**

**Your health condition was worsening instead. I can see it by myself you’ve lost weight.**

R1: Yes

**I: Had you gained weight before?**

R1: Yes

**I: You’ve lost weight in what period of time?**

R1: Just in two months.

**I: Seriously it’s like you’ve been sick for five years.**

**I: What medicine are you taking?**

**R1**: I don’t know their name, but they changed me to the second line.

**I: I will find out the type of medicine or drugs that you’ve been using. But I think you’ve been using CBV/N.**

So you’ve been taking your medicine only in the night right?

R1: Yes. Only in the night

**I: Were you not given any medicine to be taken during day time?**

R1: No please

**I: The kind of medicine that you were given the doctor ordered you to take it both day and night?**

R1: Yes please

**I: Then that means you’re on CBN/N if I am not mistaken.**

R1: Perhaps!

**I: But for the first time it used to work for you and your health condition had improved right?**

R1: Yes please. Even my wounds were healed during those days

**I: Okay**

**For now what we are interested in are the things you consider important to manage treatment in the home. What are some of those things?**

R1: Money for transport

**I: Money for transport has to be provided?**

R1: Yes

**I: Okay**

R1: I love eating and in time.

**I: You love eating food?**

R1: Yes

I would love to be provided with everything that I need in time.

**I: With Everything?**

R1: Yes

**I: Do you sometimes crave for certain foods?**

R1: (Laughter…)

**I: (Laughter…) Like a pregnant person? There was a certain man who told me that when you on ARVs) you crave for certain foods. What is your opinion?**

R1: That is true. For me when I happen to feel like I need to eat some meat, there and then I inform my mum then she orders for some from the butcher and we prepare it.

**I: Okay. Earlier you said that you would love to be provided with money for transport and food!**

R1: Yes

**I: What else do you consider important?**

R1: (Laughter…) please suggest some for me

**I: Take your time and think about those that you find necessary as far as your health condition is concerned my dear**

R1: We don’t have enough medicine and drinks.

**I: Even the drinks too?**

ALL: Yes

**I: Most especially what?**

**Water or?**

R1:Yes. Water and medicine. Water is so important and in most cases its water that we need most.

**I: Okay. What about the medicine! What kind of medicine would you need to be provided for?**

R1: That’s what I told you about earlier that it’s better to be provided with transport so that I can be able to reach these places from where I receive these medicines and in time. Then I won’t run out of my medicine.

**I: I remember you told me that you have to visit these health centers after two months**

R1: Yes

**I: Then that means they have to carry out a medical check up on you in order to examine how the medicine is reacting. But you said that at the moment you are not experiencing any issues since you started this particular HIV/AIDS form of treatment right?**

R1: No please

**I: Okay**

**What about the other drugs that you used to use at the beginning?**

**How did it react on you?**

R1: During that time there was no change at all. Instead my condition was worsening. I used to develop skin rashes but not these big ones in size. I use to develop the small ones.

**I: Was that last year?**

R1: Yes please. But during those days, we used to apply some cream on our skins.

**I: Who used to provide you with those creams?**

R1: One of the health workers used to sell them.

**I: Maliza?**

R1: Yes

**I: Nakacwa?**

**Because there is a certain lady who sells those creams up to now**

R1: The other name was Maliza but not Nakacwa.

**I: The one who normally operates on “Tuesday”?**

R1: Yes

**I: That’s madam “Nakacwa”**

**Her name is madam “Nakacwa Maliza”**

R1: Okay. That cream used to work well.

**I: She is still sells them. Have you ever used them, really?**

R1: Yes. But I stopped a long time ago when my skin improved.

**I: So you stopped using the cream when your skin improved?**

R1: Yes

**I: But it used to work for you right?**

R1: Yes

**I: Okay. At how much did she use to sell them?**

R1: UGX 1500

**I: Okay. But you didn’t say anything regarding the person who is taking care of you.**

**Who cooks for you?**

R1: The wife to my brother.

**I: Okay.** Even I see you delegating these young children to do some work for you. That means they do help you right?

R1: Yes

**I: Even your mother too. Almost does each and everything here**

R1: Yes

**I: For me I think these people who take care of you are so important, not so?**

R1: Yes they are so important to me

**I: So! As a home where do you get the food from?**

R1: We have a garden as you slop down there.

**I: You have a garden?**

R1: Yes

**I: Even the potatoes you get them from that garden**

R1: Yes please

**I: Okay**

If you are to estimate, among these things which one is more important by giving them numbers?

I.e. The most important takes the 1^st^ position, the next one takes the 2^nd^ position and then others follow.

Which one do you think should take the first position?

R1: (1) Provision of money for transport

(2) Access to Medical facilities (to be provided with enough treatment)

(3) Feeding (availability of everything that I need to eat and drink and in time)

**I: Okay**

**What do you say about the issue of “Hospitals”? What are some of the medical facilities that you would need to be provided for in these hospitals in order for them to deliver a better health service?**

R1: The machines to be used for blood testing are very important.

**I: Okay**

R1: Even the health personnel who give out medication.

**I: Meaning availability of “medicine” is very important**

R1: Yes

**I: Has it ever happened to you that you visited the health center and you didn’t receive treatment? Then you were told to check after one month?**

R1: Yes. It has ever happened to me

**I: So you’re among those people who were affected and experienced that issue which happened around September of this year?**

R1: Yes

**I: And also you were told to wait for a full month?**

R1: Yes. But for my case I was given some few tablets.

**I: They gave you some few tablets and then you were told to comeback after a certain period of time right?**

R1: Yes. But the period which they told us wasn’t that long. We were told to comeback after three weeks and we went back after that period we were given.

**I: Okay. So you even use Septrin?**

R1: Yes I do

**I: Why do you use Septrin yet you’ve been using ARVs too?**

R1: I use Septrin with a purpose of healing these other infections like cough and to improve on my immunity generally.

**I: Okay. So according to you it has worked very well?**

R1: Yes it has worked for me very well

**I: Okay. What type of medication do you use to treat your cough?**

R1: They always provide me with Septrin

**I: Meaning you don’t use any other medication apart from Septrin?**

R1: Yes

**I: They didn’t provide you with any other type of medicine?**

R1: No

**I: Normally they used to give out capsules. Have you ever been provided with capsules for cough?**

R1: No

**I: Okay. What about malaria?**

R1: No. Nowadays I rarely experience malaria.

**I: Okay. What do you normally do when you get malaria?**

R1: I visit the health facility.

**I: Okay. Now let us look at the community where you live. What is necessary for you to manage HIV?**

R1: It would be better if they facilitate our health facility with enough drugs among other medical facilities.

**I: Drugs?**

R1: Yes

**I: Assuming they happen to provide such services here at the local facility, would you ask for a transfer?**

R1: Yes I will ask for a transfer to our health facility since it’s near here.

**I: Okay**

What else would you need to be provided or put in place here in your community in favor of PLHIV just like you?

R1: (Silence)

**I: How about friends? Do you have any friends who come to visit you?**

R1: Having friends it’s very important. I have them and they always come to visit.

**I: Okay**

**Your friends those you met in town?**

R1: Yes. Even those who live within this community also come around to visit me.

**I: How did they come to know that you are sick?**

R1: As usual information is shared among friends. So when everyone concerned receives such information comes around. They always come around to visit me.

**I: Okay**

People always talk about this issue of “stigma”. Do you have any problem regarding such issues?

R1: No

**I: What I realized is that: as I was travelling to mukono, you find that most of PLHIV travel far for treatment. Those from Mukono go to Naggalama while those near Naggalama come to Mukono. When you ask them why they like going far they respond that they don’t like visiting those close facilities because people living in those areas know them already.**

R1: Yes that’s true

**I: They also added on that when you happen to go there, as you look around you see people talking about you. They even point at you using their fingers when you are seeing. So that’s the reason why they hide now days.**

R1: Yes that’s true. Also there is a day when I had gone there to receive my treatment. Reaching there I fell asleep but by the time I woke up, everyone was looking at me and talking too much. That made me so uncomfortable.

**I: But I think such people might have come for the drugs too. Not so?**

R1: Yes. Such people are also another problem.

**I: What about the community?**

R1: I have not experienced any problem with the community yet.

Actually it’s like the majority here are HIV+ (Laughter….)

**I: How did you come to know about that?**

R1: No one is safe here. Even you hear people talking about them.

ALL: (Laughter….)

R1: Actually there are those who were infected before me.

**I: Meaning they are receiving the same HIV+/AIDs treatment but in a private way right?**

R1: Yes. They are all on medication only that for me I exposed myself and for them they didn’t. That’s the only difference. So in the community, no one has ever stigmatised me at all.

**I: Even you still have all of your friends though you are weak now. Actually even you no longer work?**

R1: Yes. I still have my friends

**I: Do they come and visit you?**

R1: Yes. They come and visit me

**I: When you go there for treatment or medication do you always happen to meet some people that you already know?**

R1: There at the treatment centre?

**I: Yes**

At the treatment centre

R1: No. What I know, most of them travel far”. They don’t visit the nearer treatment centre where we normally go.

**I: Others are in Kampala among other areas. Actually there are some that I know who always come from “Juba” (sudan) to receive the medication from treatment centres in Mukono**

R1: Yes. That habit is too much and they are many of them

**I: They are many**

R1: Yes. But people are still afraid. They lack self esteem too. But for me though I have been developing skin rashes of late, I never thought of putting on long sleeved dresses or long skirts. Never.

**I: Now for you how did you develop the courage and the confidence of not putting on long sleeved dresses or skirts given the fact that you had started developing those skin rashes?**

R1: I have to encourage myself because these rashes were no longer healing. Every time when I used to put on such shirts my body temperatures would rise immediately. That used to make me so uncomfortable and I had to get used on short sleeved dresses and shirts. If you realize that someone is persistently looking at you just because you have those unusual skin rashes, turn your eyes in a different direction. Just stop looking at him or her and move on as simple as that. But it’s good now my skin is healing and getting better.

**I: Okay. So there are no health facilities or clinics that you visit within this community because when I look on this medical form, they prescribed you medicine some of which was not available?**

R1: No. Apart from a certain woman who they normally call mu seventh day. That’s the name they normally call her.

**I: Okay. So as your coming you don’t stop in town to buy medicines instead you visit that so called “mu seventh day”**

R1: But to simplify it more, I might decide to visit that pharmacy in town first than any other clinic because at times you reach there when they don’t have the drugs. But still if I happen not to find those drugs there when I visit those clinics, I visit madam “mu seventh day’s” place.

**I: Okay. What about when you happen to face a sudden infections or sickness, where do you normally go?**

R1: I go to that women’s place (mu seventh day’s clinic)

**I: Meaning it’s the only place which is near you than other places.**

R1: Yes

**I: While here, have you ever got malaria and you had to travel late in the night?**

R1: No please. I have never experienced it

**I: Okay**

Do you have some drugs or medication that you keep at home?

R1: Yes please

**I: That of “first aid”?**

R1: Yes. Mother buys and we keep it here at home

**I: Okay**

**Like which one?**

R1: She buys “Panadol, medicine for malaria and that of flu.

**I: All those types of medicine or drugs are ever available?**

R1: Yes

**I: They have to be in place purposely for emergency cases?**

R1: Yes, for instance: assuming one of the daughters falls sick most especially during night, they use some of those medicines as first aid.

**I: Okay, I was told that she has many orphans. Is that true?**

R1: Yes she does have many grandchildren

**I: Is that medicine placed there only for you because you have HIV?**

R1: No. It’s not for me alone. It for everyone who falls sick in this house

**I: Meaning it’s only your mother who is taking care of you?**

R1: Yes

**I: How about these other people most especially the relatives?**

R1: When people discover that you’re HIV+/AIDs, they give up on you, neglecting you and they don’t even remember you.

**I: Okay. What about the community?**

R1: The people within the community are good people. They don’t isolate me and they always visit. Sometimes some of them make juice and come with it for me. Actually we receive many visitors every day

**I: Sure?**

R1: Yes

**I: They always come to visit you but when they are not even your relatives?**

R1: Yes. Most of them are not my relatives

**I: (Laughter….) how and when do your relatives help you?**

R1: Family issues are not that easy to understand. From nowhere someone just behaves so strange or treats you like you had a fight with him or her.

**I: Even your mother never bothers to inform them about her daughter’s sickness?**

R1: No she doesn’t. My mother is too kind and she tends never to bother others when having issues. At times she calls them on phone but still they end up doing nothing at all.

**I: Still they don’t come to visit you?**

R1: Yes

**I: So she decided to suffer alone with you.**

All: Yes

**I: Okay**

**Then you should thank GOD that your mother is still alive**. R1: Yes. She has really taken good care of us.

**(Her brother who is also HIV positive and at home to recuperate joins the discussion)**

**I: Where did you use to stay before you came here?**

R2: “Makindye”

**I: You used to work from “Makindye”?**

R2: Yes

**I: What was the type of your job?**

R2: I was working as a painter.

**I: Is it finding out that you were HIV+ that forced you to stop from working or what?**

R2: No. It is because I had developed a serious cough.

**I: Do you think it was those paint chemicals which caused cough infections?**

R2: I really don’t know.

**I: Cough was so serious?**

R2: Yes

**I: A critical cough, sweating throughout and losing weight are signs of TB. Did you experience any of those during that time?**

R2: No. I only used to cough. I had a strong cough

**I: Was it Mother that you called to discuss about your issues?**

R2: Yes

**I: Were you married by then?**

R2: No

**I: You were not married yet?**

R2: Yes

**I: Okay**

How long have you spent here in this home?

R2: Around 7 months

**I: So when you developed that cough immediately you called your mother on phone?**

R2: Yes

R1: For her she told him to come back home.

**I: And you came back?**

R1: Yes.

**I: Its mother who makes all the decisions?**

R1: Yes. Immediately when he reached here, mother took him to the health facility

**I: She took him to the health facility?**

ALL: Yes.

**I: What did you use to treat yourself during that time?**

R2: Every day I used to wake up very early in the morning to go and work and we used to move in many different places. But mostly we used to stage along “Shell Bugolobi” at the black gate. In that place there is a company known as “living goods” which had just established a few years back. It is where I used to work from.

But we used to work a lot. Actually by 5am I had to be at work already. I used to wake up every early in the morning.

**I: So are you planning to go back for work?**

R2: Yes

**I: How did you sign?**

Did you ask for a leave or?

R2: Yes I did. My job is still available

**I: Your job is still available?**

R2: Yes

**I: Then that’s good because I thought you lost your job.**

**So when you were given that leave mother told you to come?**

R2: Yes

**I: What did you use to treat your cough?**

R2: For me I didn’t know anything. I thought it was a normal cough.

**I: You used not to take any medication at all?**

R2: No. I used not to take any form of medicine at all because I didn’t know anything about what was happening with me during that time. I also used to develop some malaria and I think that was around 2009. But for that case I used to take some medication for malaria.

**I: What type of medication?**

These coartem?

R2: Yes

**I: You used to take a full dose or?**

R2: I used to swallow like four tablets a day and you could get better. But the problem was that after taking some of the medication and you feel that you’re fine; you stop using it and don’t complete the dosage. So after a short period again you fall sick.

**I: So you had malaria and at the same time TB?**

R2: Yes. I used to cough persistently by the time I came here

**I: By the time you came here had you developed malaria already?**

R2: Yes. I wasn’t working but I used to cough and was sweating all the time my dear.

**I: That is what we call “TB”. It can make you sweat throughout. You start coughing, you develop malaria and you become weak. Those are some of the TB signs**

R2: Yes. I used to sweat throughout.

R2: He used to sweat too much.

**I: Okay**

R1: He used to sweat to an extent that even all the bed sheets and the mattress on the bed could get totally wet.

**I: Just in one night?**

R2: Yes

R1: Even when I happen to take any simple medication like panadol it’s enough to make me sweat. When I visited the hospital, I was put on drip immediately.

**I: Maybe you had lost a lot of water since you used to sweat throughout.**

R1: But I used to drink a lot of water. The first thing I used to do when I woke up in the morning was to drink water. I used to do it every day.

**I: Sorry. So when mother took you to the hospital you were admitted and put on drip?**

R2: Yes. By the time she took me to hospital I had already visited that dispensary.

**I: You had visited that dispensary first?**

R2: Yes

R1: Yes. But no one was taking care of him all the times he went there.

**I: Which clinic?**

**The one which handles HIV+ cases?**

R1: Yes. That’s where they tested for TB from. He first visited a certain clinic then the Doctor told him to visit that one which handles HIV+ cases to be tested for TB first. Normally after getting your samples they tell you to come back for your results at 2pm.

**I: Did he first visit the other dispensary which handles HIV+/AIDs cases?**

R1: Yes…So because of that much time he spent there while waiting for his results he became unconscious. He didn’t even know how we brought him back home. Actually we never went back for his TB test results.

**I: Are sure you never went back for his TB testing results?**

R1: Yes

**I: He waited for too long right?**

R1: Yes

**I: Who did you go with? Mother?**

R1: No. He went with my elder brother’s son the one I told you about. They used a bicycle

**I: A son to your brother the one you told me about earlier?**

R1: Yes. He is also a good person. He takes maximum care of him.

**I: Okay. Was it your mother who asked your brother to send his son or?**

R1: No. They also stay with us here

**I: They stay here with you too?**

ALL: Yes

**I: Okay. So he is the one who escorted him to the dispensary and brought him back when his condition had critically worsened?**

R1: Yes

**I: How did they reach here?**

R1: They used a motorcycle.

**I: They used a motorcycle all the way from Town?**

R1: Yes. There is a time when he fainted and we took him to hospital

**I: Did you use a car?**

R2: Yes

**I: So that’s where he was admitted from?**

R2: Yes. He was admitted immediately. They attended to him immediately only that money also became another issue. But in all he was treated because we were not sure that he would survive.

**I: Are you on ARVs treatment at the moment?**

R2: Yes. I am on ARVs

**I: Is it packed in bottles or?**

R2: Some are in bottles and then others in packets.

**I: Meaning you take both of them!**

R2: Yes please

**I: Did you start ARVs treatment right from the time you were admitted?**

R2: Yes

**I: Did they counsel the both of you?**

R2: Yes

**I: You and the other person who takes care of you?**

R2: Yes

**I: Okay. When did you start receiving HIV treatment?**

R2: Last year

**I: Meaning it’s coming to 8 month since you started receiving HIV treatment**

R2: Yes

**I: So far how many times have you visited the treatment centre?**

R2: Two times but I will have to report there again on Thursday next week.

**I: Assuming today it’s the day to visit the treatment centre, will the bodaboda rider pick you from here or?**

R1: Yes. I call him on phone and then he comes

**I: Do you have their phone numbers?**

ALL: Yes

**I: For all those bodaboda riders at the stage or just a few of them?**

R2: Only for those who are reliable

**I: So you arrange your program the day before in the evening?**

R2: Yes. Even when you call him early like at 6am, still he will come.

**I: Meaning at least you should be having around three phone numbers to be on the safe side right?**

ALL: Yes

R1: Almost everyone here has the numbers for those bodaboda riders. For any emergency you just give them a call and they will come immediately.

**I: Even when it’s late in the night?**

R1: Yes they will come when you call them

**I: Okay**

R1: They are so helpful

**I: Okay**

R1: What I have liked about you is that you’re very easy to be directed…

**I: Thank you. But I was writing down all the direction. I was moving with my book here with me where I wrote all the directions. So all the time I was following what I wrote in this book just as you directed me. Remember you told me that I will come across a railway line.** R1: By the way thank you so much for coming to check on us. It is so encouraging.

Actually I have been waiting for you heartedly.

**I: Thank you**

**I will spare sometime and check you once in a while**

R1: Thank you

**I: For him he is soon going back for work right?**

R2: I am still here for some few months.

**I: How long is your leave?**

R2: Four months

**I: Four month?**

R2: Yes

But after sometime I requested them to extend the leave since I was still in a bad health condition.

R1: They also come and check on him. Many people from Kampala come to check on him.

**I: Okay**

His friends or?

R1: Yes

And they come with many things for us.

**I: Those friends who come to visit you do them know that you’re HIV+?**

Did you inform them?

R2: Yes. But some of them are not yet aware. Actually no one would know that I am HIV+ even me personally. But I used to cough a lot and it was a heavy cough.

**I: Okay**

Have you ever used any local herbs?

ALL: Never

**I: Is your mother aware of those local herbs?**

R2: No. We’ve never come across them around this place even

**I: You only use tablets?**

ALL: Yes

**I: What about the one she uses when working on those women giving birth and how does she manage to do that without any machines which are used in other hospitals?**

R1: For any normal woman I don’t think it’s hard to push when giving birth. Am very sure they only apply those machines when she has failed to push the baby.

**I: Okay**

During your pregnancy of this baby, did you undergo the PMTCT treatment?

R1: Yes I did

**I: Were you given some drugs to take during your pregnancy?**

R1: Yes they did and I was using them

**I: Okay**

Meaning after giving birth to your baby was also put on the same type of treatment right?

R1: Yes

**I: Did you breast feed your baby for six months just as any other mother is supposed to do?**

R1: No please. I was given drugs and advised not to breast feed. I never breast fed my baby until she was tested for HIV.

**I: Okay. I was told that once you start ARVs treatment you’re not supposed to use any other form of medication. But how about when you are pregnant?**

R1: Assuming I am pregnant!

**I: Yes**

R1: We use them just like any other pregnant woman. Do you think I can fail to use any local herbs for example *emumbwa*

**I: But why? Because I was told that a person on ARVs treatment is not supposed to use any other form of medication.**

**Why?**

R2: It depends. For me I think the reason why a person is advised not to use any other medication when on ARVs treatment is because it’s very dangerous to take ARVs and any other medication at the same time.

But if a person takes ARVs in the morning let’s say at around 8am and then take any other form of medication in the evening, there won’t be any problem.

**I: Meaning that it can only be dangerous when you take ARVs and any other form of medication at the same time right?**

R2: Yes please

**I: They also allow you to use other drugs but after some time when you’ve taken ARVs**

ALL: Yes

R1: Like after one hour.

**I: There a certain herbal medicine called *ebombo*. Do you also apply it while bathing sometimes?**

ALL: Yes we do

R1: You wake up in the morning, prepare some herbals for example: *ebombo* or *omuluuluza* and mix it with water that you will have to use for bathing because they also improve on your health condition. You mix it with cold water.

**I: Okay. Do you feel different immediately after using it?**

R1: Yes. You feel so good after bathing with that water mixed with *ebombo* and *omuluuluza*

**I: Where do you get those herbals from?**

R1: From there in the bush

**I: Okay**

How did you come to know about these herbals?

Did you grow seeing your mother using them?

R1: Yes

**I: Okay.** For me I am not sure whether I even know how **bombo** looks like

R1: (Laughter….)

**I: Well!**

Thanks for your conversation. I promise to come back and check on you.

Hope Ernest will still be around by the time I come back to check on you. Hope he wouldn’t have gone back to Kampala for work!

R1: No

He will be around

R2: I will be around by that time

**I: When do you expect to go back?**

R2: I have to make sure I am within Kampala by the time “*Amasiro*” is launched.

**I: When is the launching?**

R2: This month

**I: So you will have to attend the “Amasiro” launch first, come back here and then after a short period you go back to Kampala for work?**

R2: Yes

**I: Health wise do you feel good at the moment?**

R2: Yes I do

**I: Okay**

**Then you should really thank GOD for having a great mother like her because for other people wouldn’t even care.**

ALL: Yes we do

**I: Now for your case you don’t work anymore but they provide you with each and everything that you need.**

R1: Yes they do. My mother is caring. When you ask her she provides. Like, when I need money for transport to come there I inform her in advance. She always gives me.

**I: Then you should really thank GOD.**

**Ernest is your company still paying you the salary?**

R2: They send me some little money through mobile money.

**I: Why can’t they send it on your account?**

R2: I never gave the company my Account number

**I: So you receive your money in cash then save it on your Account personally?**

R2: Yes

**I: Okay**

**Please stay well my dear friends.**

**I promise to come back and check on you please.**

ALL: Okay madam

**I: I bought for you some powdered milk. Hope you will like it!**

ALL: Thank you so much dear and may GOD reward you abundantly.

**I: Thank you too**
